# Associations of daily eating frequency and nighttime fasting duration with biological aging in National Health and Nutrition Examination Survey (NHANES) 2003–2010 and 2015–2018

**DOI:** 10.1186/s12966-024-01654-y

**Published:** 2024-09-19

**Authors:** Xuanyang Wang, Jia Zhang, Xiaoqing Xu, Sijia Pan, Licheng Cheng, Keke Dang, Xiang Qi, Ying Li

**Affiliations:** Department of Nutrition and Food Hygiene, School of Public Health, the National Key Discipline, Harbin Medical University, 157 Baojian Road, Harbin, 150081 P. R. China

**Keywords:** Daily eating frequency, Nighttime fasting duration, Biological aging, Predicted age metrics, National Health and Nutrition Examination Survey (NHANES)

## Abstract

**Background:**

Information on the influences of daily eating frequency (DEF) and nighttime fasting duration (NFD) on biological aging is minimal. Our study investigated the potential associations of DEF and NFD with accelerated aging.

**Methods:**

Out of 24212 participants in NHANES 2003–2010 and 2015–2018, 4 predicted age metrics [homeostatic dysregulation (HD), Klemera–Doubal method (KDM), phenoAge (PA), and allostatic load (AL)] were computed based on 12 blood chemistry parameters. Utilizing 24-h dietary recall, DEF was measured by the frequency of eating occurrences, while NFD was determined by assessing the timing of the initial and final meals throughout the day. Weighted multivariate linear regression models and restricted cubic spline (RCS) were utilized to examine the associations.

**Results:**

Compared to DEF of ≤ 3.0 times, subjects with DEF ≥ 4.6 times demonstrated lower KDM residual [β: -0.57, 95% confidence-interval (CI): (-0.97, -0.17)] and PA residual [β: -0.47, 95% CI: (-0.69, -0.25)]. In comparison to NFD between 10.1 and 12.0 h, individuals with NFD ≤ 10.0 h were at higher HD [β: 0.03, 95% CI: (0.01, 0.04)], KDM residual [β: 0.34, 95% CI: (0.05, 0.63)], and PA residual [β: 0.38, 95% CI: (0.18, 0.57)]. Likewise, those with NFD ≥ 14.1 h also had higher HD [β: 0.02, 95% CI: (0.01, 0.04)] and KDM residual [β: 0.33, 95% CI: (0.03, 0.62)]. The results were confirmed by the dose–response relationships of DEF and NFD with predicted age metrics. Lactate dehydrogenase (LDH) and globulin (Glo) were acknowledged as implicated in and mediating the relationships.

**Conclusions:**

DEF below 3.0 times and NFD less than 10.0 or more than 14.1 h were independently associated with higher predicted age metrics.

**Supplementary Information:**

The online version contains supplementary material available at 10.1186/s12966-024-01654-y.

## Background

Aging is the accumulation of life’s consequences, like molecular and cellular damage, causing a decline in function, chronic illnesses, and eventually death [1]. Across our lifespan, diverse factors such as age, genetic susceptibilities, environmental exposures, and lifestyle practices play a role in the accrual of damage [2]. Considering the significant aging of the global population, accelerated aging exposes adults to higher vulnerability to morbidity and mortality, resulting in rapidly rising healthcare costs associated with aging [3]. Implementing strategies focusing on multiple dimensions of daily life is crucial for managing the risks of aging.

Recently, a review has outlined how biological aging can be measured through twelve hallmarks such as epigenetic modifications, proteomic profiles, metabolomic markers, and composite clinical parameter algorithms [4]. Among those, the combined clinical-parameter algorithms have been demonstrated the ability to accurately predict chronological age (CA) [5]. Such predictions delineate the internal aging mechanisms, indicating the real health and functional condition of the body [6]. Utilizing the Mahalanobis distance metric, homeostatic dysregulation (HD) quantified the deviation between an individual’s clinical measurements and the reference set by a young, healthy population [7]. The measurement of Klemera–Doubal method (KDM) determining the deterioration of the body was made possible by performing regression analyses between specific biomarkers and chronological age in the reference population [8]. By utilizing elastic-net Gompertz regression, phenoAge (PA) was calculated by examining various factors associated with mortality risks to offer an estimate of the likelihood of death [9]. The assessment of allostatic load (AL) involved the measurement of biomarker levels indicating increased disease risks, capturing the cumulative impact of chronic stress and life events [10]. People sharing the same CA may undergo different biological aging stages and susceptibility to morbidity and mortality [11]. Uncovering the causes of accelerating aging is essential for developing interventions to decelerate biological aging, and prolong both health span and lifespan.

Chrono-nutrition, an emerging field of nutritional science, investigates the effects of circadian eating behaviors on health, emphasizing the significance of the rhythms of food consumption in addition to its quantity and quality for health [12]. Nutritional challenges reshape the circadian clock, whereas timing-specific food consumption has been demonstrated to deeply impact physiology [13]. Increasing evidence indicated that disruptions in circadian rhythms and mistimed eating, including skipping breakfast, consuming high-energy meals during dinner, and eating late at night detrimentally affected health [13–16].

The progression of aging is a multifactorial process, with diet playing an important role [17]. The impact of diet is extensive. Opting for healthy eating habits could contribute to improving energy metabolism, maintaining metabolic balance, reducing oxidative stress, and preventing abnormal inflammatory reactions [18–20]. Serum lactate dehydrogenase (LDH) is a key oxidoreductase enzyme in glycolysis, commonly increased during inflammation [21]. Increased LDH and its mRNA suggest that glucose indirectly affects tissue tricarboxylic acid cycle (TCA cycle) metabolism through circulating lactate, except in the brain [22]. Globulin (Glo), a diverse protein group, may indicate inflammation and oxidative stress due to its roles in matrix repair and immune regulation [23]. Findings from animal experiments suggest that urinary globulin in rats peaks with their feeding cycle, and fasting exacerbates its reduction via mRNA suppression [24]. Consequently, the two indicators listed are considered as potential mediators in our study’s associations.

Recently, findings from a cohort study comprising 30464 adults demonstrated that both reduced daily eating frequency (DEF) and shortening or lengthening nighttime fasting duration (NFD) were independently related to higher risk of cardiovascular and all-cause mortality [25]. This may be explained by the relationship between lower DEF and higher blood pressure and serum cholesterol levels [26], alongside the associations of longer NFD with improvements in body weight [27], insulin sensitivity [28], and inflammation [29, 30]. Nonetheless, there is limited research on whether DEF and NFD, two features of circadian eating behaviors, are related to the biological aging process. Here, we examined how DEF and NFD were associated with four projected age metrics (HD, KDM, PA, and AL) based on National Health and Nutrition Examination Survey (NHANES) 2003–2010 and 2015–2018.

## Methods

### Study population

**Fig. 1:**
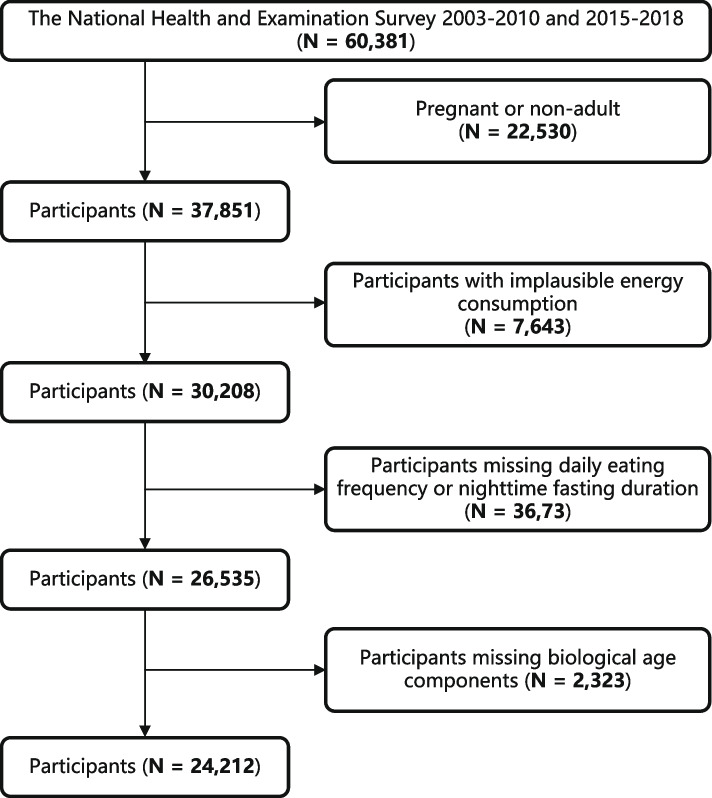
The gradual selection process of participants from the NHANES 2003–2010 and 2015–2018

The NHANES program is a continuous, nationwide study that focus on gathering information related to the health, nutrition, and lifestyle of the general population in the United States. It is conducted by the National Center for Health Statistics within the Centers for Disease Control and Prevention (CDC) and has been ongoing since the 1960s. The program has been approved by the National Center for Health Statistics Research Ethics Review Board and undergoes annual assessments (ethics approval number: Protocol #98–12, Protocol #2005–06, Continuation of Protocol #2005–06, Continuation of Protocol #2011–17, and Protocol #2018–01). Data collection methods, including interviews, physical examinations, and laboratory testing, are performed either at mobile examination centers (MECs) or in individuals’ homes [31]. The cross-sectional data utilized for our analysis was obtained from NHANES 2003–2010 and 2015–2018, involving 24212 participants who met the specified criteria: adults not in a pregnant state (*n* = 37851), standard energy consumption (the recommended daily energy intake of 800 to 4200 kcal for males and 500 to 3500 kcal for females) (*n* = 30208) [32], possess complete information on DEF and NFD (*n* = 26535), all components of predicted age metrics and covariates are comprehensive (*n* = 24212) (Fig. 1).

### Assessment of exposure

Data on food consumption over two nonconsecutive days was captured through two 24-h dietary recall interviews, as outlined by the guidelines from the U.S. Department of Agriculture's Food and Nutrient Database for Dietary Studies [33]. The first interview was done face-to-face, followed by the second interview took place via telephone 3–10 days later. Participants were asked to provide the consumption time for each food and drink during the interviews. Eating episodes were characterized by the number of occurrences in which calorie-containing foods or drinks were consumed, with a conservative threshold of 50 kcal set for identifying each eating episode [26]. The mean DEF was computed by averaging the eating episodes recorded over the two days. The calculation for the mean NFD utilized the formula: 24 h minus the time of last meal plus the time of the first meal. To provide an example, if a participant’s first eating time was 8:00 a.m. and his/her last eating time was 8:00 p.m., the NFD would be 24 minus 20 plus 8, resulting in 12 h of fasting.

### Assessment of main outcomes

By employing a selected group of 12 blood chemistry parameters (Supplementary Table 1—2), HD, KDM, and PA were determined through the use of the most reputable algorithms, initially standardized with NHANES 1988–1994 (NHANES III) and originally detailed by Nakazato et al. [34], Klemera et al. [35], and Levine et al. [36], correspondingly. The relevant code could be accessed via the R package “BioAge” at https://github.com/dayoonkwon/BioAge. In our research, we designated individuals as being at risk by allocating them to the top quartile for eleven biomarkers, with the exception of albumin, for which individuals in the lowest quartile were identified as being at risk according to previous study [37]. The AL, ranging from 0 to 1, represented the proportion of biomarkers designated as “at risk”.

The residual differences between predicted age metrics and chronological age were termed age accelerations (AAs), which harmonized inconsistencies across the measurement platforms for each component of predicted age metrics [38, 39]. To determine the AAs, residual values were calculated through a linear regression analysis with either KDM or PA as the independent variable and chronological age as the dependent variable [40]. Residuals were not calculated for HD and AL, as they were not considered age metrics and were factored in deviations from a reference population [8, 41]. With HD being significantly skewed in our study, we transformed HD using the natural logarithm transformation and used it as the outcome in models predicting HD age acceleration. Higher levels of HD, KDM residuals, PA residuals, and AL suggest accelerated aging [42–44], with subsequent analyses emphasizing these variables as primary outcomes.

### Assessment of mediation variables

Trained phlebotomists at NHANES MECs collected blood samples without requiring fasting. Participants were allocated randomly to morning, afternoon, or evening sessions, with all samples being refrigerated and transferred to Collaborative Laboratory Services for analysis. LDH was determined using the Beckman Synchron LX20 with LDH reagent through an enzymatic rate method. Within our study, following the same approach as the previous investigation, only subjects with normal LDH levels (105–333 IU/L) were included [45]. Serum Glo is a set of proteins responsible for transporting diverse substances and participating in defense mechanisms in the body [46], with levels ranging from 14–65 g/L [47]. Additional details about the LDH and Glo analysis techniques are provided in the NHANES Laboratory/Medical Technician Procedures Manual (LPM) [48].

### Assessment of covariates

Confounders considered in the present analysis were age (years), sex (male/female), race [Mexican American/other Hispanic/non-Hispanic White/non-Hispanic Black/others (including Asians or multiracial)], NHANES cycle (year), body mass index (BMI, kg/m^2^), smoking (yes/no), drinking (yes/no), exercise (yes/no), education (below high school/high school/above high school), annual family income (≤ $55,000/ > $55,000), sleep duration (hours), shift work (yes/no), daily energy intake (kcal/d), nutrient supplement use (yes/no), healthy eating index-2015 (HEI-2015), weekend dietary survey data (yes/no), self-reported cancer (yes/no), cardiovascular diseases (CVD) (yes/no), hypertension (yes/no), and diabetes (yes/no). The calculation of BMI was obtained by dividing weight in kilograms by the square of height in meters. The metabolic equivalent scores for weekly recreational activities was used to assess physical activity, with regular exercise defined as participating in a minimum of 150 min of moderate to high-intensity physical activity per week [49]. The HEI-2015 serves as a summary measure of compliance with the USDA 2015–2020 Dietary Guidelines for Americans [50], assessing the consumption of total fruits, whole fruits, total vegetables, greens and beans, total protein food, seafood and plant protein, whole grain, dairy, fatty acids, refined grain, sodium, added sugar, and saturated fat.

### Statistical analysis

Referring to NHANES analytic guidelines guaranteed thorough consideration of sample weights, stratification, and clustering to account for the complex survey design, with the inclusion of these factors in all analyses. Demographic features, disease prevalence, and anthropometric measurements were depicted as means (95% CI) for continuous variables and percentages (n) for categorical variables, which were analyzed utilizing general linear regression and logistic regression. Categorization into quintiles was applied to the DEF and NFD. Multivariate linear regression models were utilized to analyze the associations of DEF and NFD with HD, KDM residuals, PA residuals, and AL. Assumptions were examined using the Durbin-Watson test, scatter plots, correlation coefficients, tolerances, variance inflation factor (VIF), and Q-Q plots, and no violations were observed. Visualization of the dose–response relationships between DEF and NFD and predicted age metrics was displayed using restricted cubic spline (RCS) with 3 knots at the 10th, 50th, and 90th percentiles [51]. Mediation analysis using mediation package (version 4.5.0) was performed to evaluate the mediation effects of LDH and Glo in the associations. The following factors were used to conduct multiple stratified analyses to investigate potential modifying impacts: age (≤ 60 years/ > 60 years), sex (male/female), race (non-Hispanic white/others), BMI (< 30 kg/m^2^/ ≥ 30 kg/m^2^), smoking (yes/no), drinking (yes/no), exercise (yes/no), education (above high school/others), income (≤ $55,000/ > $55,000), sleep duration (≤ 7 h/ > 7 h), daily energy intake (male, ≤ 2500 kcal/ > 2500 kcal and female, ≤ 2000 kcal/ > 2000 kcal) [52], HEI-2015 (< 30.53/ ≥ 30.53), dietary supplements use (yes/no), NFD (< 12.58 h/ ≥ 12.58 h), and DEF (< 4 times/ ≥ 4 times). Statistical analyses were performed using R 4.1.1, with statistical significance set at a two-sided *P* < 0.05.

## Results

### Baseline characteristics

The demographic characteristics, disease prevalence, and anthropometric measurements of participants stratified by quintiles of DEF and NFD were shown in Table 1 and Supplementary Table 3. Participants in quintile 1, with an eating frequency of no more than 3.0 times, were more likely to be younger, non–drinkers, non-regular exercisers, and night shift workers; have higher BMI, NFD, Glo, HD, KDM residual, PA residual, and AL; as well as lower education, annual household income, daily energy intake, HEI-2015, and dietary supplements use when compared to those in quintiles 2 to 5, where DEF exceeded 3.0 times (Table 1). Participants in quintile 1, with an NFD of no more than 10.0 h, were more likely to be younger, male, non-Hispanic white, smokers, drinkers, regular exercisers, and night shift workers; have lower BMI, sleep duration, Glo, HD, KDM, and PA; as well as higher education, daily energy intake, DEF, LDH, and PA residual when compared to those with an NFD exceeding 10.0 h (quintiles 2 to 5) (Supplementary Table 3).

**Table 1:** Differences in the baseline characteristics of participants categorized by quintiles of DEF (*n* = 24,212)^a^

		Daily eating frequency (DEF)						
	Overall	Q1 (≤ 3.0 times)	Q2 (3.1—3.5 times)	Q3 (3.6—4.0 times)	Q4 (4.1—4.5 times)	Q5 (≥ 4.6 times)	*P*	*P*_test_
		*N* = 5180	*N* = 4262	*N* = 4544	*N* = 3891	*N* = 6335		
Age, years	49.09 (48.85, 49.33)	47.60 (47.06, 48.14)	49.49 (48.90, 50.08)	50.21 (49.65, 50.77)	49.38 (48.79, 49.97)	49.06 (48.62, 49.49)	< 0.001	< 0.001
Male, n (%)	11,751 (48.5)	2500 (48.3)	2082 (48.9)	2230 (49.1)	1870 (48.1)	3069 (48.4)	0.873	0.871
Non-Hispanic white, n (%)	11,171 (46.1)	2015 (38.9)	1832 (43.0)	2134 (47.0)	1860 (47.8)	3330 (52.6)	< 0.001	0.040
Body mass index, kg/m^2^	29.02 (28.93, 29.10)	29.86 (29.66, 30.05)	29.22 (29.01, 29.42)	29.12 (28.92, 29.31)	28.79 (28.58, 29.00)	28.26 (28.11, 28.42)	< 0.001	< 0.001
Current smoking, n (%)	10,504 (43.4)	2245 (43.3)	1816 (42.6)	1954 (43.0)	1692 (43.5)	2797 (44.2)	< 0.001	0.081
Current drinking, n (%)	16,329 (67.4)	3232 (62.4)	2795 (65.6)	3107 (68.4)	2688 (69.1)	4507 (71.1)	< 0.001	0.001
Regular exercise, n (%)	8245 (34.1)	1576 (30.4)	1364 (32.0)	1520 (33.5)	1358 (34.9)	2427 (38.3)	< 0.001	< 0.001
Above high school, n (%)	12,116 (50.0)	2133 (41.2)	1960 (46.0)	2311 (50.9)	2096 (53.9)	3616 (57.1)	< 0.001	< 0.001
> 55,000 annual household income, n (%)	8367 (34.6)	1525 (29.4)	1343 (31.5)	1520 (33.5)	1403 (36.1)	2576 (40.7)	< 0.001	< 0.001
Sleep duration, hours	7.29 (7.26, 7.32)	7.35 (7.26, 7.43)	7.28 (7.22, 7.34)	7.33 (7.25, 7.41)	7.23 (7.16, 7.31)	7.24 (7.17, 7.30)	0.113	0.054
Night shift work, n (%)	711 (2.9)	172 (3.3)	110 (2.6)	127 (2.8)	121 (3.1)	181 (2.9)	< 0.001	< 0.001
Daily energy intake, kcal/d	1992.76 (1983.76, 2001.76)	1619.27 (1601.75, 1636.79)	1841.20 (1821.70, 1860.71)	1984.57 (1965.14, 2004.00)	2116.15 (2094.87, 2137.44)	2330.23 (2313.22, 2347.23)	< 0.001	< 0.001
Healthy eating index-2015	31.11 (31.00, 31.22)	29.86 (29.63, 30.09)	30.45 (30.20, 30.70)	30.96 (30.71, 31.21)	31.39 (31.12, 31.67)	32.53 (32.31, 32.74)	< 0.001	< 0.001
Dietary supplements use, n (%)	12,226 (50.5)	2151 (41.5)	2056 (48.2)	2293 (50.5)	2066 (53.1)	3660 (57.8)	< 0.001	< 0.001
Dietary data surveyed on weekend, n (%)	1147 (4.7)	285 (5.5)	192 (4.5)	208 (4.6)	202 (5.2)	260 (4.1)	0.005	0.106
Self-reported cancer, n (%)	2315 (9.6)	432 (8.3)	406 (9.5)	469 (10.3)	384 (9.9)	624 (9.9)	< 0.001	< 0.001
Self-reported hypertension, n (%)	8424 (34.8)	1802 (34.8)	1515 (35.5)	1675 (36.9)	1346 (34.6)	2086 (32.9)	< 0.001	0.288
Self-reported cardiovascular diseases, n (%)	2581 (10.7)	580 (11.2)	475 (11.1)	557 (12.3)	419 (10.8)	550 (8.7)	< 0.001	< 0.001
Self-reported diabetes, n (%)	2956 (12.2)	683 (13.2)	570 (13.4)	594 (13.1)	452 (11.6)	657 (10.4)	< 0.001	0.694
Daily eating frequency, times	4.13 (4.12, 4.14)	2.73 (2.72, 2.74)	3.50 (3.50, 3.50)	4.00 (4.00, 4.00)	4.50 (4.50, 4.50)	5.57 (5.55, 5.59)	< 0.001	< 0.001
Nighttime fasting duration, hours	12.72 (12.69, 12.76)	15.21 (15.14, 15.28)	13.25 (13.19, 13.31)	12.49 (12.44, 12.55)	11.91 (11.84, 11.97)	11.00 (10.95, 11.05)	< 0.001	< 0.001
Lactate dehydrogenase, U/L	135.23 (134.80, 135.66)	135.93 (134.93, 136.92)	135.19 (134.21, 136.17)	135.55 (134.47, 136.62)	134.49 (133.53, 135.45)	134.92 (134.07, 135.77)	0.307	0.650
Globulin, g/dL	2.96 (2.95, 2.96)	3.02 (3.00, 3.03)	2.96 (2.95, 2.98)	2.96 (2.95, 2.97)	2.95 (2.93, 2.96)	2.91 (2.90, 2.92)	< 0.001	< 0.001
Homeostatic dysregulation	1.68 (1.68, 1.69)	1.76 (1.74, 1.78)	1.73 (1.71, 1.75)	1.70 (1.67, 1.72)	1.64 (1.62, 1.67)	1.61 (1.59, 1.63)	< 0.001	< 0.001
Klemera-Doubal method, years	41.98 (41.76, 42.20)	41.82 (41.31, 42.34)	42.65 (42.09, 43.20)	42.91 (42.38, 43.43)	41.91 (41.36, 42.45)	41.04 (40.63, 41.44)	< 0.001	0.001
Klemera-Doubal method residual, years	0.00 (-0.10, 0.10)	1.34 (1.11, 1.57)	0.24 (-0.02, 0.49)	-0.23 (-0.47, 0.01)	-0.36 (-0.61, -0.11)	-0.88 (-1.06, -0.69)	< 0.001	< 0.001
PhenoAge, years	48.33 (48.07, 48.59)	47.46 (46.88, 48.04)	48.96 (48.33, 49.60)	49.49 (48.88, 50.09)	48.41 (47.78, 49.04)	47.73 (47.27, 48.20)	< 0.001	< 0.001
PhenoAge residual, years	0.01 (-0.05, 0.07)	0.72 (0.58, 0.86)	0.18 (0.04, 0.33)	-0.07 (-0.21, 0.07)	-0.20 (-0.35, -0.06)	-0.49 (-0.61, -0.38)	< 0.001	< 0.001
Allostatic load	0.28 (0.28, 0.29)	0.30 (0.29, 0.30)	0.29 (0.28, 0.29)	0.29 (0.28, 0.29)	0.28 (0.28, 0.29)	0.27 (0.27, 0.27)	< 0.001	< 0.001

^a^Continuous variables were presented as mean (95% CI). Categorical variables were listed as N (%). And *P*_test_ was the result of Bonfreni correction

In terms of the different quantitative aging measures examined in the present study, participants’ predicted age metrics were closely correlated with their chronological ages (Supplementary Fig. 1).

### Associations of DEF and NFD with predicted age metrics

**Fig. 2:**
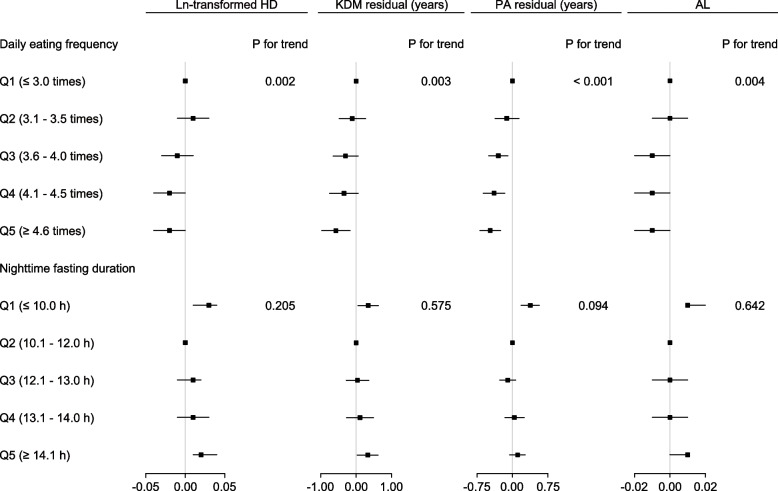
Forest plot of the associations of DEF and NFD with predicted age metrics. The adjustments included age, sex, race, NHANES cycle, BMI, smoking, drinking, exercise, education, income, sleep duration, shift work, daily energy intake, nutrient supplement use, HEI-2015, dietary data surveyed on weekend, self-reported cancer, CVD, hypertension, and diabetes. Models for DEF and NFD were additionally adjusted for NFD and DEF, respectively. Q, quintile

Compared to those in the lowest quintile (≤ 3.0 times), subjects in the highest quintile of DEF (≥ 4.6 times) demonstrated lower KDM residual [β: -0.57, 95% CI: (-0.97, -0.17)] and PA residual [β: -0.47, 95% CI: (-0.69, -0.25)] (Fig. 2, Supplementary Table 4). Unlike DEF, the second quintile (10.1 – 12.0 h) of NFD was defined as the group of reference. According to the weighted beta and 95% CIs, individuals in the lowest quintile (≤ 10.0 h) were at higher HD [β: 0.03, 95% CI: (0.01, 0.04)], KDM residual [β: 0.34, 95% CI: (0.05, 0.63)], and PA residual [β: 0.38, 95% CI: (0.18, 0.57)]. Likewise, those in the top 20% (≥ 14.1 h) had higher HD [β: 0.02, 95% CI: 0.01, 0.04)] and KDM residual [β: 0.33, 95% CI: (0.03, 0.62)] (Fig. 2, Supplementary Table 5).

### Dose–response relationships between DEF and NFD and predicted age metrics

**Fig. 3:**
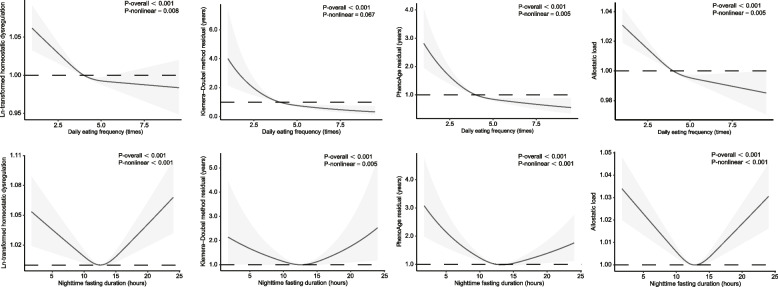
Smoothing curve for the associations of DEF and NFD with predicted age metrics. Multivariate linear regression models and RCS were performed with adjusting for age, sex, race, NHANES cycle, BMI, smoking, drinking, exercise, education, income, sleep duration, shift work, daily energy intake, nutrient supplement use, HEI-2015, dietary data surveyed on weekend, self-reported cancer, CVD, hypertension, and diabetes. Models for DEF and NFD were additionally adjusted for NFD and DEF, respectively. Examination of the linear or nonlinear relationship of the spline was conducted through the use of the analysis of variance (ANOVA). The solid black lines correspond to the exponential transformed central estimates, and the gray-shaded regions indicate the 95% confidence intervals

There was a significant negative correlation between DEF and NFD (*r* = -0.59, *P* < 0.001) (Supplementary Fig. 2). With the exclusion of KDM residual (*P*_overall_ < 0.001, *P*_nonlinearity_ = 0.067), linear relationships were observed between DEF and HD (*P*_overall_ < 0.001, *P*_nonlinearity_ = 0.028), PA residual (*P*_overall_ < 0.001, *P*_nonlinearity_ = 0.005), and AL (*P*_overall_ < 0.001, *P*_nonlinearity_ = 0.005). The predicted age metrics consistently decreased as DEF increased, reaching a beta estimate of 0.0 at around 4.0 times per day. Concerning NFD, HD (*P*_overall_ < 0.001, *P*_nonlinearity_ = 0.001), KDM residual (*P*_overall_ < 0.001, *P*_nonlinearity_ = 0.005), PA residual (*P*_overall_ < 0.001, *P*_nonlinearity_ < 0.001), and AL (*P*_overall_ < 0.001, *P*_nonlinearity_ < 0.001) all exhibited a gradual decrease as NFD extended to 10–14 h per day. Subsequent increases in NFD led to a gradual increase of the predicted age metrics, demonstrating robust U-shaped relationships (all *P*_nonlinearity_ < 0.01) (Fig. 3).

### Effects mediated by LDH and Glo on the associations of DEF and NFD with predicted age metrics

**Fig. 4:**
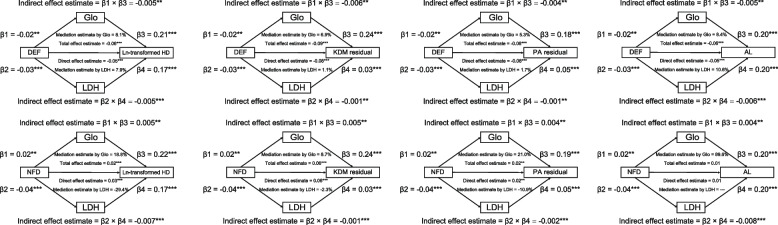
Effects mediated by Glo and LDH on the associations of DEF and NFD with predicted age metrics. The results were presented as standardized regression coefficients after adjusting for the covariates in the full model of multivariate linear regression models. *P < 0.05, **P < 0.01, ***P < 0.001

Our findings illustrated the significant effects mediated by LDH and Glo on the associations of DEF and NFD with predicted age metrics (Fig. 4). Statistical analysis using standardized regression coefficients showed the total effects of DEF on HD (β_Tot_ = -0.06, *P* < 0.001), KDM residual (β_Tot_ = -0.09, *P* < 0.001), PA residual (β_Tot_ = -0.08, *P* < 0.001), and AL (β_Tot_ = -0.06, *P* < 0.001). Correspondingly, the total effects of NFD on HD (β_Tot_ = 0.03, *P* < 0.001), KDM residual (β_Tot_ = 0.06, *P* < 0.001), and PA residual (β_Tot_ = 0.02, *P* < 0.001) were illustrated. For DEF, the indirect effects mediated by Glo and LDH contributed to a distinct portion of the total effects on HD (8.5, 7.3%), KDM residual (6.9, 1.1%), PA residual (5.3, 1.7%), and AL (8.4, 10.6%). Moreover, Glo and LDH-mediated indirect effects also contributed to the specific proportions of the total effects of NFD on HD (18.0, -24.7%), KDM residual (8.7, -2.3%), and PA residual (21.0, -10.9%).

### Associations of DEF and NFD with predicted age metrics stratified by the potential confounders

When conducting sensitivity analyses considering potential confounders, the associations of DEF with HD and PA residual and associations of DEF with KDM residual and PA residual differed according to age and daily energy intake, respectively (*P*_interaction_ < 0.05) (Supplementary Table 6—9). The associations between NFD and KDM residual and PA residual were similarly varying with age (*P*_interaction_ < 0.05) (Supplementary Table 10—13).

## Discussion

In this sizable, nationwide cross-sectional study of the representative American population, we demonstrated for the first time the associations of circadian eating patterns (DEF and NFD) with estimated biological aging using predicted age metrics. Showing a higher DEF (≥ 4.6 times) was associated with lower predicted age metrics. Either NFD shorter than 10.0 h or longer than 14.1 h were independently related to higher predicted age metrics. Furthermore, LDH and Glo partially mediated the impacts of DEF and NFD on biological aging. Sensitivity analyses revealed that the relationships differed according to age and daily energy intake.

The consistent results matched a previous study conducted among healthy, normal-weight, middle-aged adults without caloric restriction, which found that subjects consuming only one meal per day experienced significant increases in cardiovascular health markers [53]. Moreover, a prospective study of the representative American population demonstrated that DEF below 3 times was independently related to 33% and 16% higher risks of CVD and all-cause death [25]. Additionally, dietary behaviors featuring low daily energy intake may contribute to the observed relationships. In the present research, it was observed that individuals with DEF below 3.0 times and lower daily energy intake had higher predicted age metrics. This might result from insufficient energy intake, preventing the body from obtaining adequate nutrients for effective cell repair, or an irregular secretion of insulin and other hormones, impacting the body’s metabolism, immune and so forth [54, 55]. As an example, interventional investigations have further revealed that regular meal consumption improved fasting lipid, postprandial insulin profiles, and thermogenesis [56]. Our observation implied that even subjects with lower energy consumption should carefully consider the frequency and regularity to mitigate the risks of accelerated aging.

The NFD is another important circadian eating behavior besides DEF. Despite the consistent findings of both animal and human studies reporting beneficial health effects resulting from prolonged nighttime fasting, including anti-inflammation, weight loss, and improved metabolic diseases, this research has revealed a more intricate relationship [27, 57]. Particularly, either NFD shorter than 10.0 or longer than 14.1 h were independently related to higher predicted age metrics. These discoveries implied that the health effects of altering NFD were subtle and not entirely straightforward, responding to certain findings from prior research. Shortened NFD often accompanies nighttime eating, which has been shown to be positively associated with metabolic syndrome and its components, cancer, and coronary heart disease [58–60]. These relationships could be attributed to the disturbances in rhythmic insulin release, potentially serving as a fundamental mechanism behind them. A circadian network establishes daily feeding windows, largely aligning with the active phase, allowing synchronization of brain and peripheral organs to feeding time through rhythmic cues from metabolic hormones, nutrients, and neural inputs [12]. Typically, insulin secretion rhythmically increases during the day and decreases at night [61], but eating late at night may disturb this rhythm by directly triggering insulin release [58]. Misaligned insulin signaling, as evidenced in animal and cellular research, disturbed the circadian organization and clock gene expression, ultimately leading to metabolic disorders through elevated production of the PERIOD protein [62]. Additionally, our study found that longer NFD (≥ 14.1 h) was associated with higher predicted age metrics, while previous research showed the health benefits from NFD longer than 15 h [29, 30]. However, it is crucial to contextualize these studies. Some highlighting the health benefits of extended NFD have centered on subjects with metabolic dysfunction, normally with expert supervision and particular objectives, including weight reduction and glucose regulation, which could potentially account for the variations. Despite fasting’s potential to offer protection from disease on multiple occasions, a recent animal experiment demonstrated that extended fasting and subsequent refeeding had limitations, or at the very least, come with a cost [63]. In terms of mechanisms, over three thousand genes display a 12-h biological rhythm, controlling hormone homeostasis, whose ebbs and flows are essential for the regulation of metabolism and response to stresses [64]. Several research has indicated that prolonged NFD increased the circulating ghrelin during the night [65], which could be significant in the mechanism through which calorie restriction promotes longevity [66]. Moreover, fasting has been proven to significantly affect the immune system. Recent research in both mice and humans suggested that extended fasting and subsequent refeeding changed the immune response to infection by reshaping the distribution of leukocytes [67].

Another revelation was that LDH and Glo partially mediated the impacts of DEF and NFD on biological aging. LDH is a crucial enzyme for anaerobic glycolysis, and its expression increases with aging [68]. Experiments with animals indicated that overexpressing LDH in the brain and skeletal muscle increased glycolysis and reduced lifespan [68, 69]. Corresponding to the increases in LDH and its encoding mRNA during the dark period [70], glucose primarily affects tissue TCA cycle metabolism indirectly (via circulating lactate) everywhere but the brain [22]. Globulin is a broad category of proteins with various functions, such as immunoglobulins, structural proteins, and hormone carriers. Available data indicates that levels of globulin could serve as markers for inflammation and oxidative stress, considering its involvement in repairing the extracellular matrix and regulating immunity [23]. Rats fed ad libitum displayed diurnal fluctuations in urinary alpha2u-globulin (a subtype of globulin) excretion, reaching peaks between 8 p.m. and 8 a.m., corresponding to the rat’s feeding cycle. Fasting induces a significant decrease in the synthesis of this protein, and prolonged fasting exacerbates this reduction, partly attributable to selective transcriptional suppression of its mRNA [24]. However, further mechanisms may link circadian dietary habits to biological aging, necessitating examination in future investigations.

The subgroup analyses specifically emphasized the relationships differed according to age and daily energy intake. This stresses the significance of fostering healthy eating habits among younger individuals, influenced by factors such as increased nutritional requirements, higher metabolic rate, and the enduring effects of unhealthy dietary habits [71].

The key strengths of this research included the sizable sample size that provided a representative depiction of the Americans, the utilization of various predicted age metrics, and comprehensive data on diverse covariates and clinical endpoints. However, it is imperative to recognize certain limitations when interpreting our results. Firstly, our study was performed with a cross-sectional methodology, wherein both circadian eating behaviors and AAs were examined at the start of the study. Some misclassification might be unavoidable, and any alterations or continual dietary patterns could not be fully recorded. Restricted by the cross-sectional studies, the causation between DEF and NFD and accelerated aging could not be ascertained based on the present results. Additionally, DEF represents the frequency of eating occasions that combine both meals and snacks. Given this definition, clinical interpretation should be careful. Moreover, the lack of consecutive 24-h dietary information made it necessary to determine NFD using the first and last time points, preventing the accurate estimation. Lastly, despite our efforts to adjust for potential confounders associated with circadian eating behaviors such as night shift work and sleep duration, there remains the possibility of other unreported confounding factors.

Dietary modifications, as simple yet feasible interventions, have been demonstrated to offer numerous health benefits [72]. Modifications in portion sizes and meal timings have become potent strategies to improve health and postpone the onset of diseases while decelerating aging [73, 74]. Our study also discovered that apart from dietary quantity and quality [32], both reduced DEF and shortening or lengthening NFD were independently associated with accelerated aging. Currently, an increasing number of both domestic and foreign health management guidelines and dietary recommendations are strongly advocating to emphasize circadian eating behaviors [75]. Professionals are advised to take note of our latest discoveries concerning the potential benefits of regular eating habits on the aging process. Moreover, future research endeavors are supposed to focus on integrating dietary quantity, quality, meal frequency, and fasting duration to develop strategies for preventing, postponing, and treating age-related chronic diseases.

## Conclusions

Results from this substantial nationwide cross-sectional study illustrated the associations between DEF below 3.0 times and NFD less than 10.0 or more than 14.1 h and the predicted age metrics, with LDH and Glo acting as mediators, especially notable in non-elderly populations. Subsequent studies are essential to validate our discoveries, involving larger prospective cohort studies, diverse racial and ethnic demographics, and exploration of underlying mechanisms and causation of the associations between circadian eating behaviors and biological aging.

## Supplementary Information

Additional file 1.

Additional file 2.

Additional file 3: Supplementary Table 1. Components of biological age metrics by quintiles of DEF. Supplementary Table 2. Components of biological age metrics by quintiles of NFD. Supplementary Table 3. Differences in the baseline characteristics of participants categorized by quintiles of NFD. Supplementary Table 4. Associations of DEF with predicted age metrics. Supplementary Table 5. Associations of NFD with predicted age metrics. Supplementary Table 6. Association of DEF with Ln-transformed HD stratified by variables of interest. Supplementary Table 7. Association of DEF with KDM residual stratified by variables of interest. Supplementary Table 8. Association of DEF with PA residual stratified by variables of interest. Supplementary Table 9. Association of DEF with AL stratified by variables of interest. Supplementary Table 10. Association of NFD with Ln-transformed HD stratified by variables of interest. Supplementary Table 11. Association of NFD with KDM residual stratified by variables of interest. Supplementary Table 12. Association of NFD with PA residual stratified by variables of interest. Supplementary Table 13. Association of NFD with AL stratified by variables of interest. Supplementary Fig. 1. Matrix displaying the associations between chronological age and predicted age metrics (Pearson correlation). Supplementary Fig. 2. Scatter plot illustrating the correlation between DEF and NFD (Pearson correlation).

## Data Availability

The data could be collected from openly available resources at https://www.cdc.gov/nchs/nhanes/index.htm.

## Abbreviations

AAs: Age accelerations
HEI-2015: Healthy eating index-2015
AL: Allostatic load
BMI: Body mass index
CA: Chronological age
CDC: Centers for Disease Control and Prevention
CI: Confidence-interval
CVD: Cardiovascular diseases
DEF: Daily eating frequency
Glo: Globulin
HD: Homeostatic dysregulation
KDM: Klemera–Doubal method
LDH: Lactate dehydrogenase
LPM: Laboratory/Medical Technician Procedures Manual
MECs: Mobile examination centers
NFD: Nighttime fasting duration
NHANES: National Health and Nutrition Examination Survey
PA: PhenoAge
RCS: Restricted cubic spline
TCA cycle: Tricarboxylic acid cycle

## References

[1] Moqri M, Herzog C, Poganik JR, Justice J, Belsky DW, Higgins-Chen A, et al. Biomarkers of aging for the identification and evaluation of longevity interventions. Cell. 2023;186(18):3758–75. 37657418 10.1016/j.cell.2023.08.003 PMC11088934

[2] Gladyshev VN, Kritchevsky SB, Clarke SG, Cuervo AM, Fiehn O, de Magalhães JP, et al. Molecular Damage in Aging. Nat Aging. 2021;1(12):1096–106. 36846190 10.1038/s43587-021-00150-3 PMC9957516

[3] Kennedy BK, Berger SL, Brunet A, Campisi J, Cuervo AM, Epel ES, et al. Geroscience: linking aging to chronic disease. Cell. 2014;159(4):709–13. 25417146 10.1016/j.cell.2014.10.039 PMC4852871

[4] López-Otín C, Blasco MA, Partridge L, Serrano M, Kroemer G. Hallmarks of aging: An expanding universe. Cell. 2023;186(2):243–78. 36599349 10.1016/j.cell.2022.11.001

[5] Li X, Ploner A, Wang Y, Magnusson PK, Reynolds C, Finkel D, et al. Longitudinal trajectories, correlations and mortality associations of nine biological ages across 20-years follow-up. eLife. 2020;9:e51507. 10.7554/eLife.51507 PMC7012595 32041686

[6] Cui F, Tang L, Li D, Ma Y, Wang J, Xie J, et al. Early-life exposure to tobacco, genetic susceptibility, and accelerated biological aging in adulthood. Sci Adv. 2024;10(18):eadl3747. 38701212 10.1126/sciadv.adl3747 PMC11068008

[7] Mahalanobis distance. In: The Concise Encyclopedia of Statistics. New York: Springer; 2008. pp. 325–6.

[8] Kwon D, Belsky DW. A toolkit for quantification of biological age from blood chemistry and organ function test data: BioAge. GeroScience. 2021;43(6):2795–808. 34725754 10.1007/s11357-021-00480-5 PMC8602613

[9] Levine ME. Modeling the rate of senescence: can estimated biological age predict mortality more accurately than chronological age? J Gerontol A Biol Sci Med Sci. 2013;68(6):667–74. 23213031 10.1093/gerona/gls233 PMC3660119

[10] McEwen BS, Stellar E. Stress and the individual. Mechanisms leading to disease. Arch Inter Med. 1993;153(18):2093–101. 8379800

[11] Lowsky DJ, Olshansky SJ, Bhattacharya J, Goldman DP. Heterogeneity in healthy aging. J Gerontol A Biol Sci Med Sci. 2014;69(6):640–9. 24249734 10.1093/gerona/glt162 PMC4022100

[12] Challet E. The circadian regulation of food intake. Nat Rev Endocrinol. 2019;15(7):393–405. 31073218 10.1038/s41574-019-0210-x

[13] Asher G, Sassone-Corsi P. Time for food: the intimate interplay between nutrition, metabolism, and the circadian clock. Cell. 2015;161(1):84–92. 25815987 10.1016/j.cell.2015.03.015

[14] Rong S, Snetselaar LG, Xu G, Sun Y, Liu B, Wallace RB, et al. Association of Skipping Breakfast With Cardiovascular and All-Cause Mortality. J Am Coll Cardiol. 2019;73(16):2025–32. 31023424 10.1016/j.jacc.2019.01.065

[15] Han T, Gao J, Wang L, Li C, Qi L, Sun C, et al. The Association of Energy and Macronutrient Intake at Dinner Versus Breakfast With Disease-Specific and All-Cause Mortality Among People With Diabetes: The U.S. National Health and Nutrition Examination Survey, 2003–2014. Diabetes Care. 2020;43(7):1442–8. 32354697 10.2337/dc19-2289

[16] Kelly KP, McGuinness OP, Buchowski M, Hughey JJ, Chen H, Powers J, et al. Eating breakfast and avoiding late-evening snacking sustains lipid oxidation. PLoS Biol. 2020;18(2): e3000622. 32108181 10.1371/journal.pbio.3000622 PMC7046182

[17] Simpson SJ, Le Couteur DG, Raubenheimer D, Solon-Biet SM, Cooney GJ, Cogger VC, et al. Dietary protein, aging and nutritional geometry. Ageing Res Rev. 2017;39:78–86. 28274839 10.1016/j.arr.2017.03.001

[18] Wallace M. Metabolic balance-a masterclass in mass action. Nat Metab. 2022;4(1):17–8. 35058632 10.1038/s42255-021-00522-4

[19] Arvanitakis M. Counting Calories, Hours, or Both: Is Time-restricted Eating Efficient for Weight Loss? Gastroenterology. 2022;163(3):775. 35738330 10.1053/j.gastro.2022.06.049

[20] Lowe DA, Wu N, Rohdin-Bibby L, Moore AH, Kelly N, Liu YE, et al. Effects of Time-Restricted Eating on Weight Loss and Other Metabolic Parameters in Women and Men With Overweight and Obesity: The TREAT Randomized Clinical Trial. JAMA Intern Med. 2020;180(11):1491–9. 32986097 10.1001/jamainternmed.2020.4153 PMC7522780

[21] Miao P, Sheng S, Sun X, Liu J, Huang G. Lactate dehydrogenase A in cancer: a promising target for diagnosis and therapy. IUBMB Life. 2013;65(11):904–10. 24265197 10.1002/iub.1216

[22] Hui S, Ghergurovich JM, Morscher RJ, Jang C, Teng X, Lu W, et al. Glucose feeds the TCA cycle via circulating lactate. Nature. 2017;551(7678):115–8. 29045397 10.1038/nature24057 PMC5898814

[23] Korovila I, Hugo M, Castro JP, Weber D, Höhn A, Grune T, et al. Proteostasis, oxidative stress and aging. Redox Biol. 2017;13:550–67. 28763764 10.1016/j.redox.2017.07.008 PMC5536880

[24] Driscoll HK, Crim MC, Zähringer J, Munro HN. Hepatic synthesis and urinary excretion of alpha2u-globulin by male rats: diurnal rhythm and response to fasting and refeeding. J Nutr. 1978;108(10):1691–701. 81296 10.1093/jn/108.10.1691

[25] Cheng W, Meng X, Gao J, Jiang W, Sun X, Li Y, et al. Relationship between circadian eating behavior (daily eating frequency and nighttime fasting duration) and cardiovascular mortality. The international journal of behavioral nutrition and physical activity. 2024;21(1):22. 38409117 10.1186/s12966-023-01556-5 PMC10895826

[26] St-Onge MP, Ard J, Baskin ML, Chiuve SE, Johnson HM, Kris-Etherton P, et al. Meal Timing and Frequency: Implications for Cardiovascular Disease Prevention: A Scientific Statement From the American Heart Association. Circulation. 2017;135(9):e96–121. 28137935 10.1161/CIR.0000000000000476 PMC8532518

[27] Liu D, Huang Y, Huang C, Yang S, Wei X, Zhang P, et al. Calorie Restriction with or without Time-Restricted Eating in Weight Loss. N Engl J Med. 2022;386(16):1495–504. 35443107 10.1056/NEJMoa2114833

[28] Wilkinson MJ, Manoogian ENC, Zadourian A, Lo H, Fakhouri S, Shoghi A, et al. Ten-Hour Time-Restricted Eating Reduces Weight, Blood Pressure, and Atherogenic Lipids in Patients with Metabolic Syndrome. Cell Metab. 2020;31(1):92–104.e5. 31813824 10.1016/j.cmet.2019.11.004 PMC6953486

[29] Cienfuegos S, Gabel K, Kalam F, Ezpeleta M, Wiseman E, Pavlou V, et al. Effects of 4- and 6-h Time-Restricted Feeding on Weight and Cardiometabolic Health: A Randomized Controlled Trial in Adults with Obesity. Cell Metab. 2020;32(3):366–78.e3. 32673591 10.1016/j.cmet.2020.06.018 PMC9407646

[30] Xie Z, Sun Y, Ye Y, Hu D, Zhang H, He Z, et al. Randomized controlled trial for time-restricted eating in healthy volunteers without obesity. Nat Commun. 2022;13(1):1003. 35194047 10.1038/s41467-022-28662-5 PMC8864028

[31] Johnson CL, Paulose-Ram R, Ogden CL, Carroll MD, Kruszon-Moran D, Dohrmann SM, et al. National health and nutrition examination survey: analytic guidelines, 1999–2010. Vital Health Stat 2. 2013;161:1–24. 25090154

[32] Hou W, Han T, Sun X, Chen Y, Xu J, Wang Y, et al. Relationship Between Carbohydrate Intake (Quantity, Quality, and Time Eaten) and Mortality (Total, Cardiovascular, and Diabetes): Assessment of 2003–2014 National Health and Nutrition Examination Survey Participants. Diabetes Care. 2022;45(12):3024–31. 36174119 10.2337/dc22-0462

[33] Bodner-Montville J, Ahuja JKC, Ingwersen LA, Haggerty ES, Enns CW, Perloff BP. USDA Food and Nutrient Database for Dietary Studies: Released on the web. J Food Compos Anal. 2006;19:S100–7.

[34] Nakazato Y, Sugiyama T, Ohno R, Shimoyama H, Leung DL, Cohen AA, et al. Estimation of homeostatic dysregulation and frailty using biomarker variability: a principal component analysis of hemodialysis patients. Sci Rep. 2020;10(1):10314. 32587279 10.1038/s41598-020-66861-6 PMC7316742

[35] Klemera P, Doubal S. A new approach to the concept and computation of biological age. Mech Ageing Dev. 2006;127(3):240–8. 16318865 10.1016/j.mad.2005.10.004

[36] Levine ME, Lu AT, Quach A, Chen BH, Assimes TL, Bandinelli S, et al. An epigenetic biomarker of aging for lifespan and healthspan. Aging. 2018;10(4):573–91. 29676998 10.18632/aging.101414 PMC5940111

[37] Shirazi TN, Hastings WJ, Rosinger AY, Ryan CP. Parity predicts biological age acceleration in post-menopausal, but not pre-menopausal, women. Sci Rep. 2020;10(1):20522. 33239686 10.1038/s41598-020-77082-2 PMC7689483

[38] Hägg S, Belsky DW, Cohen AA. Developments in molecular epidemiology of aging. Emerg Topics Life Sci. 2019;3(4):411–21. 10.1042/ETLS20180173 PMC7289014 33523205

[39] Horvath S, Raj K. DNA methylation-based biomarkers and the epigenetic clock theory of ageing. Nat Rev Genet. 2018;19(6):371–84. 29643443 10.1038/s41576-018-0004-3

[40] Mak JKL, McMurran CE, Kuja-Halkola R, Hall P, Czene K, Jylhävä J, et al. Clinical biomarker-based biological aging and risk of cancer in the UK Biobank. Br J Cancer. 2023;129(1):94–103. 37120669 10.1038/s41416-023-02288-w PMC10307789

[41] Graf GH, Crowe CL, Kothari M, Kwon D, Manly JJ, Turney IC, et al. Testing Black-White Disparities in Biological Aging Among Older Adults in the United States: Analysis of DNA-Methylation and Blood-Chemistry Methods. Am J Epidemiol. 2022;191(4):613–25. 34850809 10.1093/aje/kwab281 PMC9077113

[42] Chaney C, Wiley KS. The variable associations between PFASs and biological aging by sex and reproductive stage in NHANES 1999–2018. Environ Res. 2023;227: 115714. 36965790 10.1016/j.envres.2023.115714

[43] Obeng-Gyasi S, Elsaid MI, Lu Y, Chen JC, Carson WE, Ballinger TJ, et al. Association of Allostatic Load With All-Cause Mortality in Patients With Breast Cancer. JAMA Netw Open. 2023;6(5): e2313989. 37200034 10.1001/jamanetworkopen.2023.13989 PMC10196875

[44] Ye Q, Apsley AT, Etzel L, Hastings WJ, Kozlosky JT, Walker C, et al. Telomere length and chronological age across the human lifespan: A systematic review and meta-analysis of 414 study samples including 743,019 individuals. Ageing Res Rev. 2023;90: 102031. 37567392 10.1016/j.arr.2023.102031 PMC10529491

[45] Buckner SL, Loenneke JP, Loprinzi PD. Cross-Sectional Association Between Normal-Range Lactate Dehydrogenase, Physical Activity and Cardiovascular Disease Risk Score. Sports medicine (Auckland, NZ). 2016;46(4):467–72. 10.1007/s40279-015-0457-x 26694048

[46] Bonilla FA, Geha RS. 12. Primary immunodeficiency diseases. J Allergy Clin Immunol. 2003;111(2 Suppl):S571–81. 12592303 10.1067/mai.2003.86

[47] Huang J, Li R, Zhu H, Huang D, Li W, Wang J, et al. Association between serum globulin and cognitive impairment in older American adults. Front Public Health. 2023;11:1193993. 37670828 10.3389/fpubh.2023.1193993 PMC10476522

[48] Myers GL, Miller WG, Coresh J, Fleming J, Greenberg N, Greene T, et al. Recommendations for improving serum creatinine measurement: a report from the Laboratory Working Group of the National Kidney Disease Education Program. Clin Chem. 2006;52(1):5–18. 16332993 10.1373/clinchem.2005.0525144

[49] Wang X, Ma H, Gupta S, Heianza Y, Fonseca V, Qi L. The Joint Secular Trends of Sleep Quality and Diabetes Among US Adults, 2005–2018. J Clin Endocrinol Metab. 2022;107(11):3152–61. 35776011 10.1210/clinem/dgac401 PMC9681613

[50] Krebs-Smith SM, Pannucci TE, Subar AF, Kirkpatrick SI, Lerman JL, Tooze JA, et al. Update of the Healthy Eating Index: HEI-2015. J Acad Nutr Diet. 2018;118(9):1591–602. 30146071 10.1016/j.jand.2018.05.021 PMC6719291

[51] Desquilbet L, Mariotti F. Dose-response analyses using restricted cubic spline functions in public health research. Stat Med. 2010;29(9):1037–57. 20087875 10.1002/sim.3841

[52] Ho FK, Gray SR, Welsh P, Petermann-Rocha F, Foster H, Waddell H, et al. Associations of fat and carbohydrate intake with cardiovascular disease and mortality: prospective cohort study of UK Biobank participants. BMJ (Clinical research ed). 2020;368: m688. 32188587 10.1136/bmj.m688 PMC7190059

[53] Stote KS, Baer DJ, Spears K, Paul DR, Harris GK, Rumpler WV, et al. A controlled trial of reduced meal frequency without caloric restriction in healthy, normal-weight, middle-aged adults. Am J Clin Nutr. 2007;85(4):981–8. 17413096 10.1093/ajcn/85.4.981 PMC2645638

[54] Tao Z, Cheng Z. Hormonal regulation of metabolism-recent lessons learned from insulin and estrogen. Clin Sci (Lond). 2023;137(6):415–34. 36942499 10.1042/CS20210519 PMC10031253

[55] Hoffmann JP, Liu JA, Seddu K, Klein SL. Sex hormone signaling and regulation of immune function. Immunity. 2023;56(11):2472–91. 37967530 10.1016/j.immuni.2023.10.008

[56] Farshchi HR, Taylor MA, Macdonald IA. Beneficial metabolic effects of regular meal frequency on dietary thermogenesis, insulin sensitivity, and fasting lipid profiles in healthy obese women. Am J Clin Nutr. 2005;81(1):16–24. 15640455 10.1093/ajcn/81.1.16

[57] Di Francesco A, Di Germanio C, Bernier M, de Cabo R. A time to fast. Science (New York, NY). 2018;362(6416):770–5. 10.1126/science.aau2095 PMC8504313 30442801

[58] Yoshida J, Eguchi E, Nagaoka K, Ito T, Ogino K. Association of night eating habits with metabolic syndrome and its components: a longitudinal study. BMC Public Health. 2018;18(1):1366. 30537972 10.1186/s12889-018-6262-3 PMC6288903

[59] Zhang X, Wu Y, Na M, Lichtenstein AH, Xing A, Chen S, et al. Habitual Night Eating Was Positively Associated With Progress of Arterial Stiffness in Chinese Adults. J Am Heart Assoc. 2020;9(19): e016455. 32954888 10.1161/JAHA.120.016455 PMC7792372

[60] Cahill LE, Chiuve SE, Mekary RA, Jensen MK, Flint AJ, Hu FB, et al. Prospective study of breakfast eating and incident coronary heart disease in a cohort of male US health professionals. Circulation. 2013;128(4):337–43. 23877060 10.1161/CIRCULATIONAHA.113.001474 PMC3797523

[61] Boden G, Ruiz J, Urbain JL, Chen X. Evidence for a circadian rhythm of insulin secretion. Am J Physiol. 1996;271(2 Pt 1):E246–52. 8770017 10.1152/ajpendo.1996.271.2.E246

[62] Crosby P, Hamnett R, Putker M, Hoyle NP, Reed M, Karam CJ, et al. Insulin/IGF-1 Drives PERIOD Synthesis to Entrain Circadian Rhythms with Feeding Time. Cell. 2019;177(4):896–909.e20. 31030999 10.1016/j.cell.2019.02.017 PMC6506277

[63] Janssen H, Kahles F, Liu D, Downey J, Koekkoek LL, Roudko V, et al. Monocytes re-enter the bone marrow during fasting and alter the host response to infection. Immunity. 2023;56(4):783–96.e7. 36827982 10.1016/j.immuni.2023.01.024 PMC10101885

[64] Chahal HS, Drake WM. The endocrine system and ageing. J Pathol. 2007;211(2):173–80. 17200939 10.1002/path.2110

[65] Nunez-Salces M, Li H, Feinle-Bisset C, Young RL, Page AJ. The regulation of gastric ghrelin secretion. Acta Physiol (Oxf). 2021;231(3): e13588. 33249751 10.1111/apha.13588

[66] Amitani M, Amitani H, Cheng KC, Kairupan TS, Sameshima N, Shimoshikiryo I, et al. The Role of Ghrelin and Ghrelin Signaling in Aging. Int J Mol Sci. 2017;18(7):1511. 28704966 10.3390/ijms18071511 PMC5536001

[67] Jordan S, Tung N, Casanova-Acebes M, Chang C, Cantoni C, Zhang D, et al. Dietary Intake Regulates the Circulating Inflammatory Monocyte Pool. Cell. 2019;178(5):1102–14.e17. 31442403 10.1016/j.cell.2019.07.050 PMC7357241

[68] Hunt LC, Demontis F. Age-Related Increase in Lactate Dehydrogenase Activity in Skeletal Muscle Reduces Life Span in Drosophila. J Gerontol A Biol Sci Med Sci. 2022;77(2):259–67. 34477202 10.1093/gerona/glab260 PMC8824701

[69] Ross JM, Öberg J, Brené S, Coppotelli G, Terzioglu M, Pernold K, et al. High brain lactate is a hallmark of aging and caused by a shift in the lactate dehydrogenase A/B ratio. Proc Natl Acad Sci USA. 2010;107(46):20087–92. 21041631 10.1073/pnas.1008189107 PMC2993405

[70] Isobe Y, Hida H, Nishino H. Circadian rhythm of metabolic oscillation in suprachiasmatic nucleus depends on the mitochondrial oxidation state, reflected by cytochrome C oxidase and lactate dehydrogenase. J Neurosci Res. 2011;89(6):929–35. 21416482 10.1002/jnr.22609

[71] Pontzer H, Yamada Y, Sagayama H, Ainslie PN, Andersen LF, Anderson LJ, et al. Daily energy expenditure through the human life course. Science (New York, NY). 2021;373(6556):808–12. 10.1126/science.abe5017 PMC8370708 34385400

[72] Longo VD, Anderson RM. Nutrition, longevity and disease: From molecular mechanisms to interventions. Cell. 2022;185(9):1455–70. 35487190 10.1016/j.cell.2022.04.002 PMC9089818

[73] Le Couteur DG, Solon-Biet SM, Parker BL, Pulpitel T, Brandon AE, Hunt NJ, et al. Nutritional reprogramming of mouse liver proteome is dampened by metformin, resveratrol, and rapamycin. Cell Metab. 2021;33(12):2367–79.e4. 34767745 10.1016/j.cmet.2021.10.016

[74] Lee MB, Hill CM, Bitto A, Kaeberlein M. Antiaging diets: Separating fact from fiction. Science (New York, NY). 2021;374(6570):eabe7365. 10.1126/science.abe7365 PMC8841109 34793210

[75] Phillips JA. Dietary Guidelines for Americans, 2020–2025. Workplace Health Saf. 2021;69(8):395. 34279148 10.1177/21650799211026980

