# Supplementary figures and images for: Associations of daily eating frequency and nighttime fasting duration with biological aging in National Health and Nutrition Examination Survey (NHANES) 2003–2010 and 2015–2018
Source: Int J Behav Nutr Phys Act. 2024 Sep 19;21:104. doi: 10.1186/s12966-024-01654-y (PMC11414321; doi:10.1186/s12966-024-01654-y)

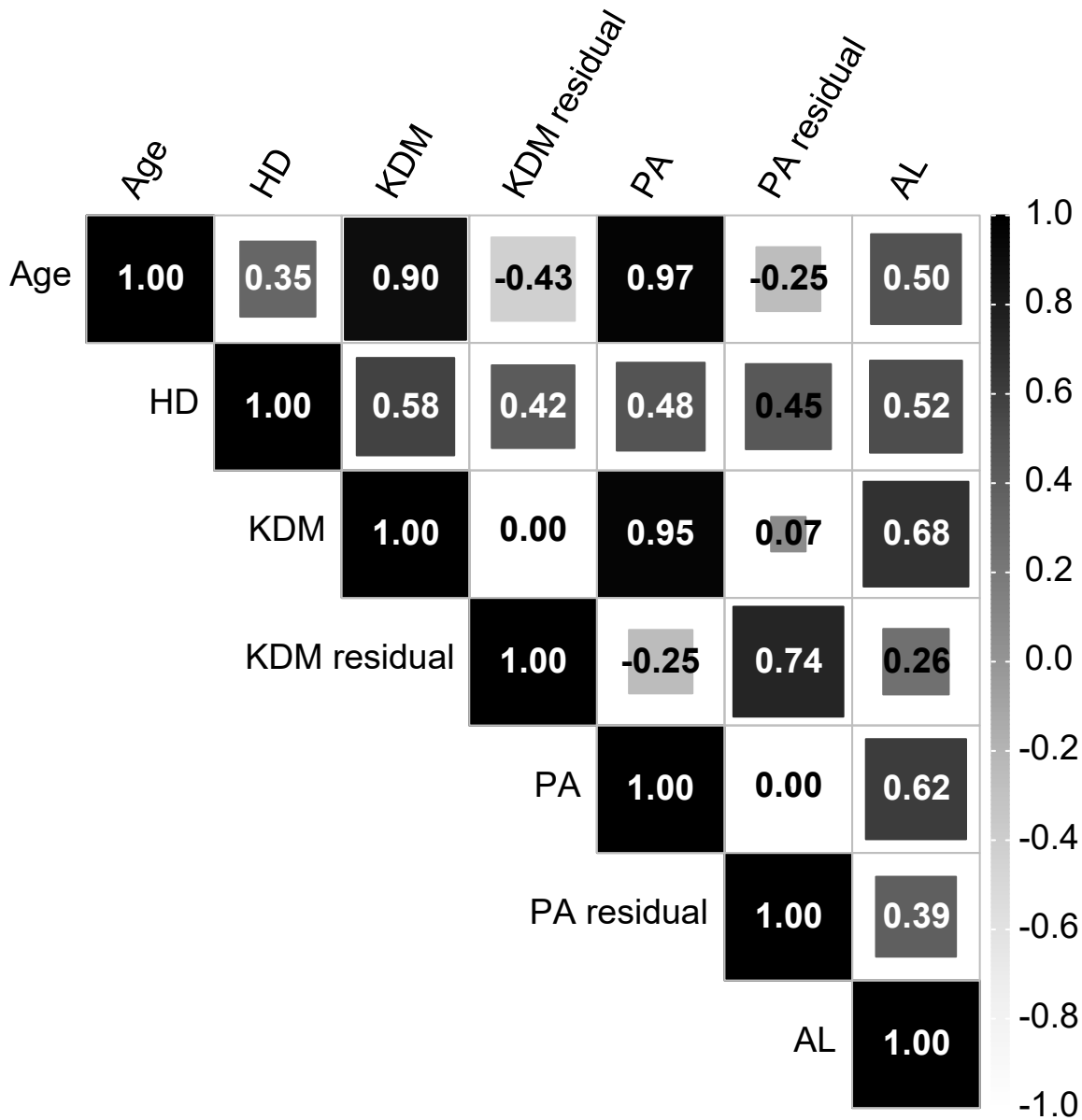

Supplement: Supplementary file 1 — Additional file 1. [file 12966_2024_1654_MOESM1_ESM.pdf]

$t_{\text{Student}}(24210) = -114.14, p < 0.01, \hat{r}_{\text{Pearson}} = -0.59, \text{CI}_{95\%} [-0.60, -0.58], n_{\text{pairs}} = 24212$

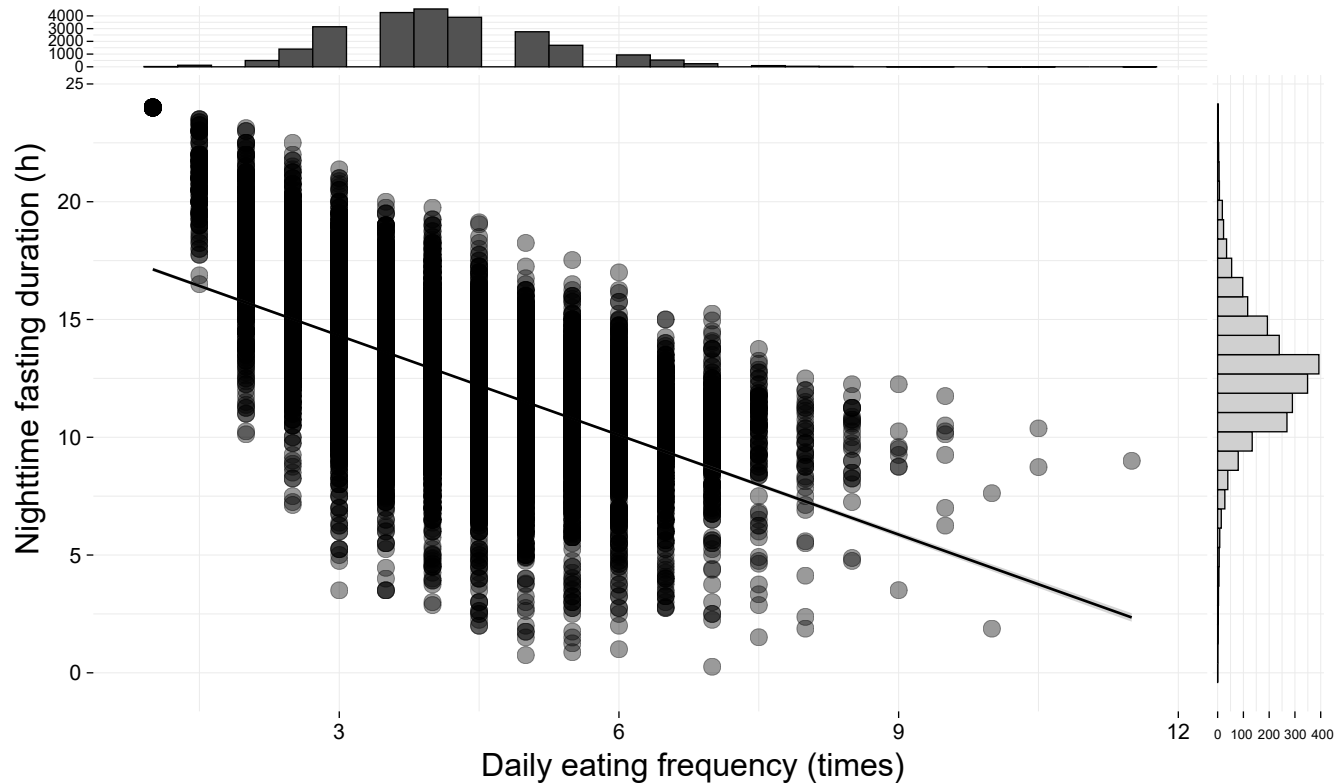

Supplement: Supplementary file 2 — Additional file 2. [file 12966_2024_1654_MOESM2_ESM.pdf]
