# Supplementary material for: Associations of daily eating frequency and nighttime fasting duration with biological aging in National Health and Nutrition Examination Survey (NHANES) 2003–2010 and 2015–2018
Source: Int J Behav Nutr Phys Act. 2024 Sep 19;21:104. doi: 10.1186/s12966-024-01654-y (PMC11414321; doi:10.1186/s12966-024-01654-y)

**Supplementary table 1.** Components of biological age metrics by quintiles of DEF^a^

|  |  | Daily eating frequency (times) | | | | |  |  |
| --- | --- | --- | --- | --- | --- | --- | --- | --- |
|  | Overall | Q1 (≤ 3.0 times) | Q2 (3.1 - 3.5 times) | Q3 (3.6 - 4.0 times) | Q4 (4.1 - 4.5 times) | Q5 (≥ 4.6 times) | *P* | *P*_test_ |
|  |  | N = 5180 | N = 4262 | N = 4544 | N = 3891 | N = 6335 |  |  |
| Albumin, g/L | 42.24 (42.19, 42.28) | 42.01 (41.91, 42.10) | 42.22 (42.11, 42.32) | 42.20 (42.10, 42.30) | 42.27 (42.16, 42.37) | 42.44 (42.36, 42.53) | < 0.001 | 0.017 |
| Alkaline phosphatase, U/L | 71.84 (71.53, 72.15) | 73.28 (72.61, 73.95) | 72.46 (71.71, 73.22) | 71.65 (70.95, 72.35) | 71.53 (70.80, 72.27) | 70.58 (69.98, 71.18) | < 0.001 | < 0.001 |
| C-reactive protein, mg/dL | 0.41 (0.41, 0.43) | 0.49 (0.47, 0.52) | 0.42 (0.40, 0.44) | 0.44 (0.41, 0.47) | 0.40 (0.38, 0.42) | 0.37 (0.36, 0.39) | < 0.001 | < 0.001 |
| Total cholesterol, mg/dL | 193.11 (192.59, 193.63) | 192.12 (190.98, 193.26) | 192.33 (191.06, 193.59) | 192.85 (191.65, 194.04) | 193.84 (192.53, 195.15) | 194.18 (193.18, 195.18) | 0.040 | 0.566 |
| Creatinine, umol/L | 80.26 (79.79, 80.73) | 81.75 (80.61, 82.89) | 81.08 (79.87, 82.29) | 80.95 (79.85, 82.06) | 79.75 (78.52, 80.98) | 78.32 (77.66, 78.97) | < 0.001 | 0.002 |
| Glycated hemoglobin, % | 5.71 (5.70, 5.72) | 5.74 (5.71, 5.77) | 5.78 (5.74, 5.81) | 5.71 (5.68, 5.74) | 5.69 (5.66, 5.72) | 5.66 (5.63, 5.68) | < 0.001 | < 0.001 |
| Systolic blood pressure, mmHg | 124.71 (124.47, 124.95) | 125.14 (124.59, 125.69) | 125.21 (124.62, 125.81) | 124.89 (124.33, 125.45) | 124.09 (123.51, 124.67) | 123.14 (122.70, 123.58) | < 0.001 | < 0.001 |
| Blood urea nitrogen, mmol/L | 4.90 (4.88, 4.93) | 4.92 (4.85, 4.98) | 4.94 (4.88, 5.01) | 4.99 (4.92, 5.05) | 4.91 (4.84, 4.97) | 4.81 (4.76, 4.86) | 0.001 | 0.311 |
| Uric acid, mg/dL | 5.45 (5.43, 5.47) | 5.58 (5.54, 5.62) | 5.48 (5.44, 5.52) | 5.47 (5.43, 5.51) | 5.39 (5.34, 5.43) | 5.34 (5.30, 5.37) | < 0.001 | < 0.001 |
| Lymphocyte percent, % | 30.73 (30.62, 30.84) | 30.88 (30.63, 31.12) | 30.67 (30.41, 30.93) | 30.68 (30.43, 30.93) | 30.61 (30.35, 30.88) | 30.77 (30.57, 30.98) | 0.600 | 0.751 |
| Mean cell volume, fL | 89.19 (89.12, 89.26) | 88.85 (88.69, 89.02) | 89.09 (88.91, 89.26) | 89.33 (89.16, 89.49) | 89.24 (89.05, 89.42) | 89.41 (89.28, 89.55) | < 0.001 | 0.025 |
| White blood cell count, 10^9 cells/L | 7.23 (7.21, 7.26) | 7.32 (7.25, 7.38) | 7.26 (7.19, 7.32) | 7.21 (7.14, 7.28) | 7.23 (7.16, 7.30) | 7.17 (7.11, 7.23) | 0.014 | < 0.001 |

^a^All variables were listed as mean (95% CI). One-way ANOVA was conducted to compare components of biological age metrics. And *P*_test_ was the result of Bonfreni correction.

**Supplementary table 2.** Components of biological age metrics by quintiles of NFD^a^

|  |  | Nighttime fasting duration (hours) | | | | |  |  |
| --- | --- | --- | --- | --- | --- | --- | --- | --- |
|  | Overall | Q1 (≤ 10.0 h) | Q2 (10.1 - 12.0 h) | Q3 (12.1 - 13.0 h) | Q4 (13.1 - 14.0 h) | Q5 (≥ 14.1 h) | *P* | *P*_test_ |
|  |  | N = 3119 | N = 6805 | N = 4325 | N = 2931 | N = 7032 |  |  |
| Albumin, g/L | 42.24 (42.19, 42.28) | 42.45 (42.33, 42.57) | 42.35 (42.27, 42.43) | 42.29 (42.19, 42.39) | 42.12 (41.99, 42.24) | 42.04 (41.96, 42.12) | < 0.001 | 0.001 |
| Alkaline phosphatase, U/L | 71.84 (71.53, 72.15) | 71.48 (70.60, 72.36) | 71.13 (70.57, 71.69) | 71.07 (70.37, 71.76) | 72.79 (71.82, 73.75) | 72.78 (72.20, 73.35) | < 0.001 | 0.001 |
| C-reactive protein, mg/dL | 0.41 (0.41, 0.43) | 0.39 (0.36, 0.42) | 0.39 (0.37, 0.40) | 0.41 (0.39, 0.44) | 0.44 (0.41, 0.47) | 0.48 (0.45, 0.50) | < 0.001 | < 0.001 |
| Total cholesterol, mg/dL | 193.11 (192.59, 193.63) | 191.49 (190.04, 192.93) | 194.20 (193.22, 195.18) | 194.17 (192.96, 195.38) | 192.20 (190.67, 193.72) | 192.50 (191.52, 193.48) | 0.004 | 0.087 |
| Creatinine, umol/L | 80.26 (79.79, 80.73) | 81.28 (80.22, 82.35) | 80.60 (79.67, 81.52) | 80.28 (79.33, 81.24) | 79.75 (78.29, 81.21) | 79.69 (78.78, 80.59) | 0.267 | 0.003 |
| Glycated hemoglobin, % | 5.71 (5.70, 5.72) | 5.66 (5.63, 5.70) | 5.71 (5.69, 5.74) | 5.72 (5.69, 5.75) | 5.75 (5.71, 5.79) | 5.71 (5.69, 5.74) | 0.026 | 0.744 |
| Systolic blood pressure, mmHg | 124.71 (124.47, 124.95) | 123.32 (122.72, 123.93) | 124.22 (123.78, 124.66) | 125.15 (124.56, 125.73) | 124.72 (124.01, 125.42) | 124.50 (124.04, 124.97) | 0.001 | 0.493 |
| Blood urea nitrogen, mmol/L | 4.90 (4.88, 4.93) | 4.82 (4.75, 4.89) | 4.97 (4.92, 5.01) | 4.98 (4.92, 5.05) | 4.94 (4.86, 5.02) | 4.82 (4.77, 4.88) | < 0.001 | 0.004 |
| Uric acid, mg/dL | 5.45 (5.43, 5.47) | 5.47 (5.42, 5.52) | 5.42 (5.39, 5.45) | 5.44 (5.40, 5.48) | 5.44 (5.38, 5.49) | 5.47 (5.44, 5.51) | 0.216 | 0.003 |
| Lymphocyte percent, % | 30.73 (30.62, 30.84) | 30.60 (30.30, 30.91) | 30.64 (30.43, 30.84) | 30.66 (30.42, 30.91) | 30.65 (30.34, 30.96) | 30.96 (30.76, 31.17) | 0.124 | 0.274 |
| Mean cell volume, fL | 89.19 (89.12, 89.26) | 89.48 (89.28, 89.67) | 89.26 (89.13, 89.40) | 89.31 (89.14, 89.48) | 89.28 (89.07, 89.49) | 88.88 (88.74, 89.03) | < 0.001 | 0.010 |
| White blood cell count, 10^9 cells/L | 7.23 (7.21, 7.26) | 7.31 (7.22, 7.39) | 7.22 (7.16, 7.28) | 7.14 (7.07, 7.20) | 7.25 (7.17, 7.33) | 7.27 (7.22, 7.33) | 0.010 | 0.005 |

^a^All variables were listed as mean (95% CI). One-way ANOVA was conducted to compare components of biological age metrics. And *P*_test_ was the result of Bonfreni correction.

**Supplementary table 3.** Differences in the baseline characteristics of participants categorized by quintiles of NFD (n = 24212)^a^

|  |  | Nighttime fasting duration (hours) | | | | |  |  |
| --- | --- | --- | --- | --- | --- | --- | --- | --- |
|  | Overall | Q1 (≤ 10.0 h) | Q2 (10.1 - 12.0 h) | Q3 (12.1 - 13.0 h) | Q4 (13.1 - 14.0 h) | Q5 (≥ 14.1 h) | *P* | *P*_test_ |
|  |  | N = 3119 | N = 6805 | N = 4325 | N = 2931 | N = 7032 |  |  |
| Age, years | 49.09 (48.85, 49.33) | 47.13 (46.51, 47.76) | 50.54 (50.11, 50.97) | 50.71 (50.14, 51.28) | 49.88 (49.18, 50.58) | 47.22 (46.76, 47.69) | < 0.001 | < 0.001 |
| Male, n (%) | 11751 (48.5) | 1891 (60.6) | 3449 (50.7) | 2051 (47.4) | 1291 (44.0) | 3069 (43.6) | < 0.001 | < 0.001 |
| Non-Hispanic white, n (%) | 11171 (46.1) | 1597 (51.2) | 3421 (50.3) | 2054 (47.5) | 1343 (45.8) | 2756 (39.2) | < 0.001 | 0.023 |
| Body mass index, kg/m^2^ | 29.02 (28.93, 29.10) | 28.47 (28.24, 28.69) | 28.73 (28.58, 28.88) | 28.91 (28.71, 29.11) | 29.24 (28.99, 29.49) | 29.51 (29.35, 29.68) | < 0.001 | < 0.001 |
| Current smoking, n (%) | 10504 (43.4) | 1555 (49.9) | 3000 (44.1) | 1794 (41.5) | 1208 (41.2) | 2947 (41.9) | < 0.001 | < 0.001 |
| Current drinking, n (%) | 16329 (67.4) | 2260 (72.5) | 4764 (70.0) | 2887 (66.8) | 1974 (67.3) | 4444 (63.2) | < 0.001 | < 0.001 |
| Regular exercise, n (%) | 8245 (34.1) | 1255 (40.2) | 2429 (35.7) | 1492 (34.5) | 935 (31.9) | 2134 (30.3) | < 0.001 | < 0.001 |
| Above high school, n (%) | 12116 (50.0) | 1742 (55.9) | 3781 (55.6) | 2199 (50.8) | 1479 (50.5) | 2915 (41.5) | < 0.001 | < 0.001 |
| > 55,000 annual household income, n (%) | 8367 (34.6) | 1171 (37.5) | 2633 (38.7) | 1510 (34.9) | 996 (48.1) | 2057 (29.3) | < 0.001 | < 0.001 |
| Sleep duration, hours | 7.29 (7.26, 7.32) | 6.94 (6.84, 7.03) | 7.21 (7.16, 7.25) | 7.41 (7.32, 7.51) | 7.41 (7.31, 7.51) | 7.38 (7.32, 7.45) | < 0.001 | < 0.001 |
| Night shift work, n (%) | 711 (2.9) | 202 (6.5) | 135 (2.0) | 105 (2.4) | 57 (1.9) | 212 (3.0) | < 0.001 | < 0.001 |
| Daily energy intake, kcal/d | 1992.76 (1983.76, 2001.76) | 2360.50 (2335.05, 2385.96) | 2140.15 (2123.56, 2156.73) | 2008.59 (1988.48, 2028.70) | 1911.25 (1887.13, 1935.36) | 1711.28 (1695.99, 1726.56) | < 0.001 | < 0.001 |
| Healthy eating index-2015 | 31.11 (31.00, 31.22) | 31.40 (31.10, 31.71) | 31.61 (31.40, 31.82) | 31.39 (31.13, 31.64) | 31.19 (30.88, 31.50) | 30.30 (30.10, 30.50) | < 0.001 | < 0.001 |
| Dietary supplements use, n (%) | 12226 (50.5) | 1581 (50.7) | 3798 (55.8) | 2254 (52.1) | 1524 (52.0) | 3069 (43.6) | < 0.001 | < 0.001 |
| Dietary data surveyed on weekend, n (%) | 1147 (4.7) | 141 (4.5) | 278 (4.1) | 209 (4.8) | 153 (5.2) | 366 (5.2) | 0.019 | 0.402 |
| Self-reported cancer, n (%) | 2315 (9.6) | 277 (8.9) | 700 (10.3) | 407 (9.4) | 315 (10.7) | 616 (8.8) | < 0.001 | < 0.001 |
| Self-reported hypertension, n (%) | 8424 (34.8) | 1011 (32.4) | 2337 (34.3) | 1586 (36.7) | 1045 (35.7) | 2445 (34.8) | 0.001 | 0.475 |
| Self-reported cardiovascular diseases, n (%) | 2581 (10.7) | 292 (9.4) | 700 (10.3) | 460 (10.4) | 334 (11.4) | 795 (11.3) | < 0.001 | < 0.001 |
| Self-reported diabetes, n (%) | 2956 (12.2) | 312 (10.0) | 799 (11.7) | 540 (12.5) | 413 (14.1) | 892 (12.7) | < 0.001 | 0.529 |
| Daily eating frequency, times | 4.13 (4.12, 4.14) | 5.03 (4.99, 5.07) | 4.60 (4.58, 4.63) | 4.21 (4.19, 4.24) | 3.96 (3.93, 3.99) | 3.30 (3.28, 3.32) | < 0.001 | < 0.001 |
| Nighttime fasting duration, hours | 12.72 (12.69, 12.76) | 8.58 (8.53, 8.64) | 11.23 (11.21, 11.24) | 12.61 (12.60, 12.62) | 13.48 (13.48, 13.49) | 15.76 (15.72, 15.80) | < 0.001 | < 0.001 |
| Lactate dehydrogenase, U/L | 135.23 (134.80, 135.66) | 136.72 (135.24, 138.20) | 136.02 (135.22, 136.82) | 134.97 (134.00, 135.94) | 133.64 (132.58, 134.70) | 134.63 (133.82, 135.44) | 0.001 | 0.021 |
| Globulin, g/dL | 2.96 (2.95, 2.96) | 2.91 (2.89, 2.92) | 2.93 (2.92, 2.94) | 2.95 (2.94, 2.97) | 2.97 (2.95, 2.99) | 3.01 (3.00, 3.02) | < 0.001 | < 0.001 |
| Homeostatic dysregulation | 1.68 (1.68, 1.69) | 1.64 (1.61, 1.66) | 1.66 (1.64, 1.67) | 1.68 (1.66, 1.71) | 1.71 (1.68, 1.74) | 1.73 (1.71, 1.75) | < 0.001 | < 0.001 |
| Klemera-Doubal method, years | 41.98 (41.76, 42.20) | 40.01 (39.43, 40.59) | 42.62 (42.22, 43.03) | 43.20 (42.66, 43.74) | 42.73 (42.08, 43.38) | 41.17 (40.73, 41.61) | < 0.001 | < 0.001 |
| Klemera-Doubal method residual, years | 0.00 (-0.10, 0.10) | 0.06 (-0.21, 0.33) | -0.84 (-1.03, -0.65) | -0.45 (-0.69, -0.21) | -0.08 (-0.37, 0.22) | 1.08 (0.89, 1.28) | < 0.001 | < 0.001 |
| PhenoAge, years | 48.33 (48.07, 48.59) | 46.65 (45.99, 47.32) | 49.55 (49.09, 50.01) | 49.75 (49.14, 50.36) | 49.16 (48.40, 49.91) | 46.67 (46.17, 47.17) | < 0.001 | < 0.001 |
| PhenoAge residual, years | 0.01 (-0.05, 0.07) | 0.46 (0.29, 0.62) | -0.34 (-0.45, -0.23) | -0.33 (-0.47, -0.19) | -0.04 (-0.21, 0.14) | 0.38 (0.27, 0.50) | < 0.001 | < 0.001 |
| Allostatic load | 0.28 (0.28, 0.29) | 0.28 (0.27, 0.28) | 0.28 (0.28, 0.28) | 0.28 (0.28, 0.29) | 0.29 (0.28, 0.29) | 0.29 (0.28, 0.29) | 0.031 | 0.119 |

^a^Continuous variables were presented as mean (95% CI). Categorical variables were listed as N (%). And Ptest was the result of Bonfreni correction.

**Supplementary table 4.** Associations of DEF with predicted age metrics

|  |  | Daily eating frequency (times) | | | | | | | | |  |  |
| --- | --- | --- | --- | --- | --- | --- | --- | --- | --- | --- | --- | --- |
|  |  | Q1^c^ | Q2 | | Q3 | | Q4 | | Q5 | | *P*_trend_^e^ | *P*_test_^f^ |
|  |  | β^b^ | β | 95% CI | β | 95% CI | β | 95% CI | β | 95% CI |  |  |
| Ln-transformed HD |  |  |  |  |  |  |  |  |  |  |  |  |
|  | Model 1^a^ | Ref (0.00)^d^ | -0.02 | (-0.04, 0.00) | -0.04 | **(-0.06, -0.02)***** | -0.06 | **(-0.08, -0.04)***** | -0.08 | **(-0.09, -0.06)***** | < 0.001 | < 0.001 |
|  | Model 2 | Ref (0.00) | 0.00 | (-0.02, 0.02) | -0.02 | (-0.04, 0.00) | -0.03 | **(-0.05, -0.01)**** | -0.04 | **(-0.05, -0.02)***** | < 0.001 | < 0.001 |
|  | Model 3 | Ref (0.00) | 0.01 | (-0.01, 0.02) | -0.01 | (-0.03, 0.01) | -0.02 | (-0.04, 0.00) | -0.02 | **(-0.04, -0.01)*** | < 0.001 | < 0.001 |
|  | Model 4 | Ref (0.00) | 0.01 | (-0.01, 0.03) | -0.01 | (-0.03, 0.01) | -0.02 | (-0.04, 0.00) | -0.02 | (-0.04, 0.00) | 0.002 | < 0.001 |
| KDM residual (years) |  |  |  |  |  |  |  |  |  |  |  |  |
|  | Model 1 | Ref (0.00) | -0.64 | **(-1.06, -0.21)**** | -0.90 | **(-1.29, -0.52)***** | -1.17 | **(-1.58, -0.75)***** | -1.74 | **(-2.08, -1.40)***** | < 0.001 | < 0.001 |
|  | Model 2 | Ref (0.00) | -0.25 | (-0.62, 0.11) | -0.44 | **(-0.78, -0.11)**** | -0.56 | **(-0.94, -0.19)**** | -0.89 | **(-1.20, -0.59)***** | < 0.001 | < 0.001 |
|  | Model 3 | Ref (0.00) | -0.12 | (-0.47, 0.24) | -0.31 | (-0.65, 0.03) | -0.36 | (-0.73, 0.01) | -0.59 | **(-0.92, -0.27)***** | < 0.001 | < 0.001 |
|  | Model 4 | Ref (0.00) | -0.11 | (-0.48, 0.27) | -0.30 | (-0.65, 0.06) | -0.34 | (-0.75, 0.06) | -0.57 | **(-0.97, -0.17)**** | 0.003 | 0.006 |
| PA residual (years) |  |  |  |  |  |  |  |  |  |  |  |  |
|  | Model 1 | Ref (0.00) | -0.41 | **(-0.70, -0.13)**** | -0.61 | **(-0.84, -0.38)***** | -0.82 | **(-1.07, -0.57)***** | -1.11 | **(-1.31, -0.90)***** | < 0.001 | < 0.001 |
|  | Model 2 | Ref (0.00) | -0.14 | (-0.38, 0.11) | -0.28 | **(-0.48, -0.08)**** | -0.39 | **(-0.60, -0.17)***** | -0.50 | **(-0.68, -0.31)***** | < 0.001 | < 0.001 |
|  | Model 3 | Ref (0.00) | -0.05 | (-0.30, 0.19) | -0.21 | (-0.41, 0.00) | -0.29 | **(-0.50, -0.07)**** | -0.33 | **(-0.54, -0.13)***** | < 0.001 | 0.001 |
|  | Model 4 | Ref (0.00) | -0.12 | (-0.37, 0.14) | -0.30 | **(-0.50, -0.09)**** | -0.39 | **(-0.62, -0.16)***** | -0.47 | **(-0.69, -0.25)***** | < 0.001 | < 0.001 |
| AL |  |  |  |  |  |  |  |  |  |  |  |  |
|  | Model 1 | Ref (0.00) | -0.01 | (-0.02, 0.00) | -0.02 | **(-0.03, -0.01)***** | -0.02 | **(-0.03, -0.02)***** | -0.03 | **(-0.04, -0.03)***** | < 0.001 | < 0.001 |
|  | Model 2 | Ref (0.00) | 0.00 | (-0.01, 0.00) | -0.01 | (-0.02, 0.00) | -0.01 | (-0.02, 0.00) | -0.02 | **(-0.02, -0.01)***** | < 0.001 | < 0.001 |
|  | Model 3 | Ref (0.00) | 0.00 | (-0.01, 0.01) | -0.01 | (-0.02, 0.00) | -0.01 | (-0.02, 0.00) | -0.01 | (-0.02, 0.00) | 0.002 | 0.003 |
|  | Model 4 | Ref (0.00) | 0.00 | (-0.01, 0.01) | -0.01 | (-0.02, 0.00) | -0.01 | (-0.02, 0.00) | -0.01 | (-0.02, 0.00) | 0.004 | 0.005 |

^a^Model 1 was adjusted for age, sex, race, and NHANES cycle. Model 2 further adjusted for BMI, smoking, drinking, exercise, education, income, sleep duration, and shift work. Model 3 further adjusted for daily energy intake, nutrient supplement use, HEI-2015, dietary data surveyed on weekend, self-reported cancer, CVD, hypertension, and diabetes. Model 4 further adjusted for NFD.

^b^Data were listed as the weighted beta estimates and 95% confidence intervals, with *p < 0.05, **p < 0.01, ***p < 0.001.

^c^Q, quintile.

^d^Ref, reference.

^e^Tests for trends based on the variables containing the median values for each quartile.

^f^*P*_test_ was the result of Bonfreni correction.

**Supplementary table 5.** Associations of NFD with predicted age metrics

|  |  | Nighttime fasting duration (hours) | | | | | | | | |  |  |
| --- | --- | --- | --- | --- | --- | --- | --- | --- | --- | --- | --- | --- |
|  |  | Q1^c^ | | Q2 | Q3 | | Q4 | | Q5 | | *P*_trend_^e^ | *P*_test_^f^ |
|  |  | β^b^ | 95% CI | β | β | 95% CI | β | 95% CI | β | 95% CI |  |  |
| Ln-transformed HD |  |  |  |  |  |  |  |  |  |  |  |  |
|  | Model 1^a^ | 0.02 | (0.00, 0.04) | Ref (0.00)^d^ | 0.02 | (0.00, 0.03) | 0.03 | **(0.01, 0.05)**** | 0.07 | **(0.05, 0.08)***** | < 0.001 | < 0.001 |
|  | Model 2 | 0.02 | **(0.01, 0.04)**** | Ref (0.00) | 0.01 | (0.00, 0.02) | 0.02 | (0.00, 0.04) | 0.04 | **(0.03, 0.06)***** | < 0.001 | < 0.001 |
|  | Model 3 | 0.02 | **(0.01, 0.04)**** | Ref (0.00) | 0.01 | (-0.01, 0.02) | 0.01 | (0.00, 0.03) | 0.03 | **(0.02, 0.04)***** | 0.006 | < 0.001 |
|  | Model 4 | 0.03 | **(0.01, 0.04)**** | Ref (0.00) | 0.01 | (-0.01, 0.02) | 0.01 | (-0.01, 0.03) | 0.02 | **(0.01, 0.04)**** | 0.205 | < 0.001 |
| KDM residual (years) |  |  |  |  |  |  |  |  |  |  |  |  |
|  | Model 1 | 0.24 | (-0.11, 0.58) | Ref (0.00) | 0.28 | (-0.08, 0.63) | 0.55 | **(0.15, 0.94)**** | 1.27 | **(0.97, 1.57)***** | < 0.001 | < 0.001 |
|  | Model 2 | 0.33 | **(0.04, 0.62)*** | Ref (0.00) | 0.17 | (-0.16, 0.50) | 0.31 | (-0.07, 0.69) | 0.78 | **(0.51, 1.06)***** | < 0.001 | < 0.001 |
|  | Model 3 | 0.27 | (0.00, 0.55) | Ref (0.00) | 0.10 | (-0.22, 0.42) | 0.22 | (-0.15, 0.58) | 0.53 | **(0.26, 0.80)***** | 0.009 | < 0.001 |
|  | Model 4 | 0.34 | **(0.05, 0.63)*** | Ref (0.00) | 0.04 | (-0.28, 0.36) | 0.11 | (-0.27, 0.49) | 0.33 | **(0.03, 0.62)*** | 0.575 | 0.030 |
| PA residual (years) |  |  |  |  |  |  |  |  |  |  |  |  |
|  | Model 1 | 0.31 | **(0.08, 0.55)**** | Ref (0.00) | 0.04 | (-0.16, 0.25) | 0.33 | **(0.09, 0.56)**** | 0.75 | **(0.56, 0.94)***** | < 0.001 | < 0.001 |
|  | Model 2 | 0.37 | **(0.18, 0.57)***** | Ref (0.00) | -0.03 | (-0.21, 0.15) | 0.16 | (-0.05, 0.37) | 0.40 | **(0.24, 0.56)***** | 0.066 | < 0.001 |
|  | Model 3 | 0.33 | **(0.13, 0.52)***** | Ref (0.00) | -0.06 | (-0.23, 0.12) | 0.12 | (-0.09, 0.32) | 0.25 | **(0.09, 0.41)**** | 0.655 | 0.003 |
|  | Model 4 | 0.38 | **(0.18, 0.57)***** | Ref (0.00) | -0.10 | (-0.27, 0.07) | 0.04 | (-0.16, 0.25) | 0.11 | (-0.06, 0.27) | 0.094 | 0.200 |
| AL |  |  |  |  |  |  |  |  |  |  |  |  |
|  | Model 1 | 0.01 | (0.00, 0.02) | Ref (0.00) | 0.01 | (0.00, 0.01) | 0.01 | (0.00, 0.02) | 0.03 | **(0.02, 0.03)***** | < 0.001 | < 0.001 |
|  | Model 2 | 0.01 | **(0.01, 0.02)***** | Ref (0.00) | 0.00 | (0.00, 0.01) | 0.00 | (-0.01, 0.01) | 0.02 | **(0.01, 0.02)***** | 0.006 | < 0.001 |
|  | Model 3 | 0.01 | (0.00, 0.02) | Ref (0.00) | 0.00 | (0.00, 0.01) | 0.00 | (-0.01, 0.01) | 0.01 | **(0.01, 0.02)***** | 0.069 | < 0.001 |
|  | Model 4 | 0.01 | **(0.01, 0.02)***** | Ref (0.00) | 0.00 | (-0.01, 0.01) | 0.00 | (-0.01, 0.01) | 0.01 | (0.00, 0.01) | 0.642 | 0.027 |

^a^Model 1 was adjusted for age, sex, race, and NHANES cycle. Model 2 further adjusted for BMI, smoking, drinking, exercise, education, income, sleep duration, and shift work. Model 3 further adjusted for daily energy intake, nutrient supplement use, HEI-2015, dietary data surveyed on weekend, self-reported cancer, CVD, hypertension, and diabetes. Model 4 further adjusted for DEF.

^b^Data were listed as the weighted beta estimates and 95% confidence intervals, with *p < 0.05, **p < 0.01, ***p < 0.001.

^c^Q, quintile.

^d^Ref, reference.

^e^Tests for trends based on the variables containing the median values for each quartile.

^f^*P*_test_ was the result of Bonfreni correction.

**Supplementary table 6.** Association of DEF with Ln-transformed HD stratified by variables of interest

|  |  |  | Daily eating frequency (times) | | | | | | | | |  |  |  |
| --- | --- | --- | --- | --- | --- | --- | --- | --- | --- | --- | --- | --- | --- | --- |
|  |  |  | Q1^c^ | Q2 | | Q3 | | Q4 | | Q5 | | *P*_trend_^e^ | *P*_test_^f^ | *P*_interaction_^g^ |
|  |  |  | β^b^ | β | 95% CI | β | 95% CI | β | 95% CI | β | 95% CI |  |  |  |
| Age |  |  |  |  |  |  |  |  |  |  |  |  |  |  |
|  | Model 1^a^ | > 60 years | Ref (0.00)^d^ | -0.02 | (-0.06,0.01) | -0.03 | (-0.07,0.00) | -0.07 | **(-0.11,-0.04)***** | -0.10 | **(-0.13,-0.07)***** | <0.001 | <0.001 | 0.008 |
|  |  | ≤ 60 years | Ref (0.00) | -0.01 | (-0.03,0.02) | -0.03 | (-0.05,0.00) | -0.04 | **(-0.06,-0.01)**** | -0.04 | **(-0.06,-0.02)***** | <0.001 | <0.001 |  |
|  | Model 2 | > 60 years | Ref (0.00) | -0.01 | (-0.04,0.03) | -0.02 | (-0.05,0.02) | -0.06 | **(-0.09,-0.02)***** | -0.07 | **(-0.10,-0.04)***** | <0.001 | <0.001 | 0.001 |
|  |  | ≤ 60 years | Ref (0.00) | 0.01 | (-0.01,0.03) | -0.01 | (-0.03,0.02) | -0.01 | (-0.03,0.01) | -0.01 | (-0.03,0.01) | 0.209 | 0.494 |  |
|  | Model 3 | > 60 years | Ref (0.00) | 0.00 | (-0.03,0.04) | 0.00 | (-0.04,0.03) | -0.03 | (-0.07,0.00) | -0.04 | (-0.07,0.00) | 0.004 | 0.033 | <0.001 |
|  |  | ≤ 60 years | Ref (0.00) | 0.02 | (0.00,0.04) | 0.00 | (-0.02,0.02) | 0.00 | (-0.02,0.02) | 0.01 | (-0.01,0.03) | 0.753 | 0.407 |  |
|  | Model 4 | > 60 years | Ref (0.00) | 0.00 | (-0.03,0.04) | 0.00 | (-0.04,0.04) | -0.03 | (-0.07,0.01) | -0.03 | (-0.07,0.01) | 0.023 | 0.133 | <0.001 |
|  |  | ≤ 60 years | Ref (0.00) | 0.01 | (-0.01,0.03) | 0.00 | (-0.02,0.02) | 0.00 | (-0.03,0.02) | 0.00 | (-0.02,0.02) | 0.609 | 0.991 |  |
| Sex |  |  |  |  |  |  |  |  |  |  |  |  |  |  |
|  | Model 1 | Male | Ref (0.00) | -0.01 | (-0.04,0.01) | -0.02 | (-0.05,0.00) | -0.05 | **(-0.08,-0.02)***** | -0.07 | **(-0.09,-0.04)***** | <0.001 | <0.001 | 0.404 |
|  |  | Female | Ref (0.00) | -0.03 | (-0.05,0.00) | -0.05 | **(-0.08,-0.03)***** | -0.07 | **(-0.09,-0.04)***** | -0.08 | **(-0.10,-0.06)***** | <0.001 | <0.001 |  |
|  | Model 2 | Male | Ref (0.00) | 0.00 | (-0.03,0.02) | -0.01 | (-0.03,0.01) | -0.03 | (-0.06,0.00) | -0.04 | **(-0.06,-0.02)**** | <0.001 | 0.002 | 0.999 |
|  |  | Female | Ref (0.00) | 0.00 | (-0.03,0.02) | -0.03 | (-0.06,0.00) | -0.04 | **(-0.06,-0.01)**** | -0.04 | **(-0.06,-0.02)***** | <0.001 | <0.001 |  |
|  | Model 3 | Male | Ref (0.00) | 0.01 | (-0.01,0.03) | 0.00 | (-0.02,0.03) | -0.01 | (-0.04,0.01) | -0.01 | (-0.03,0.01) | 0.072 | 0.309 | 0.966 |
|  |  | Female | Ref (0.00) | 0.00 | (-0.02,0.03) | -0.02 | (-0.05,0.01) | -0.03 | (-0.06,0.00) | -0.03 | **(-0.05,-0.01)*** | 0.001 | 0.013 |  |
|  | Model 4 | Male | Ref (0.00) | 0.01 | (-0.02,0.03) | 0.00 | (-0.02,0.03) | -0.01 | (-0.04,0.02) | -0.01 | (-0.04,0.01) | 0.051 | 0.284 | 0.966 |
|  |  | Female | Ref (0.00) | 0.00 | (-0.02,0.03) | -0.02 | (-0.05,0.01) | -0.03 | (-0.06,0.00) | -0.03 | (-0.05,0.00) | 0.009 | 0.053 |  |
| Race |  |  |  |  |  |  |  |  |  |  |  |  |  |  |
|  | Model 1 | Non-hispanic white | Ref (0.00) | -0.01 | (-0.04,0.01) | -0.03 | **(-0.06,-0.01)*** | -0.05 | **(-0.07,-0.02)***** | -0.06 | **(-0.08,-0.04)***** | <0.001 | <0.001 | 0.752 |
|  |  | Others | Ref (0.00) | -0.02 | (-0.04,0.01) | -0.03 | **(-0.06,-0.01)***** | -0.07 | **(-0.09,-0.04)***** | -0.08 | **(-0.10,-0.05)***** | <0.001 | <0.001 |  |
|  | Model 2 | Non-hispanic white | Ref (0.00) | 0.00 | (-0.02,0.03) | -0.01 | (-0.04,0.01) | -0.02 | (-0.04,0.00) | -0.03 | **(-0.05,-0.01)**** | 0.001 | 0.007 | 0.841 |
|  |  | Others | Ref (0.00) | -0.01 | (-0.03,0.02) | -0.02 | (-0.04,0.00) | -0.05 | **(-0.07,-0.02)***** | -0.05 | **(-0.07,-0.03)***** | <0.001 | <0.001 |  |
|  | Model 3 | Non-hispanic white | Ref (0.00) | 0.01 | (-0.01,0.03) | 0.00 | (-0.03,0.02) | -0.01 | (-0.03,0.01) | -0.01 | (-0.03,0.01) | 0.150 | 0.466 | 0.916 |
|  |  | Others | Ref (0.00) | 0.00 | (-0.03,0.02) | -0.02 | (-0.04,0.00) | -0.04 | **(-0.07,-0.02)**** | -0.04 | **(-0.07,-0.02)***** | <0.001 | 0.001 |  |
|  | Model 4 | Non-hispanic white | Ref (0.00) | 0.01 | (-0.02,0.04) | 0.00 | (-0.03,0.02) | -0.01 | (-0.03,0.01) | -0.01 | (-0.03,0.02) | 0.164 | 0.480 | 0.920 |
|  |  | Others | Ref (0.00) | 0.00 | (-0.03,0.02) | -0.02 | (-0.04,0.00) | -0.04 | **(-0.07,-0.01)**** | -0.04 | **(-0.07,-0.02)**** | <0.001 | 0.003 |  |
| BMI |  |  |  |  |  |  |  |  |  |  |  |  |  |  |
|  | Model 1 | < 30 kg/m^2^ | Ref (0.00) | -0.02 | (-0.04,0.01) | -0.03 | **(-0.05,-0.01)**** | -0.05 | **(-0.07,-0.03)***** | -0.06 | **(-0.07,-0.04)***** | <0.001 | <0.001 | 0.031 |
|  |  | ≥ 30 kg/m^2^ | Ref (0.00) | 0.00 | (-0.03,0.03) | -0.04 | **(-0.07,-0.01)*** | -0.05 | **(-0.08,-0.02)***** | -0.07 | **(-0.10,-0.05)***** | <0.001 | <0.001 |  |
|  | Model 2 | < 30 kg/m^2^ | Ref (0.00) | -0.01 | (-0.03,0.01) | -0.02 | (-0.04,0.00) | -0.03 | **(-0.05,-0.01)**** | -0.03 | **(-0.05,-0.02)***** | <0.001 | <0.001 | 0.029 |
|  |  | ≥ 30 kg/m^2^ | Ref (0.00) | 0.00 | (-0.03,0.03) | -0.03 | (-0.06,0.00) | -0.04 | **(-0.07,-0.01)**** | -0.06 | **(-0.08,-0.03)***** | <0.001 | <0.001 |  |
|  | Model 3 | < 30 kg/m^2^ | Ref (0.00) | 0.00 | (-0.02,0.03) | -0.01 | (-0.02,0.01) | -0.02 | (-0.04,0.01) | -0.01 | (-0.03,0.01) | 0.118 | 0.280 | 0.018 |
|  |  | ≥ 30 kg/m^2^ | Ref (0.00) | 0.01 | (-0.02,0.04) | -0.03 | (-0.06,0.01) | -0.03 | **(-0.06,-0.01)*** | -0.05 | **(-0.07,-0.02)***** | <0.001 | <0.001 |  |
|  | Model 4 | < 30 kg/m^2^ | Ref (0.00) | 0.00 | (-0.02,0.03) | -0.01 | (-0.03,0.01) | -0.02 | (-0.04,0.01) | -0.01 | (-0.03,0.01) | 0.158 | 0.336 | 0.018 |
|  |  | ≥ 30 kg/m^2^ | Ref (0.00) | 0.01 | (-0.03,0.04) | -0.02 | (-0.06,0.01) | -0.03 | (-0.06,0.00) | -0.05 | **(-0.08,-0.01)**** | <0.001 | 0.008 |  |
| Smoking |  |  |  |  |  |  |  |  |  |  |  |  |  |  |
|  | Model 1 | Yes | Ref (0.00) | -0.02 | (-0.04,0.00) | -0.02 | (-0.04,0.01) | -0.05 | **(-0.08,-0.02)***** | -0.06 | **(-0.08,-0.03)***** | <0.001 | <0.001 | 0.333 |
|  |  | No | Ref (0.00) | -0.02 | (-0.05,0.01) | -0.06 | **(-0.08,-0.03)***** | -0.06 | **(-0.09,-0.04)***** | -0.09 | **(-0.11,-0.07)***** | <0.001 | <0.001 |  |
|  | Model 2 | Yes | Ref (0.00) | -0.01 | (-0.03,0.01) | 0.00 | (-0.03,0.03) | -0.03 | (-0.06,0.00) | -0.03 | **(-0.05,-0.01)*** | 0.007 | 0.017 | 0.602 |
|  |  | No | Ref (0.00) | 0.00 | (-0.02,0.03) | -0.03 | **(-0.05,-0.01)**** | -0.03 | **(-0.06,-0.01)**** | -0.05 | **(-0.07,-0.03)***** | <0.001 | <0.001 |  |
|  | Model 3 | Yes | Ref (0.00) | 0.00 | (-0.02,0.02) | 0.01 | (-0.02,0.03) | -0.02 | (-0.05,0.01) | -0.01 | (-0.03,0.01) | 0.236 | 0.412 | 0.625 |
|  |  | No | Ref (0.00) | 0.01 | (-0.02,0.04) | -0.02 | (-0.04,0.00) | -0.02 | (-0.04,0.00) | -0.03 | **(-0.05,-0.01)**** | <0.001 | 0.010 |  |
|  | Model 4 | Yes | Ref (0.00) | 0.00 | (-0.02,0.02) | 0.01 | (-0.02,0.04) | -0.02 | (-0.04,0.01) | -0.01 | (-0.03,0.02) | 0.316 | 0.557 | 0.627 |
|  |  | No | Ref (0.00) | 0.01 | (-0.02,0.04) | -0.02 | (-0.04,0.00) | -0.02 | (-0.05,0.01) | -0.03 | **(-0.05,-0.01)*** | 0.001 | 0.019 |  |
| Drinking |  |  |  |  |  |  |  |  |  |  |  |  |  |  |
|  | Model 1 | Yes | Ref (0.00) | -0.02 | (-0.04,0.01) | -0.03 | **(-0.06,-0.01)**** | -0.06 | **(-0.08,-0.04)***** | -0.07 | **(-0.09,-0.05)***** | <0.001 | <0.001 | 0.848 |
|  |  | No | Ref (0.00) | -0.02 | (-0.06,0.02) | -0.04 | (-0.07,0.00) | -0.06 | **(-0.10,-0.03)***** | -0.08 | **(-0.11,-0.05)***** | <0.001 | <0.001 |  |
|  | Model 2 | Yes | Ref (0.00) | 0.00 | (-0.02,0.02) | -0.01 | (-0.03,0.01) | -0.03 | **(-0.05,-0.01)***** | -0.04 | **(-0.05,-0.02)***** | <0.001 | <0.001 | 0.810 |
|  |  | No | Ref (0.00) | 0.00 | (-0.04,0.04) | -0.02 | (-0.05,0.02) | -0.04 | (-0.07,0.00) | -0.04 | **(-0.07,-0.01)**** | <0.001 | 0.007 |  |
|  | Model 3 | Yes | Ref (0.00) | 0.01 | (-0.01,0.03) | -0.01 | (-0.03,0.02) | -0.02 | (-0.04,0.00) | -0.02 | (-0.03,0.00) | 0.007 | 0.089 | 0.878 |
|  |  | No | Ref (0.00) | 0.00 | (-0.04,0.04) | -0.01 | (-0.05,0.02) | -0.03 | (-0.07,0.01) | -0.03 | (-0.07,0.00) | 0.018 | 0.079 |  |
|  | Model 4 | Yes | Ref (0.00) | 0.01 | (-0.01,0.04) | 0.00 | (-0.03,0.02) | -0.02 | (-0.04,0.01) | -0.01 | (-0.03,0.01) | 0.041 | 0.291 | 0.875 |
|  |  | No | Ref (0.00) | 0.00 | **(0.04,0.90)***** | -0.02 | **(0.02,0.40)***** | -0.04 | **(0.00,0.08)***** | -0.04 | **(0.00,0.06)***** | 0.011 | 0.057 |  |
| Exercise |  |  |  |  |  |  |  |  |  |  |  |  |  |  |
|  | Model 1 | Yes | Ref (0.00) | -0.02 | (-0.05,0.01) | -0.04 | **(-0.06,-0.02)**** | -0.05 | **(-0.08,-0.03)***** | -0.08 | **(-0.10,-0.05)***** | <0.001 | <0.001 | 0.664 |
|  |  | No | Ref (0.00) | -0.01 | (-0.04,0.01) | -0.03 | **(-0.06,-0.01)**** | -0.06 | **(-0.08,-0.03)***** | -0.07 | **(-0.09,-0.04)***** | <0.001 | <0.001 |  |
|  | Model 2 | Yes | Ref (0.00) | -0.01 | (-0.04,0.02) | -0.02 | (-0.04,0.00) | -0.03 | **(-0.06,-0.01)*** | -0.05 | **(-0.07,-0.02)***** | <0.001 | <0.001 | 0.802 |
|  |  | No | Ref (0.00) | 0.00 | (-0.02,0.02) | -0.02 | (-0.04,0.01) | -0.03 | **(-0.06,-0.01)**** | -0.03 | **(-0.05,-0.02)**** | <0.001 | <0.001 |  |
|  | Model 3 | Yes | Ref (0.00) | -0.01 | (-0.04,0.02) | -0.02 | (-0.04,0.01) | -0.02 | (-0.05,0.00) | -0.04 | **(-0.06,-0.01)**** | 0.002 | 0.004 | 0.823 |
|  |  | No | Ref (0.00) | 0.01 | (-0.01,0.04) | -0.01 | (-0.03,0.02) | -0.02 | (-0.04,0.00) | -0.01 | (-0.03,0.01) | 0.064 | <0.001 |  |
|  | Model 4 | Yes | Ref (0.00) | -0.01 | (-0.04,0.02) | -0.02 | (-0.04,0.01) | -0.03 | (-0.05,0.01) | -0.04 | **(-0.06,-0.01)*** | 0.003 | 0.011 | 0.821 |
|  |  | No | Ref (0.00) | 0.01 | (-0.01,0.04) | -0.01 | (-0.03,0.02) | -0.02 | (-0.04,0.01) | -0.01 | (-0.04,0.01) | 0.099 | <0.001 |  |
| Education |  |  |  |  |  |  |  |  |  |  |  |  |  |  |
|  | Model 1 | Above high school | Ref (0.00) | -0.02 | (-0.05,0.01) | -0.04 | **(-0.06,-0.01)**** | -0.06 | **(-0.08,-0.04)***** | -0.08 | **(-0.10,-0.06)***** | <0.001 | <0.001 | 0.205 |
|  |  | Others | Ref (0.00) | 0.00 | (-0.03,0.02) | -0.03 | **(-0.05,-0.01)*** | -0.04 | **(-0.06,-0.01)**** | -0.04 | **(-0.07,-0.02)***** | <0.001 | 0.001 |  |
|  | Model 2 | Above high school | Ref (0.00) | -0.01 | (-0.04,0.02) | -0.02 | (-0.05,0.00) | -0.04 | **(-0.07,-0.02)***** | -0.05 | **(-0.07,-0.03)***** | <0.001 | <0.001 | 0.211 |
|  |  | Others | Ref (0.00) | 0.00 | (-0.02,0.03) | -0.02 | (-0.04,0.01) | -0.03 | (-0.05,0.00) | -0.02 | (-0.05,0.00) | 0.022 | 0.072 |  |
|  | Model 3 | Above high school | Ref (0.00) | 0.00 | (-0.03,0.02) | -0.02 | (-0.04,0.01) | -0.03 | **(-0.06,-0.01)*** | -0.04 | **(-0.06,-0.02)***** | <0.001 | <0.001 | 0.194 |
|  |  | Others | Ref (0.00) | 0.01 | (-0.01,0.04) | -0.01 | (-0.03,0.02) | -0.01 | (-0.03,0.02) | 0.00 | (-0.02,0.03) | 0.933 | 0.759 |  |
|  | Model 4 | Above high school | Ref (0.00) | -0.01 | (-0.03,0.02) | -0.02 | (-0.04,0.00) | -0.04 | **(-0.06,-0.01)**** | -0.04 | **(-0.06,-0.02)***** | <0.001 | <0.001 | 0.192 |
|  |  | Others | Ref (0.00) | 0.02 | (-0.01,0.04) | 0.00 | (-0.03,0.03) | 0.00 | (-0.03,0.03) | 0.01 | (-0.02,0.04) | 0.689 | 0.442 |  |
| Income |  |  |  |  |  |  |  |  |  |  |  |  |  |  |
|  | Model 1 | ≤ $55,000 | Ref (0.00) | -0.02 | (-0.05,0.00) | -0.05 | **(-0.07,-0.02)***** | -0.05 | **(-0.08,-0.03)***** | -0.07 | **(-0.09,-0.05)***** | <0.001 | <0.001 | 0.860 |
|  |  | > $55,000 | Ref (0.00) | -0.01 | (-0.04,0.03) | -0.02 | (-0.05,0.00) | -0.06 | **(-0.08,-0.03)***** | -0.06 | **(-0.08,-0.04)***** | <0.001 | <0.001 |  |
|  | Model 2 | ≤ $55,000 | Ref (0.00) | -0.01 | (-0.03,0.02) | -0.03 | (-0.05,0.00) | -0.03 | **(-0.05,-0.01)*** | -0.04 | **(-0.05,-0.02)***** | <0.001 | <0.001 | 0.748 |
|  |  | > $55,000 | Ref (0.00) | 0.01 | (-0.03,0.03) | -0.01 | (-0.04,0.02) | -0.04 | **(-0.07,-0.01)**** | -0.04 | **(-0.06,-0.01)**** | <0.001 | 0.003 |  |
|  | Model 3 | ≤ $55,000 | Ref (0.00) | 0.00 | (-0.02,0.02) | -0.02 | (-0.04,0.01) | -0.02 | (-0.04,0.01) | -0.02 | (-0.04,0.00) | 0.032 | 0.090 | 0.627 |
|  |  | > $55,000 | Ref (0.00) | 0.01 | (-0.02,0.04) | 0.00 | (-0.03,0.03) | -0.03 | (-0.06,0.00) | -0.02 | (-0.04,0.01) | 0.016 | 0.155 |  |
|  | Model 4 | ≤ $55,000 | Ref (0.00) | 0.00 | (-0.02,0.03) | -0.02 | (-0.04,0.01) | -0.01 | (-0.04,0.01) | -0.01 | (-0.04,0.01) | 0.096 | 0.194 | 0.623 |
|  |  | > $55,000 | Ref (0.00) | 0.01 | (-0.02,0.04) | 0.00 | (-0.03,0.03) | -0.03 | (-0.06,0.00) | -0.02 | (-0.05,0.01) | 0.021 | 0.162 |  |
| Sleep duration |  |  |  |  |  |  |  |  |  |  |  |  |  |  |
|  | Model 1 | ≤ 7 hours | Ref (0.00) | -0.01 | (-0.04,0.01) | -0.04 | **(-0.06,-0.01)*** | -0.06 | **(-0.08,-0.03)***** | -0.07 | **(-0.10,-0.05)***** | <0.001 | <0.001 | 0.114 |
|  |  | > 7 hours | Ref (0.00) | -0.02 | (-0.05,0.00) | -0.04 | **(-0.07,-0.02)***** | -0.06 | **(-0.08,-0.03)***** | -0.08 | **(-0.10,-0.06)***** | <0.001 | <0.001 |  |
|  | Model 2 | ≤ 7 hours | Ref (0.00) | 0.00 | (-0.02,0.03) | -0.02 | (-0.04,0.01) | -0.03 | **(-0.06,-0.01)**** | -0.04 | **(-0.06,-0.02)***** | <0.001 | 0.001 | 0.310 |
|  |  | > 7 hours | Ref (0.00) | -0.01 | (-0.03,0.02) | -0.02 | (-0.04,0.00) | -0.03 | **(-0.06,-0.01)*** | -0.04 | **(-0.06,-0.02)***** | <0.001 | <0.001 |  |
|  | Model 3 | ≤ 7 hours | Ref (0.00) | 0.01 | (-0.02,0.03) | -0.01 | (-0.04,0.01) | -0.03 | **(-0.05,-0.01)*** | -0.03 | **(-0.05,-0.01)*** | 0.001 | 0.013 | 0.452 |
|  |  | > 7 hours | Ref (0.00) | 0.01 | (-0.02,0.03) | -0.01 | (-0.03,0.01) | -0.01 | (-0.04,0.01) | -0.01 | (-0.03,0.01) | 0.086 | 0.219 |  |
|  | Model 4 | ≤ 7 hours | Ref (0.00) | 0.01 | (-0.02,0.04) | -0.01 | (-0.04,0.02) | -0.02 | (-0.05,0.00) | -0.02 | (-0.05,0.00) | 0.012 | 0.091 | 0.448 |
|  |  | > 7 hours | Ref (0.00) | 0.00 | (-0.02,0.03) | -0.01 | (-0.04,0.01) | -0.02 | (-0.04,0.01) | -0.02 | (-0.04,0.01) | 0.061 | 0.152 |  |
| Daily energy intake |  |  |  |  |  |  |  |  |  |  |  |  |  |  |
|  | Model 1 | Higher | Ref (0.00) | -0.01 | (-0.05,0.03) | -0.02 | (-0.06,0.01) | -0.05 | **(-0.08,-0.01)**** | -0.06 | **(-0.09,-0.03)***** | <0.001 | <0.001 | 0.001 |
|  |  | Lower | Ref (0.00) | -0.02 | (-0.04,0.00) | -0.04 | **(-0.07,-0.02)***** | -0.06 | **(-0.08,-0.04)***** | -0.08 | **(-0.10,-0.06)***** | <0.001 | <0.001 |  |
|  | Model 2 | Higher | Ref (0.00) | 0.01 | (-0.03,0.04) | -0.01 | (-0.04,0.03) | -0.02 | (-0.06,0.01) | -0.02 | (-0.05,0.01) | 0.030 | 0.190 | 0.015 |
|  |  | Lower | Ref (0.00) | 0.00 | (-0.02,0.02) | -0.02 | (-0.04,0.00) | -0.03 | **(-0.05,-0.01)**** | -0.04 | **(-0.06,-0.02)***** | <0.001 | <0.001 |  |
|  | Model 3 | Higher | Ref (0.00) | 0.01 | (-0.03,0.05) | 0.00 | (-0.04,0.04) | -0.02 | (-0.05,0.02) | -0.01 | (-0.04,0.02) | 0.136 | 0.475 | 0.012 |
|  |  | Lower | Ref (0.00) | 0.00 | (-0.02,0.02) | -0.02 | (-0.04,0.00) | -0.03 | (-0.05,0.00) | -0.04 | **(-0.05,-0.02)***** | <0.001 | 0.001 |  |
|  | Model 4 | Higher | Ref (0.00) | 0.01 | (-0.03,0.05) | 0.00 | (-0.04,0.04) | -0.02 | (-0.06,0.02) | -0.02 | (-0.05,0.02) | 0.073 | 0.337 | 0.012 |
|  |  | Lower | Ref (0.00) | 0.01 | (-0.02,0.03) | -0.01 | (-0.04,0.01) | -0.02 | (-0.05,0.00) | -0.03 | **(-0.05,-0.01)*** | 0.002 | 0.018 |  |
| Healthy eating index-2015 |  |  |  |  |  |  |  |  |  |  |  |  |  |  |
|  | Model 1 | < 30.53 | Ref (0.00) | -0.03 | (-0.05,0.00) | -0.04 | **(-0.07,-0.02)***** | -0.05 | **(-0.08,-0.02)***** | -0.06 | **(-0.08,-0.03)***** | <0.001 | <0.001 | 0.362 |
|  |  | ≥ 30.53 | Ref (0.00) | -0.01 | (-0.04,0.02) | -0.03 | **(-0.06,-0.01)*** | -0.06 | **(-0.08,-0.04)***** | -0.08 | **(-0.10,-0.06)***** | <0.001 | <0.001 |  |
|  | Model 2 | < 30.53 | Ref (0.00) | -0.01 | (-0.03,0.02) | -0.02 | (-0.05,0.00) | -0.03 | (-0.06,0.00) | -0.02 | (-0.05,0.00) | 0.025 | 0.031 | 0.214 |
|  |  | ≥ 30.53 | Ref (0.00) | 0.00 | (-0.02,0.03) | -0.01 | (-0.04,0.01) | -0.04 | **(-0.06,-0.02)***** | -0.04 | **(-0.06,-0.03)***** | <0.001 | <0.001 |  |
|  | Model 3 | < 30.53 | Ref (0.00) | 0.00 | (-0.02,0.02) | -0.02 | (-0.04,0.01) | -0.02 | (-0.05,0.01) | -0.01 | (-0.04,0.01) | 0.230 | 0.292 | 0.259 |
|  |  | ≥ 30.53 | Ref (0.00) | 0.01 | (-0.02,0.04) | -0.01 | (-0.03,0.02) | -0.02 | (-0.05,0.00) | -0.03 | **(-0.05,-0.01)**** | <0.001 | 0.009 |  |
|  | Model 4 | < 30.53 | Ref (0.00) | 0.00 | (-0.02,0.02) | -0.02 | (-0.04,0.01) | -0.02 | (-0.05,0.01) | -0.01 | (-0.04,0.02) | 0.396 | 0.448 | 0.257 |
|  |  | ≥ 30.53 | Ref (0.00) | 0.01 | (-0.02,0.04) | -0.01 | (-0.03,0.02) | -0.03 | (-0.05,0.00) | -0.03 | (-0.05,0.00) | <0.001 | 0.025 |  |
| Dietary supplements use |  |  |  |  |  |  |  |  |  |  |  |  |  |  |
|  | Model 1 | Yes | Ref (0.00) | -0.03 | (-0.06,0.00) | -0.03 | **(-0.06,-0.01)**** | -0.06 | **(-0.09,-0.03)***** | -0.08 | **(-0.10,-0.05)***** | <0.001 | <0.001 | 0.889 |
|  |  | No | Ref (0.00) | 0.00 | (-0.03,0.03) | -0.04 | **(-0.07,-0.02)**** | -0.05 | **(-0.07,-0.02)***** | -0.06 | **(-0.08,-0.04)***** | <0.001 | <0.001 |  |
|  | Model 2 | Yes | Ref (0.00) | -0.02 | (-0.04,0.01) | -0.02 | (-0.04,0.01) | -0.04 | **(-0.07,-0.01)**** | -0.05 | **(-0.07,-0.03)***** | <0.001 | <0.001 | 0.648 |
|  |  | No | Ref (0.00) | 0.02 | (-0.01,0.04) | -0.02 | (-0.05,0.00) | -0.02 | (-0.04,0.00) | -0.03 | **(-0.04,-0.01)*** | <0.001 | 0.013 |  |
|  | Model 3 | Yes | Ref (0.00) | -0.01 | (-0.04,0.02) | -0.01 | (-0.03,0.02) | -0.03 | (-0.06,0.00) | -0.03 | **(-0.05,-0.01)*** | 0.003 | 0.011 | 0.758 |
|  |  | No | Ref (0.00) | 0.02 | (0.00,0.05) | -0.01 | (-0.04,0.01) | -0.01 | (-0.03,0.01) | -0.01 | (-0.03,0.01) | 0.057 | 0.336 |  |
|  | Model 4 | Yes | Ref (0.00) | -0.01 | (-0.04,0.02) | -0.01 | (-0.04,0.01) | -0.04 | **(-0.07,-0.01)*** | -0.04 | **(-0.06,-0.01)**** | 0.002 | 0.008 | 0.752 |
|  |  | No | Ref (0.00) | 0.02 | (0.00,0.05) | -0.01 | (-0.04,0.02) | 0.00 | (-0.03,0.02) | 0.00 | (-0.03,0.02) | 0.214 | 0.767 |  |
| Nighttime fasting duration |  |  |  |  |  |  |  |  |  |  |  |  |  |  |
|  | Model 1 | < 12.58 hours | Ref (0.00) | -0.03 | (-0.06,0.01) | -0.05 | **(-0.08,-0.01)**** | -0.06 | **(-0.09,-0.02)**** | -0.07 | **(-0.11,-0.04)***** | <0.001 | <0.001 | 0.002 |
|  |  | ≥ 12.58 hours | Ref (0.00) | -0.01 | (-0.04,0.01) | -0.03 | **(-0.05,-0.01)*** | -0.05 | **(-0.08,-0.03)***** | -0.06 | **(-0.08,-0.03)***** | <0.001 | <0.001 |  |
|  | Model 2 | < 12.58 hours | Ref (0.00) | -0.01 | (-0.05,0.02) | -0.03 | (-0.06,0.01) | -0.04 | (-0.07,0.00) | -0.04 | **(-0.07,-0.01)**** | <0.001 | 0.008 | 0.016 |
|  |  | ≥ 12.58 hours | Ref (0.00) | 0.00 | (-0.02,0.03) | -0.01 | (-0.03,0.01) | -0.03 | (-0.05,0.00) | -0.02 | (-0.05,0.00) | 0.006 | 0.037 |  |
|  | Model 3 | < 12.58 hours | Ref (0.00) | 0.00 | (-0.04,0.03) | -0.02 | (-0.05,0.01) | -0.02 | (-0.06,0.01) | -0.03 | (-0.06,0.01) | 0.026 | 0.097 | 0.029 |
|  |  | ≥ 12.58 hours | Ref (0.00) | 0.01 | (-0.01,0.03) | 0.00 | (-0.02,0.02) | -0.01 | (-0.04,0.01) | -0.01 | (-0.03,0.02) | 0.240 | 0.569 |  |

^a^The variables adjusted in each model were the factors mentioned above except the stratification variables.

^b^Data were listed as the weighted beta estimates and 95% confidence intervals, with *p < 0.05, **p < 0.01, ***p < 0.001.

^c^Q, quintile.

^d^Ref, reference.

^e^Tests for trends based on the variables containing the median values for each quartile.

^f^*P*_test_ was the result of Bonfreni correction.

^g^Multiplicative interaction was assessed by adding interaction terms to the models.

**Supplementary table 7.** Association of DEF with KDM residual (years) stratified by variables of interest

|  |  |  | Daily eating frequency (times) | | | | | | | | |  |  |  |
| --- | --- | --- | --- | --- | --- | --- | --- | --- | --- | --- | --- | --- | --- | --- |
|  |  |  | Q1^c^ | Q2 | | Q3 | | Q4 | | Q5 | | *P*_trend_^e^ | *P*_test_^f^ | *P*_interaction_^g^ |
|  |  |  | β^b^ | β | 95% CI | β | 95% CI | β | 95% CI | β | 95% CI |  |  |  |
| Age |  |  |  |  |  |  |  |  |  |  |  |  |  |  |
|  | Model 1^a^ | > 60 years | Ref (0.00)^d^ | -0.90 | **(-1.78, -0.01)*** | -1.84 | **(-2.76, -0.92)***** | -1.52 | **(-2.39, -0.65)***** | -2.26 | **(-3.17, -1.36)***** | < 0.001 | < 0.001 | 0.002 |
|  |  | ≤ 60 years | Ref (0.00) | -0.88 | **(-1.37, -0.38)***** | -1.01 | **(-1.44, -0.58)***** | -1.46 | **(-1.88, -1.05)***** | -2.15 | **(-2.55, -1.75)***** | < 0.001 | < 0.001 |  |
|  | Model 2 | > 60 years | Ref (0.00) | -0.53 | (-1.35, 0.30) | -1.48 | **(-2.36, -0.61)***** | -1.18 | **(-2.02, -0.34)**** | -1.67 | **(-2.54, -0.81)***** | < 0.001 | < 0.001 | < 0.001 |
|  |  | ≤ 60 years | Ref (0.00) | -0.53 | **(-0.97, -0.08)*** | -0.55 | **(-0.96, -0.14)**** | -0.79 | **(-1.16, -0.41)***** | -1.27 | **(-1.63, -0.90)***** | < 0.001 | < 0.001 |  |
|  | Model 3 | > 60 years | Ref (0.00) | -0.37 | (-1.23, 0.48) | -1.31 | **(-2.22, -0.40)**** | -0.84 | (-1.71, 0.02) | -1.06 | **(-1.99, -0.13)*** | 0.037 | 0.025 | < 0.001 |
|  |  | ≤ 60 years | Ref (0.00) | -0.44 | **(-0.87, -0.01)*** | -0.53 | **(-0.93, -0.12)*** | -0.74 | **(-1.13, -0.35)***** | -1.21 | **(-1.63, -0.79)***** | < 0.001 | < 0.001 |  |
|  | Model 4 | > 60 years | Ref (0.00) | -0.20 | (-1.05, 0.66) | -1.03 | **(-1.95, -0.11)*** | -0.52 | (-1.39, 0.35) | -0.65 | (-1.62, 0.31) | 0.303 | 0.182 | < 0.001 |
|  |  | ≤ 60 years | Ref (0.00) | -0.33 | (-0.78, 0.12) | -0.39 | (-0.81, 0.04) | -0.57 | **(-1.01, -0.13)*** | -0.99 | **(-1.50, -0.49)***** | < 0.001 | < 0.001 |  |
| Sex |  |  |  |  |  |  |  |  |  |  |  |  |  |  |
|  | Model 1 | Male | Ref (0.00) | -0.61 | (-1.23, 0.02) | -0.83 | **(-1.35, -0.30)**** | -1.02 | **(-1.62, -0.42)***** | -1.80 | **(-2.34, -1.26)***** | < 0.001 | < 0.001 | 0.893 |
|  |  | Female | Ref (0.00) | -0.65 | **(-1.11, -0.19)**** | -0.98 | **(-1.50, -0.45)***** | -1.31 | **(-1.81, -0.81)***** | -1.67 | **(-2.06, -1.29)***** | < 0.001 | < 0.001 |  |
|  | Model 2 | Male | Ref (0.00) | -0.28 | (-0.85, 0.29) | -0.41 | (-0.88, 0.06) | -0.43 | (-0.98, 0.12) | -0.95 | **(-1.45, -0.46)***** | < 0.001 | < 0.001 | 0.191 |
|  |  | Female | Ref (0.00) | -0.24 | (-0.66, 0.18) | -0.50 | **(-0.99, -0.01)*** | -0.71 | **(-1.19, -0.24)**** | -0.84 | **(-1.20, -0.48)***** | < 0.001 | < 0.001 |  |
|  | Model 3 | Male | Ref (0.00) | -0.11 | (-0.68, 0.46) | -0.26 | (-0.74, 0.21) | -0.22 | (-0.74, 0.29) | -0.65 | **(-1.16, -0.14)*** | 0.008 | 0.014 | 0.158 |
|  |  | Female | Ref (0.00) | -0.18 | (-0.59, 0.24) | -0.43 | (-0.93, 0.06) | -0.62 | **(-1.12, -0.11)*** | -0.68 | **(-1.08, -0.28)***** | < 0.001 | 0.001 |  |
|  | Model 4 | Male | Ref (0.00) | -0.08 | (-0.67, 0.51) | -0.22 | (-0.71, 0.27) | -0.18 | (-0.76, 0.41) | -0.59 | (-1.17, 0.00) | 0.029 | 0.050 | 0.158 |
|  |  | Female | Ref (0.00) | -0.17 | (-0.61, 0.28) | -0.42 | (-0.94, 0.10) | -0.60 | **(-1.14, -0.07)*** | -0.66 | **(-1.15, -0.18)**** | 0.003 | 0.008 |  |
| Race |  |  |  |  |  |  |  |  |  |  |  |  |  |  |
|  | Model 1 | Non-hispanic white | Ref (0.00) | -0.65 | **(-1.19, -0.11)*** | -0.84 | **(-1.36, -0.32)**** | -1.09 | **(-1.61, -0.58)***** | -1.59 | **(-2.06, -1.12)***** | < 0.001 | < 0.001 | 0.146 |
|  |  | Others | Ref (0.00) | -0.51 | (-1.01, 0.00) | -0.78 | **(-1.23, -0.33)***** | -1.09 | **(-1.61, -0.57)***** | -1.69 | **(-2.12, -1.27)***** | < 0.001 | < 0.001 |  |
|  | Model 2 | Non-hispanic white | Ref (0.00) | -0.23 | (-0.68, 0.22) | -0.37 | (-0.84, 0.09) | -0.49 | **(-0.95, -0.02)*** | -0.76 | **(-1.17, -0.35)***** | < 0.001 | < 0.001 | 0.151 |
|  |  | Others | Ref (0.00) | -0.28 | (-0.77, 0.20) | -0.49 | **(-0.92, -0.06)*** | -0.68 | **(-1.17, -0.18)**** | -1.10 | **(-1.51, -0.70)***** | < 0.001 | < 0.001 |  |
|  | Model 3 | Non-hispanic white | Ref (0.00) | -0.06 | (-0.50, 0.38) | -0.20 | (-0.67, 0.27) | -0.24 | (-0.70, 0.23) | -0.37 | (-0.80, 0.05) | 0.061 | 0.084 | 0.196 |
|  |  | Others | Ref (0.00) | -0.24 | (-0.71, 0.23) | -0.51 | **(-0.92, -0.10)*** | -0.66 | **(-1.16, -0.17)**** | -1.10 | **(-1.51, -0.68)***** | < 0.001 | < 0.001 |  |
|  | Model 4 | Non-hispanic white | Ref (0.00) | -0.06 | (-0.53, 0.41) | -0.20 | (-0.71, 0.32) | -0.23 | (-0.77, 0.30) | -0.37 | (-0.92, 0.18) | 0.139 | 0.184 | 0.195 |
|  |  | Others | Ref (0.00) | -0.22 | (-0.71, 0.28) | -0.48 | **(-0.92, -0.04)*** | -0.62 | **(-1.19, -0.06)*** | -1.05 | **(-1.55, -0.55)***** | < 0.001 | < 0.001 |  |
| BMI |  |  |  |  |  |  |  |  |  |  |  |  |  |  |
|  | Model 1 | < 30 kg/m^2^ | Ref (0.00) | -0.73 | **(-1.19, -0.26)**** | -0.93 | **(-1.36, -0.49)***** | -0.98 | **(-1.42, -0.55)***** | -1.38 | **(-1.76, -1.00)***** | < 0.001 | < 0.001 | 0.888 |
|  |  | ≥ 30 kg/m^2^ | Ref (0.00) | 0.01 | (-0.60, 0.61) | -0.46 | (-1.01, 0.09) | -0.77 | **(-1.42, -0.13)*** | -1.26 | **(-1.79, -0.73)***** | < 0.001 | < 0.001 |  |
|  | Model 2 | < 30 kg/m^2^ | Ref (0.00) | -0.56 | **(-1.01, -0.11)*** | -0.68 | **(-1.11, -0.26)**** | -0.67 | **(-1.09, -0.26)**** | -1.02 | **(-1.38, -0.65)***** | < 0.001 | < 0.001 | 0.926 |
|  |  | ≥ 30 kg/m^2^ | Ref (0.00) | 0.09 | (-0.49, 0.68) | -0.32 | (-0.86, 0.22) | -0.63 | (-1.28, 0.01) | -0.98 | **(-1.48, -0.48)***** | < 0.001 | < 0.001 |  |
|  | Model 3 | < 30 kg/m^2^ | Ref (0.00) | -0.38 | (-0.83, 0.07) | -0.51 | **(-0.95, -0.08)*** | -0.43 | **(-0.84, -0.02)*** | -0.66 | **(-1.06, -0.26)***** | 0.005 | 0.001 | 0.656 |
|  |  | ≥ 30 kg/m^2^ | Ref (0.00) | 0.15 | (-0.41, 0.71) | -0.27 | (-0.79, 0.25) | -0.53 | (-1.13, 0.07) | -0.83 | **(-1.33, -0.33)***** | < 0.001 | < 0.001 |  |
|  | Model 4 | < 30 kg/m^2^ | Ref (0.00) | -0.35 | (-0.81, 0.10) | -0.48 | **(-0.91, -0.04)*** | -0.39 | (-0.82, 0.05) | -0.61 | **(-1.07, -0.15)**** | 0.037 | 0.010 | 0.656 |
|  |  | ≥ 30 kg/m^2^ | Ref (0.00) | 0.16 | (-0.45, 0.78) | -0.25 | (-0.85, 0.36) | -0.50 | (-1.16, 0.16) | -0.79 | **(-1.48, -0.10)*** | 0.003 | < 0.001 |  |
| Smoking |  |  |  |  |  |  |  |  |  |  |  |  |  |  |
|  | Model 1 | Yes | Ref (0.00) | -0.52 | (-1.05, 0.00) | -0.54 | (-1.08, 0.01) | -0.86 | **(-1.42, -0.30)**** | -1.38 | **(-1.87, -0.90)***** | < 0.001 | < 0.001 | 0.011 |
|  |  | No | Ref (0.00) | -0.74 | **(-1.32, -0.15)*** | -1.14 | **(-1.59, -0.69)***** | -1.41 | **(-1.88, -0.94)***** | -2.00 | **(-2.41, -1.59)***** | < 0.001 | < 0.001 |  |
|  | Model 2 | Yes | Ref (0.00) | -0.19 | (-0.67, 0.28) | -0.16 | (-0.65, 0.34) | -0.32 | (-0.82, 0.19) | -0.59 | **(-1.02, -0.15)**** | 0.004 | 0.009 | 0.035 |
|  |  | No | Ref (0.00) | -0.31 | (-0.84, 0.21) | -0.62 | **(-1.04, -0.21)**** | -0.79 | **(-1.24, -0.35)***** | -1.13 | **(-1.52, -0.74)***** | < 0.001 | < 0.001 |  |
|  | Model 3 | Yes | Ref (0.00) | -0.06 | (-0.52, 0.41) | -0.04 | (-0.55, 0.47) | -0.09 | (-0.58, 0.40) | -0.27 | (-0.75, 0.22) | 0.216 | 0.275 | 0.048 |
|  |  | No | Ref (0.00) | -0.18 | (-0.70, 0.34) | -0.49 | **(-0.90, -0.08)*** | -0.62 | **(-1.07, -0.16)**** | -0.84 | **(-1.27, -0.42)***** | < 0.001 | < 0.001 |  |
|  | Model 4 | Yes | Ref (0.00) | -0.01 | (-0.50, 0.48) | 0.02 | (-0.53, 0.57) | -0.03 | (-0.56, 0.51) | -0.19 | (-0.75, 0.37) | 0.397 | 0.508 | 0.047 |
|  |  | No | Ref (0.00) | -0.19 | (-0.70, 0.33) | -0.50 | **(-0.91, -0.08)*** | -0.63 | **(-1.13, -0.13)*** | -0.86 | **(-1.34, -0.38)***** | 0.001 | 0.001 |  |
| Drinking |  |  |  |  |  |  |  |  |  |  |  |  |  |  |
|  | Model 1 | Yes | Ref (0.00) | -0.53 | **(-1.00, -0.05)*** | -0.85 | **(-1.27, -0.42)***** | -1.17 | **(-1.65, -0.68)***** | -1.70 | **(-2.06, -1.33)***** | < 0.001 | < 0.001 | 0.876 |
|  |  | No | Ref (0.00) | -0.91 | (-1.86, 0.03) | -0.59 | (-1.31, 0.13) | -1.18 | **(-1.96, -0.40)**** | -1.68 | **(-2.34, -1.01)***** | < 0.001 | < 0.001 |  |
|  | Model 2 | Yes | Ref (0.00) | -0.11 | (-0.53, 0.32) | -0.39 | **(-0.76, -0.02)*** | -0.59 | **(-1.04, -0.14)**** | -0.84 | **(-1.17, -0.52)***** | < 0.001 | < 0.001 | 0.705 |
|  |  | No | Ref (0.00) | -0.62 | (-1.51, 0.28) | -0.19 | (-0.89, 0.51) | -0.63 | (-1.40, 0.14) | -0.97 | **(-1.62, -0.31)**** | 0.002 | 0.005 |  |
|  | Model 3 | Yes | Ref (0.00) | 0.04 | (-0.38, 0.46) | -0.27 | (-0.65, 0.11) | -0.38 | (-0.84, 0.07) | -0.56 | **(-0.91, -0.20)**** | 0.001 | 0.003 | 0.713 |
|  |  | No | Ref (0.00) | -0.53 | (-1.41, 0.35) | -0.01 | (-0.70, 0.68) | -0.45 | (-1.22, 0.31) | -0.57 | (-1.29, 0.14) | 0.109 | 0.114 |  |
|  | Model 4 | Yes | Ref (0.00) | 0.09 | (-0.34, 0.53) | -0.20 | (-0.60, 0.20) | -0.29 | (-0.80, 0.21) | -0.44 | (-0.88, 0.01) | 0.019 | 0.053 | 0.711 |
|  |  | No | Ref (0.00) | -0.62 | (-1.52, 0.28) | -0.14 | (-0.83, 0.56) | -0.61 | (-1.40, 0.19) | -0.77 | **(-1.52, -0.02)*** | 0.036 | 0.045 |  |
| Exercise |  |  |  |  |  |  |  |  |  |  |  |  |  |  |
|  | Model 1 | Yes | Ref (0.00) | -0.34 | (-0.97, 0.30) | -0.77 | **(-1.32, -0.21)**** | -0.84 | **(-1.47, -0.21)**** | -1.45 | **(-1.96, -0.94)***** | < 0.001 | < 0.001 | 0.693 |
|  |  | No | Ref (0.00) | -0.75 | **(-1.26, -0.25)**** | -0.87 | **(-1.40, -0.34)**** | -1.29 | **(-1.79, -0.79)***** | -1.75 | **(-2.21, -1.28)***** | < 0.001 | < 0.001 |  |
|  | Model 2 | Yes | Ref (0.00) | 0.03 | (-0.53, 0.59) | -0.23 | (-0.73, 0.27) | -0.19 | (-0.72, 0.35) | -0.57 | **(-1.03, -0.12)*** | 0.007 | 0.014 | 0.377 |
|  |  | No | Ref (0.00) | -0.40 | (-0.84, 0.04) | -0.53 | **(-1.01, -0.06)*** | -0.78 | **(-1.25, -0.32)***** | -1.05 | **(-1.45, -0.64)***** | < 0.001 | < 0.001 |  |
|  | Model 3 | Yes | Ref (0.00) | -0.01 | (-0.55, 0.52) | -0.25 | (-0.74, 0.25) | -0.18 | (-0.70, 0.35) | -0.59 | **(-1.03, -0.14)*** | 0.008 | 0.011 | 0.392 |
|  |  | No | Ref (0.00) | -0.17 | (-0.59, 0.27) | -0.34 | (-0.81, 0.14) | -0.50 | **(-0.95, -0.04)*** | -0.60 | **(-1.03, -0.17)**** | 0.004 | 0.006 |  |
|  | Model 4 | Yes | Ref (0.00) | -0.01 | (-0.58, 0.57) | -0.24 | (-0.77, 0.30) | -0.17 | (-0.75, 0.42) | -0.57 | **(-1.10, -0.05)*** | 0.015 | 0.033 | 0.392 |
|  |  | No | Ref (0.00) | -0.15 | (-0.60, 0.29) | -0.32 | (-0.83, 0.18) | -0.48 | (-1.00, 0.04) | -0.58 | **(-1.11, -0.05)*** | 0.026 | 0.033 |  |
| Education |  |  |  |  |  |  |  |  |  |  |  |  |  |  |
|  | Model 1 | Above high school | Ref (0.00) | -0.77 | **(-1.37, -0.17)*** | -0.73 | **(-1.21, -0.24)**** | -1.20 | **(-1.77, -0.63)***** | -1.68 | **(-2.13, -1.23)***** | < 0.001 | < 0.001 | 0.571 |
|  |  | Others | Ref (0.00) | -0.25 | (-0.83, 0.33) | -0.84 | **(-1.38, -0.31)**** | -0.67 | **(-1.23, -0.10)*** | -1.29 | **(-1.79, -0.78)***** | < 0.001 | < 0.001 |  |
|  | Model 2 | Above high school | Ref (0.00) | -0.42 | (-0.97, 0.12) | -0.40 | (-0.84, 0.05) | -0.67 | **(-1.22, -0.11)*** | -0.96 | **(-1.40, -0.52)***** | < 0.001 | < 0.001 | 0.691 |
|  |  | Others | Ref (0.00) | -0.07 | (-0.61, 0.46) | -0.59 | **(-1.09, -0.09)*** | -0.41 | (-0.94, 0.12) | -0.79 | **(-1.26, -0.31)***** | < 0.001 | 0.001 |  |
|  | Model 3 | Above high school | Ref (0.00) | -0.31 | (-0.82, 0.21) | -0.31 | (-0.75, 0.13) | -0.53 | (-1.08, 0.03) | -0.73 | **(-1.18, -0.28)**** | 0.002 | 0.002 | 0.728 |
|  |  | Others | Ref (0.00) | 0.10 | (-0.44, 0.63) | -0.38 | (-0.91, 0.14) | -0.12 | (-0.66, 0.42) | -0.39 | (-0.90, 0.13) | 0.088 | 0.140 |  |
|  | Model 4 | Above high school | Ref (0.00) | -0.33 | (-0.85, 0.20) | -0.33 | (-0.79, 0.13) | -0.55 | (-1.17, 0.06) | -0.77 | **(-1.30, -0.23)**** | 0.008 | 0.005 | 0.727 |
|  |  | Others | Ref (0.00) | 0.16 | (-0.40, 0.72) | -0.29 | (-0.82, 0.24) | -0.01 | (-0.57, 0.55) | -0.25 | (-0.81, 0.32) | 0.255 | 0.383 |  |
| Income |  |  |  |  |  |  |  |  |  |  |  |  |  |  |
|  | Model 1 | ≤ $55,000 | Ref (0.00) | -0.50 | (-1.02, 0.02) | -1.00 | **(-1.48, -0.52)***** | -1.09 | **(-1.67, -0.51)***** | -1.78 | **(-2.25, -1.32)***** | < 0.001 | < 0.001 | 0.080 |
|  |  | > $55,000 | Ref (0.00) | -0.69 | **(-1.33, -0.05)*** | -0.64 | **(-1.19, -0.08)*** | -1.25 | **(-1.84, -0.66)***** | -1.41 | **(-1.90, -0.91)***** | < 0.001 | < 0.001 |  |
|  | Model 2 | ≤ $55,000 | Ref (0.00) | -0.13 | (-0.59, 0.33) | -0.57 | **(-1.01, -0.13)*** | -0.59 | **(-1.10, -0.07)*** | -0.98 | **(-1.41, -0.55)***** | < 0.001 | < 0.001 | 0.224 |
|  |  | > $55,000 | Ref (0.00) | -0.42 | (-0.99, 0.15) | -0.32 | (-0.83, 0.19) | -0.75 | **(-1.33, -0.17)*** | -0.80 | **(-1.28, -0.33)***** | 0.001 | 0.001 |  |
|  | Model 3 | ≤ $55,000 | Ref (0.00) | 0.02 | (-0.44, 0.49) | -0.43 | (-0.88, 0.02) | -0.36 | (-0.90, 0.18) | -0.65 | **(-1.13, -0.17)**** | 0.003 | 0.008 | 0.308 |
|  |  | > $55,000 | Ref (0.00) | -0.30 | (-0.87, 0.26) | -0.19 | (-0.69, 0.31) | -0.57 | **(-1.12, -0.02)*** | -0.56 | **(-1.02, -0.09)*** | 0.022 | 0.021 |  |
|  | Model 4 | ≤ $55,000 | Ref (0.00) | 0.03 | (-0.45, 0.51) | -0.42 | (-0.90, 0.05) | -0.35 | (-0.93, 0.22) | -0.64 | **(-1.20, -0.08)*** | 0.010 | 0.025 | 0.313 |
|  |  | > $55,000 | Ref (0.00) | -0.31 | (-0.90, 0.29) | -0.20 | (-0.75, 0.36) | -0.57 | (-1.18, 0.03) | -0.56 | (-1.14, 0.03) | 0.059 | 0.061 |  |
| Sleep duration |  |  |  |  |  |  |  |  |  |  |  |  |  |  |
|  | Model 1 | ≤ 7 hours | Ref (0.00) | -0.51 | (-1.10, 0.09) | -0.85 | **(-1.47, -0.24)**** | -1.00 | **(-1.52, -0.48)***** | -1.54 | **(-2.03, -1.04)***** | < 0.001 | < 0.001 | 0.052 |
|  |  | > 7 hours | Ref (0.00) | -0.72 | **(-1.24, -0.20)**** | -0.95 | **(-1.44, -0.46)***** | -1.28 | **(-1.83, -0.73)***** | -1.87 | **(-2.30, -1.44)***** | < 0.001 | < 0.001 |  |
|  | Model 2 | ≤ 7 hours | Ref (0.00) | -0.19 | (-0.72, 0.35) | -0.43 | (-0.95, 0.09) | -0.43 | (-0.93, 0.07) | -0.74 | **(-1.19, -0.28)**** | 0.001 | 0.002 | 0.115 |
|  |  | > 7 hours | Ref (0.00) | -0.32 | (-0.77, 0.14) | -0.47 | (-0.94, 0.00) | -0.67 | **(-1.19, -0.15)*** | -1.02 | **(-1.43, -0.60)***** | < 0.001 | < 0.001 |  |
|  | Model 3 | ≤ 7 hours | Ref (0.00) | -0.10 | (-0.61, 0.42) | -0.40 | (-0.93, 0.13) | -0.38 | (-0.89, 0.13) | -0.59 | **(-1.09, -0.10)*** | 0.015 | 0.019 | 0.131 |
|  |  | > 7 hours | Ref (0.00) | -0.15 | (-0.60, 0.29) | -0.28 | (-0.74, 0.19) | -0.37 | (-0.89, 0.14) | -0.61 | **(-1.06, -0.15)**** | 0.007 | 0.009 |  |
|  | Model 4 | ≤ 7 hours | Ref (0.00) | 0.02 | (-0.53, 0.56) | -0.24 | (-0.80, 0.31) | -0.20 | (-0.77, 0.38) | -0.35 | (-0.92, 0.22) | 0.154 | 0.220 | 0.130 |
|  |  | > 7 hours | Ref (0.00) | -0.22 | (-0.68, 0.24) | -0.37 | (-0.86, 0.13) | -0.48 | (-1.02, 0.06) | -0.74 | **(-1.28, -0.21)**** | 0.006 | 0.007 |  |
| Daily energy intake |  |  |  |  |  |  |  |  |  |  |  |  |  |  |
|  | Model 1 | Higher | Ref (0.00) | -0.52 | (-1.30, 0.26) | -0.37 | (-1.08, 0.33) | -0.81 | **(-1.41, -0.22)**** | -1.52 | **(-2.12, -0.91)***** | < 0.001 | < 0.001 | 0.018 |
|  |  | Lower | Ref (0.00) | -0.67 | **(-1.15, -0.19)**** | -1.14 | **(-1.59, -0.69)***** | -1.35 | **(-1.87, -0.82)***** | -1.84 | **(-2.29, -1.39)***** | < 0.001 | < 0.001 |  |
|  | Model 2 | Higher | Ref (0.00) | -0.22 | (-0.93, 0.50) | 0.03 | (-0.63, 0.68) | -0.27 | (-0.85, 0.32) | -0.63 | **(-1.19, -0.08)*** | 0.002 | 0.026 | 0.183 |
|  |  | Lower | Ref (0.00) | -0.25 | (-0.67, 0.18) | -0.62 | **(-1.02, -0.22)**** | -0.66 | **(-1.17, -0.15)*** | -0.93 | **(-1.35, -0.51)***** | < 0.001 | < 0.001 |  |
|  | Model 3 | Higher | Ref (0.00) | -0.10 | (-0.81, 0.61) | 0.12 | (-0.55, 0.79) | -0.10 | (-0.68, 0.48) | -0.43 | (-1.01, 0.15) | 0.025 | 0.146 | 0.165 |
|  |  | Lower | Ref (0.00) | -0.15 | (-0.57, 0.28) | -0.56 | **(-0.96, -0.15)**** | -0.56 | **(-1.07, -0.06)*** | -0.78 | **(-1.20, -0.37)***** | < 0.001 | < 0.001 |  |
|  | Model 4 | Higher | Ref (0.00) | -0.15 | (-0.87, 0.57) | 0.04 | (-0.65, 0.74) | -0.20 | (-0.82, 0.42) | -0.56 | (-1.22, 0.11) | 0.021 | 0.101 | 0.160 |
|  |  | Lower | Ref (0.00) | -0.08 | (-0.53, 0.38) | -0.46 | **(-0.89, -0.02)*** | -0.45 | (-1.00, 0.11) | -0.64 | **(-1.13, -0.15)*** | 0.005 | 0.012 |  |
| Healthy eating index-2015 |  |  |  |  |  |  |  |  |  |  |  |  |  |  |
|  | Model 1 | < 30.53 | Ref (0.00) | -0.94 | **(-1.41, -0.47)***** | -1.02 | **(-1.58, -0.46)***** | -1.05 | **(-1.59, -0.51)***** | -1.48 | **(-2.00, -0.96)***** | < 0.001 | < 0.001 | 0.543 |
|  |  | ≥ 30.53 | Ref (0.00) | -0.25 | (-0.85, 0.34) | -0.65 | **(-1.16, -0.14)*** | -1.12 | **(-1.66, -0.59)***** | -1.65 | **(-2.13, -1.17)***** | < 0.001 | < 0.001 |  |
|  | Model 2 | < 30.53 | Ref (0.00) | -0.48 | **(-0.89, -0.07)*** | -0.56 | **(-1.06, -0.06)*** | -0.49 | (-0.99, 0.02) | -0.70 | **(-1.17, -0.23)**** | 0.012 | 0.040 | 0.332 |
|  |  | ≥ 30.53 | Ref (0.00) | 0.00 | (-0.56, 0.55) | -0.27 | (-0.74, 0.20) | -0.59 | **(-1.09, -0.08)*** | -0.88 | **(-1.34, -0.43)***** | < 0.001 | < 0.001 |  |
|  | Model 3 | < 30.53 | Ref (0.00) | -0.38 | (-0.77, 0.01) | -0.51 | (-1.03, 0.00) | -0.39 | (-0.91, 0.12) | -0.58 | **(-1.06, -0.09)*** | 0.050 | 0.021 | 0.379 |
|  |  | ≥ 30.53 | Ref (0.00) | 0.09 | (-0.47, 0.66) | -0.14 | (-0.61, 0.33) | -0.40 | (-0.94, 0.14) | -0.63 | **(-1.13, -0.13)*** | 0.002 | 0.014 |  |
|  | Model 4 | < 30.53 | Ref (0.00) | -0.39 | (-0.79, 0.01) | -0.53 | **(-1.03, -0.02)*** | -0.41 | (-0.93, 0.10) | -0.60 | **(-1.13, -0.07)*** | 0.077 | 0.028 | 0.376 |
|  |  | ≥ 30.53 | Ref (0.00) | 0.13 | (-0.46, 0.72) | -0.09 | (-0.60, 0.42) | -0.35 | (-0.95, 0.26) | -0.55 | (-1.17, 0.06) | 0.015 | 0.078 |  |
| Dietary supplements use |  |  |  |  |  |  |  |  |  |  |  |  |  |  |
|  | Model 1 | Yes | Ref (0.00) | -0.64 | **(-1.24, -0.04)*** | -0.72 | **(-1.21, -0.22)**** | -1.29 | **(-1.88, -0.71)***** | -1.61 | **(-2.05, -1.16)***** | < 0.001 | < 0.001 | 0.673 |
|  |  | No | Ref (0.00) | -0.51 | (-1.02, 0.01) | -0.99 | **(-1.49, -0.48)***** | -0.76 | **(-1.29, -0.24)**** | -1.61 | **(-2.07, -1.14)***** | < 0.001 | < 0.001 |  |
|  | Model 2 | Yes | Ref (0.00) | -0.32 | (-0.90, 0.25) | -0.30 | (-0.77, 0.16) | -0.76 | **(-1.32, -0.21)**** | -0.88 | **(-1.30, -0.46)***** | < 0.001 | < 0.001 | 0.479 |
|  |  | No | Ref (0.00) | -0.12 | (-0.58, 0.35) | -0.57 | **(-1.00, -0.14)**** | -0.22 | (-0.68, 0.25) | -0.81 | **(-1.21, -0.40)***** | < 0.001 | < 0.001 |  |
|  | Model 3 | Yes | Ref (0.00) | -0.13 | (-0.68, 0.42) | -0.14 | (-0.61, 0.34) | -0.52 | (-1.07, 0.03) | -0.51 | **(-0.95, -0.08)*** | 0.011 | 0.022 | 0.687 |
|  |  | No | Ref (0.00) | -0.11 | (-0.57, 0.35) | -0.55 | **(-0.97, -0.12)*** | -0.19 | (-0.67, 0.29) | -0.74 | **(-1.20, -0.29)**** | 0.001 | 0.020 |  |
|  | Model 4 | Yes | Ref (0.00) | -0.16 | (-0.73, 0.41) | -0.18 | (-0.65, 0.30) | -0.56 | (-1.14, 0.02) | -0.58 | **(-1.10, -0.05)*** | 0.021 | 0.032 | 0.686 |
|  |  | No | Ref (0.00) | -0.05 | (-0.53, 0.44) | -0.46 | **(-0.90, -0.02)*** | -0.09 | (-0.61, 0.43) | -0.62 | **(-1.14, -0.10)*** | 0.016 | 0.020 |  |
| Nighttime fasting duration |  |  |  |  |  |  |  |  |  |  |  |  |  |  |
|  | Model 1 | < 12.58 hours | Ref (0.00) | -0.89 | **(-1.66, -0.11)*** | -0.90 | **(-1.73, -0.08)*** | -1.16 | **(-1.92, -0.39)**** | -1.69 | **(-2.41, -0.97)***** | < 0.001 | < 0.001 | 0.002 |
|  |  | ≥ 12.58 hours | Ref (0.00) | -0.44 | (-0.91, 0.03) | -0.78 | **(-1.24, -0.33)***** | -0.98 | **(-1.60, -0.35)**** | -1.44 | **(-1.92, -0.96)***** | < 0.001 | < 0.001 |  |
|  | Model 2 | < 12.58 hours | Ref (0.00) | -0.50 | (-1.16, 0.16) | -0.44 | (-1.18, 0.30) | -0.65 | (-1.34, 0.04) | -0.89 | **(-1.53, -0.24)**** | 0.002 | 0.008 | 0.008 |
|  |  | ≥ 12.58 hours | Ref (0.00) | -0.11 | (-0.54, 0.31) | -0.41 | (-0.83, 0.00) | -0.34 | (-0.94, 0.26) | -0.73 | **(-1.18, -0.27)**** | 0.001 | 0.002 |  |
|  | Model 3 | < 12.58 hours | Ref (0.00) | -0.39 | (-1.05, 0.27) | -0.37 | (-1.09, 0.36) | -0.47 | (-1.16, 0.22) | -0.64 | (-1.29, 0.01) | 0.051 | 0.053 | 0.015 |
|  |  | ≥ 12.58 hours | Ref (0.00) | 0.03 | (-0.39, 0.45) | -0.28 | (-0.71, 0.16) | -0.17 | (-0.75, 0.41) | -0.46 | (-0.93, 0.02) | 0.039 | 0.061 |  |

^a^The variables adjusted in each model were the factors mentioned above except the stratification variables.

^b^Data were listed as the weighted beta estimates and 95% confidence intervals, with *p < 0.05, **p < 0.01, ***p < 0.001.

^c^Q, quintile.

^d^Ref, reference.

^e^Tests for trends based on the variables containing the median values for each quartile.

^f^*P*_test_ was the result of Bonfreni correction.

^g^Multiplicative interaction was assessed by adding interaction terms to the models.

**Supplementary table 8.** Association of DEF with PA residual (years) stratified by variables of interest

|  |  |  | Daily eating frequency (times) | | | | | | | | |  |  |  |
| --- | --- | --- | --- | --- | --- | --- | --- | --- | --- | --- | --- | --- | --- | --- |
|  |  |  | Q1^c^ | Q2 | | Q3 | | Q4 | | Q5 | | *P*_trend_^e^ | *P*_test_^f^ | *P*_interaction_^g^ |
|  |  |  | β^b^ | β | 95% CI | β | 95% CI | β | 95% CI | β | 95% CI |  |  |  |
| Age |  |  |  |  |  |  |  |  |  |  |  |  |  |  |
|  | Model 1^a^ | > 60 years | Ref (0.00)^d^ | -0.40 | (-0.91, 0.10) | -0.74 | **(-1.20, -0.29)**** | -0.86 | **(-1.35, -0.36)***** | -1.22 | **(-1.70, -0.73)***** | < 0.001 | < 0.001 | 0.001 |
|  |  | ≤ 60 years | Ref (0.00) | -0.55 | **(-0.87, -0.23)***** | -0.75 | **(-1.03, -0.46)***** | -0.97 | **(-1.23, -0.70)***** | -1.29 | **(-1.54, -1.04)***** | < 0.001 | < 0.001 |  |
|  | Model 2 | > 60 years | Ref (0.00) | -0.09 | (-0.56, 0.39) | -0.43 | (-0.87, 0.01) | -0.56 | **(-1.06, -0.06)*** | -0.70 | **(-1.17, -0.23)**** | 0.002 | 0.004 | < 0.001 |
|  |  | ≤ 60 years | Ref (0.00) | -0.32 | **(-0.59, -0.04)*** | -0.44 | **(-0.69, -0.20)***** | -0.51 | **(-0.74, -0.29)***** | -0.70 | **(-0.90, -0.49)***** | < 0.001 | < 0.001 |  |
|  | Model 3 | > 60 years | Ref (0.00) | -0.02 | (-0.50, 0.47) | -0.35 | (-0.81, 0.10) | -0.39 | (-0.91, 0.13) | -0.35 | (-0.87, 0.18) | 0.137 | 0.194 | < 0.001 |
|  |  | ≤ 60 years | Ref (0.00) | -0.25 | (-0.51, 0.01) | -0.42 | **(-0.66, -0.18)***** | -0.48 | **(-0.71, -0.26)***** | -0.65 | **(-0.88, -0.43)***** | < 0.001 | < 0.001 |  |
|  | Model 4 | > 60 years | Ref (0.00) | -0.03 | (-0.57, 0.51) | -0.38 | (-0.87, 0.12) | -0.42 | (-1.02, 0.18) | -0.38 | (-1.00, 0.24) | 0.167 | 0.225 | < 0.001 |
|  |  | ≤ 60 years | Ref (0.00) | -0.27 | (-0.55, 0.00) | -0.45 | **(-0.69, -0.22)***** | -0.52 | **(-0.77, -0.27)***** | -0.70 | **(-0.95, -0.46)***** | < 0.001 | < 0.001 |  |
| Sex |  |  |  |  |  |  |  |  |  |  |  |  |  |  |
|  | Model 1 | Male | Ref (0.00) | -0.31 | (-0.66, 0.05) | -0.41 | **(-0.71, -0.12)**** | -0.52 | **(-0.86, -0.17)**** | -0.96 | **(-1.26, -0.66)***** | < 0.001 | < 0.001 | 0.261 |
|  |  | Female | Ref (0.00) | -0.51 | **(-0.86, -0.16)**** | -0.79 | **(-1.14, -0.44)***** | -1.09 | **(-1.40, -0.79)***** | -1.24 | **(-1.51, -0.97)***** | < 0.001 | < 0.001 |  |
|  | Model 2 | Male | Ref (0.00) | -0.13 | (-0.45, 0.20) | -0.18 | (-0.44, 0.09) | -0.19 | (-0.49, 0.12) | -0.48 | **(-0.76, -0.21)***** | 0.001 | 0.001 | 0.878 |
|  |  | Female | Ref (0.00) | -0.15 | (-0.45, 0.16) | -0.37 | **(-0.68, -0.06)*** | -0.58 | **(-0.84, -0.31)***** | -0.51 | **(-0.75, -0.26)***** | < 0.001 | < 0.001 |  |
|  | Model 3 | Male | Ref (0.00) | 0.02 | (-0.29, 0.33) | -0.04 | (-0.31, 0.22) | -0.01 | (-0.31, 0.28) | -0.23 | (-0.52, 0.05) | 0.071 | 0.011 | 0.760 |
|  |  | Female | Ref (0.00) | -0.13 | (-0.44, 0.18) | -0.37 | **(-0.68, -0.06)*** | -0.56 | **(-0.84, -0.29)***** | -0.46 | **(-0.73, -0.20)***** | < 0.001 | 0.001 |  |
|  | Model 4 | Male | Ref (0.00) | -0.01 | (-0.34, 0.32) | -0.08 | (-0.35, 0.19) | -0.06 | (-0.40, 0.27) | -0.29 | (-0.61, 0.03) | 0.040 | 0.072 | 0.754 |
|  |  | Female | Ref (0.00) | -0.22 | (-0.55, 0.10) | -0.50 | **(-0.82, -0.18)**** | -0.72 | **(-1.02, -0.42)***** | -0.67 | **(-0.98, -0.35)***** | < 0.001 | < 0.001 |  |
| Race |  |  |  |  |  |  |  |  |  |  |  |  |  |  |
|  | Model 1 | Non-hispanic white | Ref (0.00) | -0.48 | **(-0.83, -0.13)**** | -0.62 | **(-0.91, -0.33)***** | -0.92 | **(-1.23, -0.61)***** | -1.15 | **(-1.41, -0.89)***** | < 0.001 | < 0.001 | 0.225 |
|  |  | Others | Ref (0.00) | -0.29 | (-0.61, 0.03) | -0.58 | **(-0.87, -0.29)***** | -0.56 | **(-0.89, -0.24)***** | -0.98 | **(-1.26, -0.70)***** | < 0.001 | < 0.001 |  |
|  | Model 2 | Non-hispanic white | Ref (0.00) | -0.15 | (-0.45, 0.14) | -0.25 | (-0.50, 0.00) | -0.45 | **(-0.71, -0.19)***** | -0.50 | **(-0.72, -0.29)***** | < 0.001 | < 0.001 | 0.253 |
|  |  | Others | Ref (0.00) | -0.13 | (-0.42, 0.16) | -0.38 | **(-0.65, -0.11)**** | -0.28 | (-0.59, 0.03) | -0.56 | **(-0.83, -0.30)***** | < 0.001 | < 0.001 |  |
|  | Model 3 | Non-hispanic white | Ref (0.00) | -0.04 | (-0.33, 0.25) | -0.15 | (-0.42, 0.11) | -0.32 | **(-0.58, -0.05)*** | -0.29 | **(-0.53, -0.05)*** | 0.008 | 0.020 | 0.383 |
|  |  | Others | Ref (0.00) | -0.10 | (-0.39, 0.20) | -0.37 | **(-0.64, -0.11)**** | -0.26 | (-0.56, 0.04) | -0.52 | **(-0.78, -0.26)***** | < 0.001 | < 0.001 |  |
|  | Model 4 | Non-hispanic white | Ref (0.00) | -0.14 | (-0.45, 0.18) | -0.28 | **(-0.55, -0.01)*** | -0.47 | **(-0.76, -0.17)**** | -0.49 | **(-0.77, -0.21)***** | < 0.001 | 0.001 | 0.328 |
|  |  | Others | Ref (0.00) | -0.09 | (-0.40, 0.22) | -0.37 | **(-0.65, -0.09)**** | -0.25 | (-0.59, 0.08) | -0.51 | **(-0.82, -0.20)**** | 0.001 | 0.002 |  |
| BMI |  |  |  |  |  |  |  |  |  |  |  |  |  |  |
|  | Model 1 | < 30 kg/m^2^ | Ref (0.00) | -0.52 | **(-0.82, -0.22)***** | -0.53 | **(-0.79, -0.26)***** | -0.70 | **(-0.95, -0.45)***** | -0.89 | **(-1.14, -0.65)***** | < 0.001 | < 0.001 | 0.661 |
|  |  | ≥ 30 kg/m^2^ | Ref (0.00) | 0.13 | (-0.29, 0.55) | -0.44 | **(-0.76, -0.12)**** | -0.55 | **(-0.91, -0.18)**** | -0.69 | **(-1.00, -0.38)***** | < 0.001 | < 0.001 |  |
|  | Model 2 | < 30 kg/m^2^ | Ref (0.00) | -0.39 | **(-0.69, -0.10)**** | -0.35 | **(-0.60, -0.09)**** | -0.47 | **(-0.71, -0.23)***** | -0.62 | **(-0.87, -0.38)***** | 0.003 | < 0.001 | 0.685 |
|  |  | ≥ 30 kg/m^2^ | Ref (0.00) | 0.20 | (-0.20, 0.61) | -0.34 | **(-0.65, -0.02)*** | -0.45 | **(-0.80, -0.09)*** | -0.48 | **(-0.78, -0.18)**** | < 0.001 | < 0.001 |  |
|  | Model 3 | < 30 kg/m^2^ | Ref (0.00) | -0.28 | (-0.57, 0.02) | -0.25 | (-0.51, 0.01) | -0.34 | **(-0.58, -0.10)**** | -0.43 | **(-0.68, -0.17)***** | < 0.001 | 0.001 | 0.430 |
|  |  | ≥ 30 kg/m^2^ | Ref (0.00) | 0.24 | (-0.17, 0.64) | -0.32 | **(-0.64, -0.01)*** | -0.41 | **(-0.73, -0.08)*** | -0.41 | **(-0.73, -0.09)*** | < 0.001 | < 0.001 |  |
|  | Model 4 | < 30 kg/m^2^ | Ref (0.00) | -0.34 | **(-0.64, -0.03)*** | -0.33 | **(-0.58, -0.07)*** | -0.43 | **(-0.69, -0.17)***** | -0.55 | **(-0.81, -0.28)***** | < 0.001 | < 0.001 | 0.380 |
|  |  | ≥ 30 kg/m^2^ | Ref (0.00) | 0.19 | (-0.25, 0.62) | -0.39 | **(-0.73, -0.05)*** | -0.49 | **(-0.86, -0.12)**** | -0.52 | **(-0.91, -0.13)**** | < 0.001 | < 0.001 |  |
| Smoking |  |  |  |  |  |  |  |  |  |  |  |  |  |  |
|  | Model 1 | Yes | Ref (0.00) | -0.26 | (-0.58, 0.05) | -0.38 | **(-0.74, -0.02)*** | -0.70 | **(-1.04, -0.37)***** | -1.05 | **(-1.37, -0.73)***** | < 0.001 | < 0.001 | 0.337 |
|  |  | No | Ref (0.00) | -0.50 | **(-0.90, -0.11)*** | -0.73 | **(-1.02, -0.45)***** | -0.89 | **(-1.18, -0.61)***** | -1.11 | **(-1.36, -0.86)***** | < 0.001 | < 0.001 |  |
|  | Model 2 | Yes | Ref (0.00) | -0.04 | (-0.32, 0.25) | -0.11 | (-0.44, 0.21) | -0.33 | **(-0.64, -0.03)*** | -0.50 | **(-0.80, -0.21)***** | < 0.001 | 0.001 | 0.738 |
|  |  | No | Ref (0.00) | -0.21 | (-0.55, 0.14) | -0.37 | **(-0.64, -0.11)**** | -0.46 | **(-0.72, -0.19)***** | -0.49 | **(-0.71, -0.27)***** | < 0.001 | < 0.001 |  |
|  | Model 3 | Yes | Ref (0.00) | 0.05 | (-0.25, 0.35) | -0.05 | (-0.40, 0.29) | -0.21 | (-0.51, 0.10) | -0.33 | (-0.68, 0.02) | 0.020 | 0.067 | 0.852 |
|  |  | No | Ref (0.00) | -0.13 | (-0.47, 0.21) | -0.29 | **(-0.56, -0.03)*** | -0.37 | **(-0.64, -0.10)**** | -0.33 | **(-0.58, -0.08)*** | 0.012 | 0.011 |  |
|  | Model 4 | Yes | Ref (0.00) | -0.03 | (-0.36, 0.30) | -0.15 | (-0.50, 0.20) | -0.32 | (-0.66, 0.02) | -0.47 | **(-0.87, -0.08)*** | 0.003 | 0.019 | 0.889 |
|  |  | No | Ref (0.00) | -0.18 | (-0.51, 0.16) | -0.37 | **(-0.64, -0.09)**** | -0.46 | **(-0.75, -0.16)**** | -0.44 | **(-0.74, -0.15)**** | 0.010 | 0.004 |  |
| Drinking |  |  |  |  |  |  |  |  |  |  |  |  |  |  |
|  | Model 1 | Yes | Ref (0.00) | -0.35 | **(-0.66, -0.04)*** | -0.59 | **(-0.86, -0.32)***** | -0.87 | **(-1.16, -0.58)***** | -1.09 | **(-1.34, -0.85)***** | < 0.001 | < 0.001 | 0.354 |
|  |  | No | Ref (0.00) | -0.69 | **(-1.25, -0.13)*** | -0.53 | **(-0.92, -0.13)**** | -0.77 | **(-1.23, -0.31)***** | -1.11 | **(-1.52, -0.70)***** | < 0.001 | < 0.001 |  |
|  | Model 2 | Yes | Ref (0.00) | -0.04 | (-0.31, 0.23) | -0.25 | **(-0.47, -0.02)*** | -0.45 | **(-0.70, -0.20)***** | -0.47 | **(-0.69, -0.25)***** | < 0.001 | < 0.001 | 0.366 |
|  |  | No | Ref (0.00) | -0.46 | (-0.98, 0.06) | -0.22 | (-0.60, 0.17) | -0.36 | (-0.82, 0.10) | -0.56 | **(-0.96, -0.17)**** | 0.011 | 0.006 |  |
|  | Model 3 | Yes | Ref (0.00) | 0.04 | (-0.24, 0.31) | -0.20 | (-0.43, 0.04) | -0.36 | **(-0.62, -0.09)**** | -0.34 | **(-0.59, -0.09)**** | 0.001 | 0.008 | 0.276 |
|  |  | No | Ref (0.00) | -0.40 | (-0.91, 0.12) | -0.11 | (-0.51, 0.29) | -0.26 | (-0.71, 0.20) | -0.32 | (-0.78, 0.14) | 0.273 | 0.171 |  |
|  | Model 4 | Yes | Ref (0.00) | -0.01 | (-0.31, 0.28) | -0.27 | **(-0.50, -0.04)*** | -0.44 | **(-0.73, -0.15)**** | -0.45 | **(-0.72, -0.17)**** | < 0.001 | 0.002 | 0.309 |
|  |  | No | Ref (0.00) | -0.49 | (-1.04, 0.06) | -0.24 | (-0.67, 0.19) | -0.41 | (-0.93, 0.10) | -0.52 | **(-1.01, -0.02)*** | 0.066 | 0.040 |  |
| Exercise |  |  |  |  |  |  |  |  |  |  |  |  |  |  |
|  | Model 1 | Yes | Ref (0.00) | -0.41 | (-0.85, 0.02) | -0.74 | **(-1.10, -0.39)***** | -0.73 | **(-1.11, -0.35)***** | -0.90 | **(-1.23, -0.58)***** | < 0.001 | < 0.001 | 0.587 |
|  |  | No | Ref (0.00) | -0.36 | **(-0.67, -0.05)*** | -0.42 | **(-0.73, -0.11)**** | -0.81 | **(-1.10, -0.51)***** | -1.08 | **(-1.34, -0.83)***** | < 0.001 | < 0.001 |  |
|  | Model 2 | Yes | Ref (0.00) | -0.17 | (-0.56, 0.22) | -0.39 | **(-0.69, -0.08)*** | -0.30 | (-0.61, 0.02) | -0.33 | **(-0.61, -0.04)*** | 0.063 | 0.027 | 0.251 |
|  |  | No | Ref (0.00) | -0.10 | (-0.36, 0.16) | -0.18 | (-0.45, 0.09) | -0.45 | **(-0.71, -0.19)***** | -0.59 | **(-0.81, -0.37)***** | < 0.001 | < 0.001 |  |
|  | Model 3 | Yes | Ref (0.00) | -0.20 | (-0.58, 0.18) | -0.39 | **(-0.70, -0.08)*** | -0.28 | (-0.58, 0.02) | -0.31 | **(-0.60, -0.01)*** | 0.116 | 0.043 | 0.234 |
|  |  | No | Ref (0.00) | 0.05 | (-0.21, 0.30) | -0.08 | (-0.34, 0.18) | -0.30 | **(-0.56, -0.04)*** | -0.36 | **(-0.59, -0.12)**** | < 0.001 | 0.004 |  |
|  | Model 4 | Yes | Ref (0.00) | -0.24 | (-0.66, 0.18) | -0.44 | **(-0.77, -0.10)*** | -0.34 | (-0.68, 0.00) | -0.38 | **(-0.74, -0.03)*** | 0.084 | 0.032 | 0.209 |
|  |  | No | Ref (0.00) | -0.04 | (-0.30, 0.23) | -0.20 | (-0.47, 0.07) | -0.44 | **(-0.73, -0.15)**** | -0.53 | **(-0.81, -0.25)***** | < 0.001 | < 0.001 |  |
| Education |  |  |  |  |  |  |  |  |  |  |  |  |  |  |
|  | Model 1 | Above high school | Ref (0.00) | -0.41 | **(-0.78, -0.04)*** | -0.47 | **(-0.76, -0.17)**** | -0.72 | **(-1.01, -0.42)***** | -0.98 | **(-1.23, -0.73)***** | < 0.001 | < 0.001 | 0.975 |
|  |  | Others | Ref (0.00) | -0.21 | (-0.60, 0.17) | -0.53 | **(-0.86, -0.20)**** | -0.60 | **(-0.92, -0.28)***** | -0.86 | **(-1.17, -0.55)***** | < 0.001 | < 0.001 |  |
|  | Model 2 | Above high school | Ref (0.00) | -0.16 | (-0.48, 0.15) | -0.22 | (-0.49, 0.04) | -0.34 | **(-0.62, -0.06)*** | -0.47 | **(-0.70, -0.24)***** | < 0.001 | < 0.001 | 0.787 |
|  |  | Others | Ref (0.00) | -0.08 | (-0.44, 0.27) | -0.34 | **(-0.64, -0.04)*** | -0.41 | **(-0.70, -0.12)**** | -0.49 | **(-0.78, -0.20)***** | < 0.001 | 0.001 |  |
|  | Model 3 | Above high school | Ref (0.00) | -0.07 | (-0.37, 0.23) | -0.14 | (-0.40, 0.12) | -0.24 | (-0.51, 0.03) | -0.29 | **(-0.51, -0.06)*** | 0.014 | 0.014 | 0.638 |
|  |  | Others | Ref (0.00) | 0.00 | (-0.36, 0.36) | -0.28 | (-0.60, 0.05) | -0.29 | (-0.60, 0.01) | -0.34 | (-0.68, 0.00) | 0.020 | 0.051 |  |
|  | Model 4 | Above high school | Ref (0.00) | -0.15 | (-0.45, 0.16) | -0.24 | (-0.49, 0.00) | -0.37 | **(-0.65, -0.09)*** | -0.45 | **(-0.68, -0.21)***** | 0.001 | < 0.001 | 0.523 |
|  |  | Others | Ref (0.00) | -0.05 | (-0.43, 0.34) | -0.34 | **(-0.67, -0.01)*** | -0.37 | **(-0.71, -0.03)*** | -0.44 | **(-0.78, -0.09)*** | 0.003 | 0.014 |  |
| Income |  |  |  |  |  |  |  |  |  |  |  |  |  |  |
|  | Model 1 | ≤ $55,000 | Ref (0.00) | -0.33 | (-0.66, 0.00) | -0.56 | **(-0.84, -0.28)***** | -0.71 | **(-1.04, -0.38)***** | -1.07 | **(-1.33, -0.80)***** | < 0.001 | < 0.001 | 0.504 |
|  |  | > $55,000 | Ref (0.00) | -0.41 | (-0.85, 0.02) | -0.46 | **(-0.82, -0.10)*** | -0.82 | **(-1.20, -0.44)***** | -0.88 | **(-1.19, -0.56)***** | < 0.001 | < 0.001 |  |
|  | Model 2 | ≤ $55,000 | Ref (0.00) | -0.07 | (-0.37, 0.23) | -0.25 | (-0.51, 0.00) | -0.35 | **(-0.64, -0.06)*** | -0.50 | **(-0.74, -0.26)***** | < 0.001 | < 0.001 | 0.933 |
|  |  | > $55,000 | Ref (0.00) | -0.21 | (-0.57, 0.16) | -0.23 | (-0.56, 0.10) | -0.46 | **(-0.82, -0.10)*** | -0.43 | **(-0.72, -0.14)**** | 0.003 | 0.004 |  |
|  | Model 3 | ≤ $55,000 | Ref (0.00) | 0.01 | (-0.30, 0.31) | -0.21 | (-0.47, 0.06) | -0.27 | (-0.56, 0.03) | -0.36 | **(-0.63, -0.09)**** | 0.003 | 0.009 | 0.985 |
|  |  | > $55,000 | Ref (0.00) | -0.11 | (-0.48, 0.26) | -0.12 | (-0.46, 0.21) | -0.32 | (-0.67, 0.02) | -0.24 | (-0.55, 0.07) | 0.086 | 0.120 |  |
|  | Model 4 | ≤ $55,000 | Ref (0.00) | -0.06 | (-0.37, 0.26) | -0.29 | **(-0.56, -0.01)*** | -0.37 | **(-0.68, -0.06)*** | -0.49 | **(-0.78, -0.20)***** | < 0.001 | 0.001 | 0.947 |
|  |  | > $55,000 | Ref (0.00) | -0.19 | (-0.57, 0.19) | -0.23 | (-0.55, 0.09) | -0.45 | **(-0.82, -0.07)*** | -0.40 | **(-0.74, -0.06)*** | 0.017 | 0.021 |  |
| Sleep duration |  |  |  |  |  |  |  |  |  |  |  |  |  |  |
|  | Model 1 | ≤ 7 hours | Ref (0.00) | -0.37 | (-0.74, 0.01) | -0.56 | **(-0.93, -0.20)**** | -0.60 | **(-0.93, -0.27)***** | -1.01 | **(-1.30, -0.71)***** | < 0.001 | < 0.001 | 0.046 |
|  |  | > 7 hours | Ref (0.00) | -0.44 | **(-0.79, -0.09)*** | -0.64 | **(-0.96, -0.33)***** | -0.98 | **(-1.30, -0.66)***** | -1.17 | **(-1.45, -0.89)***** | < 0.001 | < 0.001 |  |
|  | Model 2 | ≤ 7 hours | Ref (0.00) | -0.14 | (-0.47, 0.19) | -0.27 | (-0.56, 0.03) | -0.20 | (-0.51, 0.10) | -0.44 | **(-0.70, -0.18)***** | 0.001 | 0.001 | 0.090 |
|  |  | > 7 hours | Ref (0.00) | -0.14 | (-0.45, 0.17) | -0.29 | (-0.60, 0.02) | -0.53 | **(-0.82, -0.24)***** | -0.54 | **(-0.80, -0.28)***** | < 0.001 | < 0.001 |  |
|  | Model 3 | ≤ 7 hours | Ref (0.00) | -0.05 | (-0.39, 0.28) | -0.23 | (-0.54, 0.08) | -0.16 | (-0.46, 0.14) | -0.34 | **(-0.63, -0.04)*** | 0.017 | 0.026 | 0.102 |
|  |  | > 7 hours | Ref (0.00) | -0.06 | (-0.37, 0.25) | -0.20 | (-0.52, 0.11) | -0.38 | **(-0.68, -0.09)*** | -0.34 | **(-0.62, -0.05)*** | 0.007 | 0.020 |  |
|  | Model 4 | ≤ 7 hours | Ref (0.00) | -0.10 | (-0.45, 0.25) | -0.29 | (-0.59, 0.01) | -0.24 | (-0.58, 0.10) | -0.43 | **(-0.76, -0.11)**** | 0.006 | 0.009 | 0.105 |
|  |  | > 7 hours | Ref (0.00) | -0.14 | (-0.46, 0.19) | -0.31 | (-0.64, 0.02) | -0.51 | **(-0.83, -0.19)**** | -0.50 | **(-0.82, -0.18)**** | 0.001 | 0.003 |  |
| Daily energy intake |  |  |  |  |  |  |  |  |  |  |  |  |  |  |
|  | Model 1 | Higher | Ref (0.00) | -0.25 | (-0.79, 0.28) | -0.21 | (-0.66, 0.24) | -0.51 | **(-0.95, -0.06)*** | -0.82 | **(-1.26, -0.39)***** | < 0.001 | < 0.001 | < 0.001 |
|  |  | Lower | Ref (0.00) | -0.46 | **(-0.76, -0.17)**** | -0.78 | **(-1.06, -0.51)***** | -0.97 | **(-1.27, -0.68)***** | -1.28 | **(-1.52, -1.04)***** | < 0.001 | < 0.001 |  |
|  | Model 2 | Higher | Ref (0.00) | -0.02 | (-0.50, 0.45) | 0.10 | (-0.32, 0.52) | -0.09 | (-0.51, 0.33) | -0.16 | (-0.57, 0.26) | 0.202 | 0.453 | 0.015 |
|  |  | Lower | Ref (0.00) | -0.17 | (-0.43, 0.10) | -0.42 | **(-0.65, -0.19)***** | -0.50 | **(-0.77, -0.22)***** | -0.64 | **(-0.87, -0.41)***** | < 0.001 | < 0.001 |  |
|  | Model 3 | Higher | Ref (0.00) | 0.06 | (-0.42, 0.53) | 0.18 | (-0.26, 0.61) | 0.02 | (-0.40, 0.44) | -0.01 | (-0.44, 0.42) | 0.588 | 0.969 | 0.014 |
|  |  | Lower | Ref (0.00) | -0.08 | (-0.34, 0.19) | -0.35 | **(-0.58, -0.12)**** | -0.41 | **(-0.67, -0.14)**** | -0.50 | **(-0.72, -0.27)***** | < 0.001 | < 0.001 |  |
|  | Model 4 | Higher | Ref (0.00) | -0.04 | (-0.52, 0.44) | 0.04 | (-0.41, 0.49) | -0.16 | (-0.59, 0.28) | -0.24 | (-0.70, 0.22) | 0.116 | 0.299 | 0.004 |
|  |  | Lower | Ref (0.00) | -0.11 | (-0.40, 0.18) | -0.40 | **(-0.65, -0.15)**** | -0.46 | **(-0.78, -0.14)**** | -0.56 | **(-0.83, -0.30)***** | < 0.001 | < 0.001 |  |
| Healthy eating index-2015 |  |  |  |  |  |  |  |  |  |  |  |  |  |  |
|  | Model 1 | < 30.53 | Ref (0.00) | -0.56 | **(-0.90, -0.23)***** | -0.69 | **(-0.98, -0.40)***** | -0.69 | **(-1.03, -0.36)***** | -0.94 | **(-1.26, -0.62)***** | < 0.001 | < 0.001 | 0.948 |
|  |  | ≥ 30.53 | Ref (0.00) | -0.19 | (-0.58, 0.20) | -0.42 | **(-0.76, -0.07)*** | -0.80 | **(-1.14, -0.45)***** | -0.99 | **(-1.28, -0.70)***** | < 0.001 | < 0.001 |  |
|  | Model 2 | < 30.53 | Ref (0.00) | -0.23 | (-0.53, 0.07) | -0.36 | **(-0.61, -0.10)**** | -0.29 | (-0.59, 0.02) | -0.39 | **(-0.67, -0.11)**** | 0.015 | 0.007 | 0.782 |
|  |  | ≥ 30.53 | Ref (0.00) | -0.03 | (-0.39, 0.34) | -0.15 | (-0.47, 0.16) | -0.42 | **(-0.74, -0.11)**** | -0.44 | **(-0.71, -0.18)***** | < 0.001 | 0.001 |  |
|  | Model 3 | < 30.53 | Ref (0.00) | -0.19 | (-0.48, 0.10) | -0.36 | **(-0.62, -0.10)**** | -0.28 | (-0.58, 0.03) | -0.39 | **(-0.69, -0.08)*** | 0.023 | 0.014 | 0.926 |
|  |  | ≥ 30.53 | Ref (0.00) | 0.03 | (-0.35, 0.42) | -0.09 | (-0.41, 0.24) | -0.34 | **(-0.68, -0.01)*** | -0.33 | **(-0.63, -0.03)*** | 0.002 | 0.032 |  |
|  | Model 4 | < 30.53 | Ref (0.00) | -0.24 | (-0.55, 0.08) | -0.42 | **(-0.70, -0.15)**** | -0.35 | **(-0.68, -0.03)*** | -0.48 | **(-0.83, -0.14)**** | 0.011 | 0.006 | 0.968 |
|  |  | ≥ 30.53 | Ref (0.00) | -0.05 | (-0.44, 0.35) | -0.19 | (-0.51, 0.13) | -0.47 | **(-0.84, -0.10)*** | -0.49 | **(-0.81, -0.17)**** | < 0.001 | 0.003 |  |
| Dietary supplements use |  |  |  |  |  |  |  |  |  |  |  |  |  |  |
|  | Model 1 | Yes | Ref (0.00) | -0.53 | **(-0.92, -0.13)*** | -0.57 | **(-0.88, -0.26)***** | -0.94 | **(-1.26, -0.63)***** | -1.06 | **(-1.36, -0.76)***** | < 0.001 | < 0.001 | 0.515 |
|  |  | No | Ref (0.00) | -0.22 | (-0.55, 0.11) | -0.59 | **(-0.92, -0.26)***** | -0.52 | **(-0.86, -0.18)**** | -0.97 | **(-1.25, -0.70)***** | < 0.001 | < 0.001 |  |
|  | Model 2 | Yes | Ref (0.00) | -0.31 | (-0.68, 0.07) | -0.28 | (-0.57, 0.02) | -0.57 | **(-0.87, -0.27)***** | -0.54 | **(-0.81, -0.28)***** | < 0.001 | < 0.001 | 0.296 |
|  |  | No | Ref (0.00) | 0.06 | (-0.23, 0.35) | -0.29 | **(-0.56, -0.01)*** | -0.12 | (-0.40, 0.16) | -0.40 | **(-0.64, -0.16)***** | < 0.001 | 0.001 |  |
|  | Model 3 | Yes | Ref (0.00) | -0.18 | (-0.55, 0.19) | -0.17 | (-0.48, 0.13) | -0.43 | **(-0.74, -0.13)**** | -0.34 | **(-0.64, -0.04)*** | 0.032 | 0.028 | 0.502 |
|  |  | No | Ref (0.00) | 0.07 | (-0.22, 0.36) | -0.28 | (-0.57, 0.01) | -0.12 | (-0.41, 0.18) | -0.36 | **(-0.63, -0.10)**** | 0.001 | 0.008 |  |
|  | Model 4 | Yes | Ref (0.00) | -0.27 | (-0.66, 0.13) | -0.29 | (-0.60, 0.02) | -0.57 | **(-0.91, -0.24)***** | -0.52 | **(-0.86, -0.17)**** | 0.004 | 0.004 | 0.616 |
|  |  | No | Ref (0.00) | 0.03 | (-0.26, 0.32) | -0.34 | **(-0.63, -0.05)*** | -0.19 | (-0.51, 0.14) | -0.45 | **(-0.73, -0.17)**** | < 0.001 | 0.002 |  |
| Nighttime fasting duration |  |  |  |  |  |  |  |  |  |  |  |  |  |  |
|  | Model 1 | < 12.58 hours | Ref (0.00) | -0.61 | **(-1.11, -0.11)*** | -0.52 | (-1.03, 0.00) | -0.80 | **(-1.28, -0.31)**** | -1.11 | **(-1.55, -0.67)***** | < 0.001 | < 0.001 | < 0.001 |
|  |  | ≥ 12.588 hours | Ref (0.00) | -0.28 | (-0.61, 0.04) | -0.68 | **(-0.97, -0.38)***** | -0.79 | **(-1.12, -0.46)***** | -0.91 | **(-1.19, -0.64)***** | < 0.001 | < 0.001 |  |
|  | Model 2 | < 12.58 hours | Ref (0.00) | -0.34 | (-0.77, 0.09) | -0.18 | (-0.64, 0.27) | -0.44 | (-0.87, 0.00) | -0.54 | **(-0.94, -0.14)**** | 0.002 | 0.009 | 0.001 |
|  |  | ≥ 12.588 hours | Ref (0.00) | -0.04 | (-0.33, 0.26) | -0.40 | **(-0.66, -0.14)**** | -0.33 | **(-0.63, -0.02)*** | -0.39 | **(-0.65, -0.12)**** | 0.001 | 0.006 |  |
|  | Model 3 | < 12.58 hours | Ref (0.00) | -0.28 | (-0.73, 0.16) | -0.15 | (-0.60, 0.30) | -0.36 | (-0.80, 0.07) | -0.44 | **(-0.84, -0.04)*** | 0.011 | 0.033 | 0.001 |
|  |  | ≥ 12.588 hours | Ref (0.00) | 0.08 | (-0.21, 0.37) | -0.31 | **(-0.58, -0.04)*** | -0.20 | (-0.50, 0.10) | -0.18 | (-0.46, 0.11) | 0.048 | 0.222 |  |

^a^The variables adjusted in each model were the factors mentioned above except the stratification variables.

^b^Data were listed as the weighted beta estimates and 95% confidence intervals, with *p < 0.05, **p < 0.01, ***p < 0.001.

^c^Q, quintile.

^d^Ref, reference.

^e^Tests for trends based on the variables containing the median values for each quartile.

^f^*P*_test_ was the result of Bonfreni correction.

^g^Multiplicative interaction was assessed by adding interaction terms to the models.

**Supplementary table 9.** Association of DEF with AL stratified by variables of interest

|  |  |  | Daily eating frequency (DEF) | | | | | | | | |  |  |  |
| --- | --- | --- | --- | --- | --- | --- | --- | --- | --- | --- | --- | --- | --- | --- |
|  |  |  | Q1^c^ | Q2 | | Q3 | | Q4 | | Q5 | | *P*_trend_^e^ | *P*_test_^f^ | *P*_interaction_^g^ |
|  |  |  | β^b^ | β | 95% CI | β | 95% CI | β | 95% CI | β | 95% CI |  |  |  |
| Age |  |  |  |  |  |  |  |  |  |  |  |  |  |  |
|  | Model 1^a^ | > 60 years | Ref (0.00)^d^ | -0.01 | (-0.03, 0.01) | -0.02 | (-0.03, 0.00) | -0.02 | (-0.04, 0.00) | -0.04 | **(-0.05, -0.03)***** | < 0.001 | < 0.001 | 0.160 |
|  |  | ≤ 60 years | Ref (0.00) | -0.01 | (-0.02, 0.00) | -0.01 | **(-0.02, -0.01)**** | -0.02 | **(-0.03, -0.01)**** | -0.02 | **(-0.03, -0.01)***** | < 0.001 | < 0.001 |  |
|  | Model 2 | > 60 years | Ref (0.00) | 0.00 | (-0.02, 0.01) | -0.01 | (-0.02, 0.01) | -0.01 | (-0.03, 0.00) | -0.03 | **(-0.04, -0.01)***** | < 0.001 | 0.001 | 0.016 |
|  |  | ≤ 60 years | Ref (0.00) | 0.00 | (-0.01, 0.01) | -0.01 | (-0.01, 0.00) | 0.00 | (-0.01, 0.01) | 0.00 | (-0.01, 0.01) | 0.716 | < 0.001 |  |
|  | Model 3 | > 60 years | Ref (0.00) | 0.00 | (-0.02, 0.01) | -0.01 | (-0.02, 0.01) | -0.01 | (-0.02, 0.01) | -0.02 | (-0.03, 0.00) | 0.028 | 0.040 | 0.010 |
|  |  | ≤ 60 years | Ref (0.00) | 0.00 | (-0.01, 0.01) | 0.00 | (-0.01, 0.01) | 0.00 | (-0.01, 0.01) | 0.00 | (-0.01, 0.01) | 0.815 | < 0.001 |  |
|  | Model 4 | > 60 years | Ref (0.00) | 0.00 | (-0.02, 0.02) | -0.01 | (-0.02, 0.01) | -0.01 | (-0.02, 0.01) | -0.01 | (-0.03, 0.00) | 0.039 | 0.077 | 0.012 |
|  |  | ≤ 60 years | Ref (0.00) | 0.00 | (-0.01, 0.01) | -0.01 | (-0.02, 0.00) | -0.01 | (-0.02, 0.00) | -0.01 | (-0.01, 0.01) | 0.393 | < 0.001 |  |
| Sex |  |  |  |  |  |  |  |  |  |  |  |  |  |  |
|  | Model 1 | Male | Ref (0.00) | -0.01 | (-0.03, 0.00) | -0.02 | **(-0.03, -0.01)**** | -0.02 | **(-0.04, -0.01)***** | -0.03 | **(-0.05, -0.02)***** | < 0.001 | < 0.001 | 0.559 |
|  |  | Female | Ref (0.00) | -0.01 | (-0.02, 0.00) | -0.03 | **(-0.04, -0.02)***** | -0.03 | **(-0.04, -0.02)***** | -0.03 | **(-0.04, -0.03)***** | < 0.001 | < 0.001 |  |
|  | Model 2 | Male | Ref (0.00) | -0.01 | (-0.02, 0.00) | -0.01 | (-0.02, 0.00) | -0.01 | (-0.03, 0.00) | -0.02 | **(-0.03, -0.01)***** | 0.001 | < 0.001 | 0.075 |
|  |  | Female | Ref (0.00) | 0.00 | (-0.01, 0.01) | -0.01 | (-0.02, 0.00) | -0.01 | (-0.02, 0.00) | -0.01 | (-0.02, 0.00) | 0.002 | 0.004 |  |
|  | Model 3 | Male | Ref (0.00) | -0.01 | (-0.02, 0.01) | -0.01 | (-0.02, 0.00) | -0.01 | (-0.02, 0.00) | -0.02 | (-0.03, 0.00) | 0.009 | 0.008 | 0.054 |
|  |  | Female | Ref (0.00) | 0.00 | (-0.01, 0.01) | -0.01 | (-0.02, 0.00) | -0.01 | (-0.02, 0.00) | -0.01 | (-0.02, 0.00) | 0.040 | 0.043 |  |
|  | Model 4 | Male | Ref (0.00) | -0.01 | (-0.02, 0.01) | -0.01 | (-0.02, 0.00) | -0.01 | (-0.03, 0.00) | -0.02 | (-0.03, 0.00) | 0.015 | 0.014 | 0.054 |
|  |  | Female | Ref (0.00) | 0.00 | (-0.01, 0.01) | -0.01 | (-0.02, 0.00) | -0.01 | (-0.02, 0.00) | -0.01 | (-0.02, 0.00) | 0.069 | 0.056 |  |
| Race |  |  |  |  |  |  |  |  |  |  |  |  |  |  |
|  | Model 1 | Non-hispanic white | Ref (0.00) | -0.01 | (-0.02, 0.00) | -0.02 | **(-0.03, -0.01)***** | -0.02 | **(-0.04, -0.01)***** | -0.03 | **(-0.04, -0.02)***** | < 0.001 | < 0.001 | 0.165 |
|  |  | Others | Ref (0.00) | -0.01 | (-0.02, 0.00) | -0.02 | **(-0.03, -0.01)***** | -0.02 | **(-0.03, -0.01)***** | -0.03 | **(-0.04, -0.02)***** | < 0.001 | < 0.001 |  |
|  | Model 2 | Non-hispanic white | Ref (0.00) | 0.00 | (-0.01, 0.01) | -0.01 | (-0.02, 0.00) | -0.01 | (-0.02, 0.00) | -0.02 | **(-0.02, -0.01)***** | 0.001 | 0.001 | 0.151 |
|  |  | Others | Ref (0.00) | -0.01 | (-0.01, 0.01) | -0.01 | (-0.02, 0.00) | -0.01 | (-0.02, 0.00) | -0.01 | **(-0.02, -0.01)***** | 0.001 | 0.001 |  |
|  | Model 3 | Non-hispanic white | Ref (0.00) | 0.00 | (-0.01, 0.01) | -0.01 | (-0.02, 0.00) | -0.01 | (-0.02, 0.00) | -0.01 | (-0.02, 0.00) | 0.039 | 0.043 | 0.180 |
|  |  | Others | Ref (0.00) | -0.01 | (-0.02, 0.01) | -0.01 | (-0.02, 0.00) | -0.01 | (-0.02, 0.00) | -0.02 | **(-0.03, -0.01)***** | < 0.001 | 0.001 |  |
|  | Model 4 | Non-hispanic white | Ref (0.00) | 0.00 | (-0.01, 0.01) | -0.01 | (-0.02, 0.00) | -0.01 | (-0.02, 0.00) | -0.01 | (-0.02, 0.00) | 0.058 | 0.064 | 0.172 |
|  |  | Others | Ref (0.00) | -0.01 | (-0.02, 0.00) | -0.01 | (-0.02, 0.00) | -0.01 | (-0.02, 0.00) | -0.02 | **(-0.03, -0.01)***** | 0.001 | 0.001 |  |
| BMI |  |  |  |  |  |  |  |  |  |  |  |  |  |  |
|  | Model 1 | < 30 kg/m^2^ | Ref (0.00) | -0.01 | (-0.02, 0.00) | -0.02 | **(-0.03, -0.01)***** | -0.02 | **(-0.03, -0.01)***** | -0.03 | **(-0.03, -0.02)***** | < 0.001 | < 0.001 | 0.794 |
|  |  | ≥ 30 kg/m^2^ | Ref (0.00) | 0.00 | (-0.01, 0.01) | -0.01 | (-0.03, 0.00) | -0.01 | (-0.03, 0.00) | -0.02 | **(-0.03, -0.01)***** | < 0.001 | < 0.001 |  |
|  | Model 2 | < 30 kg/m^2^ | Ref (0.00) | -0.01 | (-0.02, 0.00) | -0.02 | **(-0.02, -0.01)***** | -0.02 | **(-0.02, -0.01)***** | -0.02 | **(-0.03, -0.01)***** | < 0.001 | < 0.001 | 0.834 |
|  |  | ≥ 30 kg/m^2^ | Ref (0.00) | 0.00 | (-0.01, 0.01) | -0.01 | (-0.02, 0.00) | -0.01 | (-0.02, 0.00) | -0.02 | **(-0.03, -0.01)**** | 0.001 | < 0.001 |  |
|  | Model 3 | < 30 kg/m^2^ | Ref (0.00) | -0.01 | (-0.02, 0.00) | -0.01 | (-0.02, 0.00) | -0.01 | (-0.02, 0.00) | -0.01 | **(-0.02, -0.01)**** | 0.003 | 0.002 | 0.623 |
|  |  | ≥ 30 kg/m^2^ | Ref (0.00) | 0.00 | (-0.01, 0.01) | -0.01 | (-0.02, 0.00) | -0.01 | (-0.02, 0.00) | -0.02 | (-0.03, 0.00) | 0.002 | < 0.001 |  |
|  | Model 4 | < 30 kg/m^2^ | Ref (0.00) | -0.01 | (-0.02, 0.00) | -0.01 | (-0.02, 0.00) | -0.01 | (-0.02, 0.00) | -0.01 | (-0.02, 0.00) | 0.011 | 0.005 | 0.597 |
|  |  | ≥ 30 kg/m^2^ | Ref (0.00) | 0.00 | (-0.01, 0.02) | -0.01 | (-0.03, 0.00) | -0.01 | (-0.03, 0.00) | -0.02 | (-0.03, 0.00) | 0.006 | < 0.001 |  |
| Smoking |  |  |  |  |  |  |  |  |  |  |  |  |  |  |
|  | Model 1 | Yes | Ref (0.00) | -0.01 | (-0.03, 0.00) | -0.02 | **(-0.03, -0.01)***** | -0.02 | **(-0.03, -0.01)***** | -0.03 | **(-0.04, -0.02)***** | < 0.001 | < 0.001 | 0.103 |
|  |  | No | Ref (0.00) | -0.01 | (-0.02, 0.00) | -0.02 | **(-0.03, -0.01)***** | -0.02 | **(-0.04, -0.01)***** | -0.03 | **(-0.04, -0.02)***** | < 0.001 | < 0.001 |  |
|  | Model 2 | Yes | Ref (0.00) | -0.01 | (-0.02, 0.00) | -0.01 | (-0.02, 0.00) | -0.01 | (-0.02, 0.00) | -0.02 | **(-0.03, -0.01)**** | 0.005 | 0.003 | 0.337 |
|  |  | No | Ref (0.00) | 0.00 | (-0.01, 0.01) | -0.01 | (-0.02, 0.00) | -0.01 | (-0.02, 0.00) | -0.01 | **(-0.02, -0.01)***** | 0.001 | 0.001 |  |
|  | Model 3 | Yes | Ref (0.00) | -0.01 | (-0.02, 0.01) | -0.01 | (-0.02, 0.00) | -0.01 | (-0.02, 0.00) | -0.01 | (-0.02, 0.00) | 0.064 | 0.045 | 0.515 |
|  |  | No | Ref (0.00) | 0.00 | (-0.01, 0.01) | -0.01 | (-0.02, 0.00) | -0.01 | (-0.02, 0.00) | -0.01 | (-0.02, 0.00) | 0.009 | 0.022 |  |
|  | Model 4 | Yes | Ref (0.00) | -0.01 | (-0.02, 0.01) | -0.01 | (-0.02, 0.00) | -0.01 | (-0.02, 0.00) | -0.01 | (-0.03, 0.00) | 0.087 | 0.048 | 0.501 |
|  |  | No | Ref (0.00) | 0.00 | (-0.01, 0.01) | -0.01 | (-0.02, 0.00) | -0.01 | (-0.02, 0.00) | -0.01 | (-0.02, 0.00) | 0.014 | 0.027 |  |
| Drinking |  |  |  |  |  |  |  |  |  |  |  |  |  |  |
|  | Model 1 | Yes | Ref (0.00) | -0.01 | (-0.02, 0.00) | -0.02 | **(-0.03, -0.01)***** | -0.02 | **(-0.04, -0.01)***** | -0.03 | **(-0.04, -0.02)***** | < 0.001 | < 0.001 | 0.943 |
|  |  | No | Ref (0.00) | -0.01 | (-0.03, 0.00) | -0.01 | (-0.02, 0.01) | -0.03 | **(-0.04, -0.01)***** | -0.03 | **(-0.05, -0.02)***** | < 0.001 | < 0.001 |  |
|  | Model 2 | Yes | Ref (0.00) | 0.00 | (-0.01, 0.01) | -0.01 | **(-0.02, -0.01)**** | -0.01 | (-0.02, 0.00) | -0.02 | **(-0.02, -0.01)***** | 0.001 | 0.001 | 0.860 |
|  |  | No | Ref (0.00) | -0.01 | (-0.02, 0.01) | 0.00 | (-0.01, 0.02) | -0.01 | (-0.03, 0.00) | -0.01 | (-0.03, 0.00) | 0.017 | 0.036 |  |
|  | Model 3 | Yes | Ref (0.00) | 0.00 | (-0.01, 0.01) | -0.01 | (-0.02, 0.00) | -0.01 | (-0.02, 0.00) | -0.01 | (-0.02, 0.00) | 0.012 | 0.015 | 0.831 |
|  |  | No | Ref (0.00) | -0.01 | (-0.02, 0.01) | 0.00 | (-0.01, 0.02) | -0.01 | (-0.03, 0.00) | -0.01 | (-0.03, 0.00) | 0.080 | 0.129 |  |
|  | Model 4 | Yes | Ref (0.00) | 0.00 | (-0.01, 0.01) | -0.01 | (-0.02, 0.00) | -0.01 | (-0.02, 0.00) | -0.01 | (-0.02, 0.00) | 0.036 | 0.037 | 0.8544 |
|  |  | No | Ref (0.00) | -0.01 | (-0.02, 0.01) | 0.00 | (-0.01, 0.02) | -0.01 | (-0.03, 0.00) | -0.02 | (-0.03, 0.00) | 0.039 | 0.074 |  |
| Exercise |  |  |  |  |  |  |  |  |  |  |  |  |  |  |
|  | Model 1 | Yes | Ref (0.00) | -0.01 | (-0.03, 0.01) | -0.02 | **(-0.04, -0.01)***** | -0.03 | **(-0.04, -0.01)***** | -0.03 | **(-0.04, -0.02)***** | < 0.001 | < 0.001 | 0.962 |
|  |  | No | Ref (0.00) | -0.01 | (-0.02, 0.00) | -0.02 | **(-0.03, -0.01)***** | -0.02 | **(-0.03, -0.01)***** | -0.03 | **(-0.04, -0.02)***** | < 0.001 | < 0.001 |  |
|  | Model 2 | Yes | Ref (0.00) | 0.00 | (-0.02, 0.01) | -0.01 | (-0.02, 0.00) | -0.01 | (-0.02, 0.00) | -0.01 | (-0.02, 0.00) | 0.046 | 0.042 | 0.564 |
|  |  | No | Ref (0.00) | -0.01 | (-0.01, 0.00) | -0.01 | (-0.02, 0.00) | -0.01 | (-0.02, 0.00) | -0.02 | **(-0.03, -0.01)***** | < 0.001 | < 0.001 |  |
|  | Model 3 | Yes | Ref (0.00) | 0.00 | (-0.02, 0.01) | -0.01 | (-0.03, 0.00) | -0.01 | (-0.02, 0.00) | -0.01 | (-0.02, 0.00) | 0.054 | 0.042 | 0.578 |
|  |  | No | Ref (0.00) | 0.00 | (-0.01, 0.01) | -0.01 | (-0.02, 0.00) | -0.01 | (-0.02, 0.00) | -0.01 | (-0.02, 0.00) | 0.003 | 0.006 |  |
|  | Model 4 | Yes | Ref (0.00) | 0.00 | (-0.02, 0.01) | -0.01 | (-0.02, 0.00) | -0.01 | (-0.02, 0.00) | -0.01 | (-0.02, 0.00) | 0.168 | 0.127 | 0.558 |
|  |  | No | Ref (0.00) | 0.00 | (-0.01, 0.01) | -0.01 | (-0.02, 0.00) | -0.01 | (-0.02, 0.00) | -0.02 | **(-0.03, -0.01)**** | 0.002 | 0.005 |  |
| Education |  |  |  |  |  |  |  |  |  |  |  |  |  |  |
|  | Model 1 | Above high school | Ref (0.00) | -0.01 | (-0.03, 0.00) | -0.02 | **(-0.03, -0.01)***** | -0.02 | **(-0.04, -0.01)***** | -0.03 | **(-0.04, -0.02)***** | < 0.001 | < 0.001 | 0.405 |
|  |  | Others | Ref (0.00) | -0.01 | (-0.02, 0.01) | -0.02 | **(-0.03, -0.01)***** | -0.02 | **(-0.03, -0.01)**** | -0.02 | **(-0.04, -0.01)***** | < 0.001 | < 0.001 |  |
|  | Model 2 | Above high school | Ref (0.00) | -0.01 | (-0.02, 0.00) | -0.01 | (-0.02, 0.00) | -0.01 | (-0.02, 0.00) | -0.02 | **(-0.03, -0.01)***** | < 0.001 | 0.001 | 0.508 |
|  |  | Others | Ref (0.00) | 0.00 | (-0.01, 0.01) | -0.01 | (-0.02, 0.00) | -0.01 | (-0.02, 0.00) | -0.01 | (-0.02, 0.00) | 0.007 | 0.011 |  |
|  | Model 3 | Above high school | Ref (0.00) | -0.01 | (-0.02, 0.01) | -0.01 | (-0.02, 0.00) | -0.01 | (-0.02, 0.00) | -0.01 | (-0.03, 0.00) | 0.009 | 0.010 | 0.588 |
|  |  | Others | Ref (0.00) | 0.00 | (-0.01, 0.01) | -0.01 | (-0.02, 0.00) | -0.01 | (-0.02, 0.00) | -0.01 | (-0.02, 0.00) | 0.127 | 0.161 |  |
|  | Model 4 | Above high school | Ref (0.00) | -0.01 | (-0.02, 0.01) | -0.01 | (-0.02, 0.00) | -0.01 | (-0.03, 0.00) | -0.02 | (-0.03, 0.00) | 0.009 | 0.008 | 0.638 |
|  |  | Others | Ref (0.00) | 0.00 | (-0.01, 0.01) | -0.01 | (-0.02, 0.00) | -0.01 | (-0.02, 0.01) | -0.01 | (-0.02, 0.01) | 0.244 | 0.294 |  |
| Income |  |  |  |  |  |  |  |  |  |  |  |  |  |  |
|  | Model 1 | ≤ $55,000 | Ref (0.00) | -0.01 | (-0.02, 0.00) | -0.02 | **(-0.03, -0.01)***** | -0.02 | **(-0.03, -0.01)***** | -0.03 | **(-0.04, -0.02)***** | < 0.001 | < 0.001 | 0.222 |
|  |  | > $55,000 | Ref (0.00) | -0.01 | (-0.02, 0.00) | -0.02 | (-0.03, 0.00) | -0.03 | **(-0.04, -0.01)***** | -0.03 | **(-0.04, -0.02)***** | < 0.001 | < 0.001 |  |
|  | Model 2 | ≤ $55,000 | Ref (0.00) | -0.01 | (-0.02, 0.00) | -0.01 | **(-0.02, -0.01)**** | -0.01 | (-0.02, 0.00) | -0.01 | (-0.02, 0.00) | 0.007 | 0.006 | 0.438 |
|  |  | > $55,000 | Ref (0.00) | 0.00 | (-0.02, 0.01) | -0.01 | (-0.02, 0.00) | -0.02 | (-0.03, 0.00) | -0.02 | **(-0.03, -0.01)***** | 0.001 | 0.001 |  |
|  | Model 3 | ≤ $55,000 | Ref (0.00) | 0.00 | (-0.01, 0.01) | -0.01 | (-0.02, 0.00) | -0.01 | (-0.02, 0.00) | -0.01 | (-0.02, 0.00) | 0.098 | 0.078 | 0.544 |
|  |  | > $55,000 | Ref (0.00) | 0.00 | (-0.01, 0.01) | -0.01 | (-0.02, 0.01) | -0.01 | (-0.03, 0.00) | -0.01 | (-0.03, 0.00) | 0.002 | 0.008 |  |
|  | Model 4 | ≤ $55,000 | Ref (0.00) | 0.00 | (-0.01, 0.01) | -0.01 | (-0.02, 0.00) | -0.01 | (-0.02, 0.00) | -0.01 | (-0.02, 0.00) | 0.498 | 0.197 | 0.534 |
|  |  | > $55,000 | Ref (0.00) | -0.01 | (-0.02, 0.01) | -0.01 | (-0.02, 0.00) | -0.02 | (-0.03, 0.00) | -0.02 | **(-0.03, -0.01)**** | 0.001 | 0.004 |  |
| Sleep duration |  |  |  |  |  |  |  |  |  |  |  |  |  |  |
|  | Model 1 | ≤ 7 hours | Ref (0.00) | -0.01 | (-0.03, 0.00) | -0.03 | **(-0.04, -0.01)***** | -0.02 | **(-0.04, -0.01)***** | -0.03 | **(-0.05, -0.02)***** | < 0.001 | < 0.001 | 0.277 |
|  |  | > 7 hours | Ref (0.00) | -0.01 | (-0.02, 0.00) | -0.02 | **(-0.03, -0.01)***** | -0.02 | **(-0.03, -0.01)***** | -0.03 | **(-0.04, -0.03)***** | < 0.001 | < 0.001 |  |
|  | Model 2 | ≤ 7 hours | Ref (0.00) | -0.01 | (-0.02, 0.00) | -0.02 | (-0.03, 0.00) | -0.01 | (-0.02, 0.00) | -0.02 | **(-0.03, -0.01)**** | 0.010 | 0.006 | 0.493 |
|  |  | > 7 hours | Ref (0.00) | 0.00 | (-0.01, 0.01) | -0.01 | (-0.02, 0.00) | -0.01 | (-0.02, 0.00) | -0.02 | **(-0.02, -0.01)***** | < 0.001 | 0.001 |  |
|  | Model 3 | ≤ 7 hours | Ref (0.00) | -0.01 | (-0.02, 0.00) | -0.02 | (-0.03, 0.00) | -0.01 | (-0.02, 0.00) | -0.02 | (-0.03, 0.00) | 0.028 | 0.020 | 0.577 |
|  |  | > 7 hours | Ref (0.00) | 0.00 | (-0.01, 0.01) | -0.01 | (-0.02, 0.00) | -0.01 | (-0.02, 0.00) | -0.01 | (-0.02, 0.00) | 0.022 | 0.049 |  |
|  | Model 4 | ≤ 7 hours | Ref (0.00) | -0.01 | (-0.02, 0.01) | -0.01 | (-0.03, 0.00) | -0.01 | (-0.02, 0.00) | -0.01 | (-0.03, 0.00) | 0.114 | 0.083 | 0.588 |
|  |  | > 7 hours | Ref (0.00) | 0.00 | (-0.01, 0.01) | -0.01 | (-0.02, 0.00) | -0.01 | (-0.02, 0.00) | -0.01 | (-0.03, 0.00) | 0.011 | 0.023 |  |
| Daily energy intake |  |  |  |  |  |  |  |  |  |  |  |  |  |  |
|  | Model 1 | Higher | Ref (0.00) | -0.02 | (-0.04, 0.00) | -0.02 | (-0.03, 0.00) | -0.03 | **(-0.04, -0.01)**** | -0.04 | **(-0.05, -0.02)***** | < 0.001 | < 0.001 | 0.109 |
|  |  | Lower | Ref (0.00) | -0.01 | (-0.02, 0.00) | -0.03 | **(-0.04, -0.02)***** | -0.03 | **(-0.04, -0.02)***** | -0.04 | **(-0.04, -0.03)***** | < 0.001 | < 0.001 |  |
|  | Model 2 | Higher | Ref (0.00) | -0.01 | (-0.03, 0.00) | -0.01 | (-0.02, 0.01) | -0.01 | (-0.03, 0.00) | -0.02 | (-0.03, 0.00) | 0.013 | 0.016 | 0.690 |
|  |  | Lower | Ref (0.00) | 0.00 | (-0.01, 0.01) | -0.01 | **(-0.02, -0.01)**** | -0.01 | (-0.02, 0.00) | -0.02 | **(-0.02, -0.01)***** | 0.001 | 0.001 |  |
|  | Model 3 | Higher | Ref (0.00) | -0.01 | (-0.03, 0.01) | -0.01 | (-0.02, 0.01) | -0.01 | (-0.03, 0.01) | -0.01 | (-0.03, 0.00) | 0.048 | 0.050 | 0.662 |
|  |  | Lower | Ref (0.00) | 0.00 | (-0.01, 0.01) | -0.01 | (-0.02, 0.00) | -0.01 | (-0.02, 0.00) | -0.01 | (-0.02, 0.00) | 0.003 | 0.006 |  |
|  | Model 4 | Higher | Ref (0.00) | -0.01 | (-0.03, 0.00) | -0.01 | (-0.03, 0.01) | -0.02 | (-0.03, 0.00) | -0.02 | **(-0.04, -0.01)**** | 0.006 | 0.007 | 0.552 |
|  |  | Lower | Ref (0.00) | 0.00 | (-0.01, 0.01) | -0.01 | (-0.02, 0.00) | -0.01 | (-0.02, 0.00) | -0.01 | (-0.02, 0.00) | 0.043 | 0.071 |  |
| Healthy eating index-2015 |  |  |  |  |  |  |  |  |  |  |  |  |  |  |
|  | Model 1 | < 30.53 | Ref (0.00) | -0.02 | **(-0.03, -0.01)***** | -0.02 | **(-0.03, -0.01)***** | -0.02 | **(-0.03, -0.01)***** | -0.03 | **(-0.04, -0.02)***** | < 0.001 | < 0.001 | 0.880 |
|  |  | ≥ 30.53 | Ref (0.00) | -0.01 | (-0.02, 0.01) | -0.02 | **(-0.03, -0.01)***** | -0.02 | **(-0.04, -0.01)***** | -0.03 | **(-0.04, -0.02)***** | < 0.001 | < 0.001 |  |
|  | Model 2 | < 30.53 | Ref (0.00) | -0.01 | (-0.02, 0.00) | -0.01 | (-0.02, 0.00) | -0.01 | (-0.02, 0.00) | -0.01 | (-0.02, 0.00) | 0.048 | 0.021 | 0.604 |
|  |  | ≥ 30.53 | Ref (0.00) | 0.00 | (-0.01, 0.01) | -0.01 | (-0.02, 0.00) | -0.01 | (-0.03, 0.00) | -0.02 | **(-0.03, -0.01)***** | < 0.001 | < 0.001 |  |
|  | Model 3 | < 30.53 | Ref (0.00) | -0.01 | (-0.01, 0.00) | -0.01 | (-0.02, 0.00) | -0.01 | (-0.02, 0.00) | -0.01 | (-0.02, 0.00) | 0.146 | 0.089 | 0.685 |
|  |  | ≥ 30.53 | Ref (0.00) | 0.00 | (-0.01, 0.01) | -0.01 | (-0.02, 0.00) | -0.01 | (-0.02, 0.00) | -0.02 | **(-0.02, -0.01)***** | < 0.001 | 0.001 |  |
|  | Model 4 | < 30.53 | Ref (0.00) | -0.01 | (-0.02, 0.00) | -0.01 | (-0.02, 0.00) | -0.01 | (-0.02, 0.00) | -0.01 | (-0.02, 0.00) | 0.172 | 0.098 | 0.730 |
|  |  | ≥ 30.53 | Ref (0.00) | 0.00 | (-0.01, 0.01) | -0.01 | (-0.02, 0.00) | -0.01 | (-0.03, 0.00) | -0.02 | **(-0.03, -0.01)**** | 0.002 | 0.003 |  |
| Dietary supplements use |  |  |  |  |  |  |  |  |  |  |  |  |  |  |
|  | Model 1 | Yes | Ref (0.00) | -0.01 | (-0.02, 0.01) | -0.02 | (-0.03, 0.00) | -0.02 | **(-0.04, -0.01)***** | -0.03 | **(-0.04, -0.02)***** | < 0.001 | < 0.001 | 0.552 |
|  |  | No | Ref (0.00) | -0.02 | **(-0.03, -0.01)***** | -0.03 | **(-0.04, -0.02)***** | -0.02 | **(-0.03, -0.01)***** | -0.03 | **(-0.04, -0.02)***** | < 0.001 | < 0.001 |  |
|  | Model 2 | Yes | Ref (0.00) | 0.00 | (-0.01, 0.01) | -0.01 | (-0.02, 0.00) | -0.01 | (-0.02, 0.00) | -0.01 | (-0.02, 0.00) | 0.002 | 0.010 | 0.604 |
|  |  | No | Ref (0.00) | -0.01 | (-0.02, 0.00) | -0.02 | **(-0.02, -0.01)***** | -0.01 | (-0.02, 0.00) | -0.02 | **(-0.03, -0.01)***** | 0.002 | 0.001 |  |
|  | Model 3 | Yes | Ref (0.00) | 0.00 | (-0.01, 0.02) | -0.01 | (-0.02, 0.01) | -0.01 | (-0.02, 0.00) | -0.01 | (-0.02, 0.00) | 0.016 | 0.072 | 0.461 |
|  |  | No | Ref (0.00) | -0.01 | (-0.02, 0.00) | -0.02 | **(-0.02, -0.01)**** | -0.01 | (-0.02, 0.01) | -0.01 | (-0.03, 0.00) | 0.023 | 0.008 |  |
|  | Model 4 | Yes | Ref (0.00) | 0.00 | (-0.01, 0.01) | -0.01 | (-0.02, 0.00) | -0.01 | (-0.03, 0.00) | -0.01 | (-0.03, 0.00) | 0.008 | 0.035 | 0.420 |
|  |  | No | Ref (0.00) | -0.01 | (-0.02, 0.00) | -0.01 | (-0.02, 0.00) | 0.00 | (-0.01, 0.01) | -0.01 | (-0.02, 0.00) | 0.099 | 0.031 |  |
| Nighttime fasting duration |  |  |  |  |  |  |  |  |  |  |  |  |  |  |
|  | Model 1 | < 12.58 hours | Ref (0.00) | -0.02 | (-0.03, 0.00) | -0.02 | **(-0.04, -0.01)*** | -0.03 | **(-0.04, -0.01)**** | -0.04 | **(-0.05, -0.02)***** | < 0.001 | < 0.001 | 0.001 |
|  |  | ≥ 12.58 hours | Ref (0.00) | -0.01 | (-0.02, 0.00) | -0.02 | **(-0.03, -0.01)***** | -0.02 | **(-0.03, -0.01)**** | -0.02 | **(-0.04, -0.01)***** | < 0.001 | < 0.001 |  |
|  | Model 2 | < 12.58 hours | Ref (0.00) | -0.01 | (-0.02, 0.01) | -0.01 | (-0.02, 0.00) | -0.02 | (-0.03, 0.00) | -0.02 | (-0.03, 0.00) | 0.006 | 0.011 | 0.003 |
|  |  | ≥ 12.58 hours | Ref (0.00) | 0.00 | (-0.01, 0.01) | -0.01 | (-0.02, 0.00) | 0.00 | (-0.02, 0.01) | -0.01 | (-0.02, 0.00) | 0.065 | 0.124 |  |
|  | Model 3 | < 12.58 hours | Ref (0.00) | -0.01 | (-0.02, 0.01) | -0.01 | (-0.02, 0.00) | -0.01 | (-0.03, 0.00) | -0.02 | (-0.03, 0.00) | 0.026 | 0.030 | 0.005 |
|  |  | ≥ 12.58 hours | Ref (0.00) | 0.00 | (-0.01, 0.01) | -0.01 | (-0.02, 0.00) | 0.00 | (-0.01, 0.01) | -0.01 | (-0.02, 0.01) | 0.291 | 0.435 |  |

^a^The variables adjusted in each model were the factors mentioned above except the stratification variables.

^b^Data were listed as the weighted beta estimates and 95% confidence intervals, with *p < 0.05, **p < 0.01, ***p < 0.001.

^c^Q, quintile.

^d^Ref, reference.

^e^Tests for trends based on the variables containing the median values for each quartile.

^f^*P*_test_ was the result of Bonfreni correction.

^g^Multiplicative interaction was assessed by adding interaction terms to the models.

**Supplementary table 10.** Association of NFD with Ln-transformed HD stratified by variables of interest

|  |  |  | Nighttime fasting duration (hours) | | | | | | | | |  |  |  |
| --- | --- | --- | --- | --- | --- | --- | --- | --- | --- | --- | --- | --- | --- | --- |
|  |  |  | Q1^c^ | | Q2 | Q3 | | Q4 | | Q5 | | *P*_trend_^e^ | *P*_test_^f^ | *P*_interaction_^g^ |
|  |  |  | β^b^ | 95% CI | β | β | 95% CI | β | 95% CI | β | 95% CI |  |  |  |
| Age |  |  |  |  |  |  |  |  |  |  |  |  |  |  |
|  | Model 1^a^ | > 60 years | 0.00 | (-0.04,0.04) | Ref (0.00)^d^ | 0.01 | (-0.02,0.04) | 0.03 | (0.00,0.06) | 0.08 | **(0.05,0.11)***** | <0.001 | <0.001 | 0.001 |
|  |  | ≤ 60 years | 0.02 | (0.00,0.04) | Ref (0.00) | 0.01 | (-0.01,0.03) | 0.02 | (-0.01,0.04) | 0.03 | **(0.02,0.05)***** | 0.01 | <0.001 |  |
|  | Model 2 | > 60 years | 0.01 | (-0.03,0.05) | Ref (0.00) | 0.01 | (-0.02,0.04) | 0.03 | (0.00,0.06) | 0.06 | **(0.03,0.10)***** | <0.001 | <0.001 | <0.001 |
|  |  | ≤ 60 years | 0.02 | (0.00,0.04) | Ref (0.00) | 0.01 | (-0.01,0.02) | 0.00 | (-0.02,0.02) | 0.01 | (0.00,0.03) | 0.711 | 0.161 |  |
|  | Model 3 | > 60 years | 0.01 | (-0.03,0.04) | Ref (0.00) | 0.00 | (-0.03,0.03) | 0.02 | (-0.01,0.05) | 0.03 | (0.00,0.06) | 0.025 | 0.033 | 0.001 |
|  |  | ≤ 60 years | 0.02 | (0.00,0.04) | Ref (0.00) | 0.00 | (-0.01,0.02) | 0.00 | (-0.02,0.02) | 0.00 | (-0.01,0.02) | 0.097 | 0.636 |  |
|  | Model 4 | > 60 years | 0.01 | (-0.02,0.05) | Ref (0.00) | 0.00 | (-0.03,0.02) | 0.01 | (-0.02,0.04) | 0.02 | (-0.01,0.05) | 0.428 | 0.230 | 0.001 |
|  |  | ≤ 60 years | 0.02 | (0.00,0.04) | Ref (0.00) | 0.00 | (-0.01,0.02) | 0.00 | (-0.02,0.02) | 0.01 | (-0.01,0.02) | 0.217 | 0.459 |  |
| Sex |  |  |  |  |  |  |  |  |  |  |  |  |  |  |
|  | Model 1 | Male | 0.01 | (-0.01,0.04) | Ref (0.00) | 0.02 | (-0.01,0.04) | 0.03 | (0.00,0.06) | 0.06 | **(0.04,0.08)***** | <0.001 | <0.001 | 0.239 |
|  |  | Female | 0.03 | (0.00,0.06) | Ref (0.00) | 0.02 | (-0.01,0.04) | 0.03 | **(0.01,0.05)**** | 0.07 | **(0.06,0.09)***** | <0.001 | <0.001 |  |
|  | Model 2 | Male | 0.02 | (0.00,0.04) | Ref (0.00) | 0.01 | (-0.01,0.04) | 0.02 | (-0.01,0.05) | 0.04 | **(0.02,0.06)***** | 0.007 | <0.001 | 0.853 |
|  |  | Female | 0.03 | **(0.01,0.06)*** | Ref (0.00) | 0.01 | (-0.01,0.03) | 0.02 | (0.00,0.04) | 0.05 | **(0.03,0.06)***** | 0.001 | <0.001 |  |
|  | Model 3 | Male | 0.02 | (0.00,0.04) | Ref (0.00) | 0.01 | (-0.01,0.03) | 0.02 | (-0.01,0.04) | 0.02 | (0.00,0.04) | 0.485 | 0.070 | 0.806 |
|  |  | Female | 0.03 | (0.00,0.05) | Ref (0.00) | 0.01 | (-0.01,0.03) | 0.01 | (0.00,0.03) | 0.04 | **(0.02,0.06)***** | 0.016 | <0.001 |  |
|  | Model 4 | Male | 0.02 | (0.00,0.04) | Ref (0.00) | 0.01 | (-0.01,0.03) | 0.01 | (-0.02,0.04) | 0.02 | (-0.01,0.04) | 0.866 | 0.143 | 0.889 |
|  |  | Female | 0.03 | **(0.01,0.06)*** | Ref (0.00) | 0.00 | (-0.02,0.02) | 0.01 | (-0.01,0.03) | 0.03 | **(0.01,0.05)**** | 0.232 | 0.006 |  |
| Race |  |  |  |  |  |  |  |  |  |  |  |  |  |  |
|  | Model 1 | Non-hispanic white | 0.02 | (0.00,0.04) | Ref (0.00) | 0.01 | (0.00,0.03) | 0.03 | (0.00,0.05) | 0.06 | **(0.04,0.08)***** | <0.001 | <0.001 | 0.562 |
|  |  | Others | 0.03 | (0.00,0.05) | Ref (0.00) | 0.01 | (-0.02,0.03) | 0.03 | (0.00,0.05) | 0.06 | **(0.04,0.08)***** | <0.001 | <0.001 |  |
|  | Model 2 | Non-hispanic white | 0.03 | **(0.01,0.05)**** | Ref (0.00) | 0.01 | (-0.01,0.03) | 0.02 | (0.00,0.04) | 0.04 | **(0.02,0.06)***** | 0.003 | <0.001 | 0.638 |
|  |  | Others | 0.02 | (0.00,0.05) | Ref (0.00) | 0.00 | (-0.02,0.02) | 0.02 | (-0.01,0.04) | 0.04 | **(0.02,0.06)***** | 0.001 | <0.001 |  |
|  | Model 3 | Non-hispanic white | 0.02 | **(0.01,0.04)*** | Ref (0.00) | 0.01 | (-0.01,0.02) | 0.01 | (-0.01,0.03) | 0.02 | **(0.01,0.04)**** | 0.282 | 0.010 | 0.794 |
|  |  | Others | 0.03 | (0.00,0.05) | Ref (0.00) | 0.00 | (-0.02,0.03) | 0.01 | (-0.01,0.04) | 0.03 | **(0.02,0.05)***** | 0.009 | <0.001 |  |
|  | Model 4 | Non-hispanic white | 0.03 | **(0.01,0.04)**** | Ref (0.00) | 0.00 | (-0.01,0.02) | 0.01 | (-0.01,0.03) | 0.02 | (0.00,0.04) | 0.654 | 0.028 | 0.817 |
|  |  | Others | 0.03 | **(0.01,0.05)**** | Ref (0.00) | 0.00 | (-0.02,0.02) | 0.01 | (-0.02,0.03) | 0.02 | (0.00,0.04) | 0.548 | 0.038 |  |
| BMI |  |  |  |  |  |  |  |  |  |  |  |  |  |  |
|  | Model 1 | < 30 kg/m^2^ | 0.02 | **(0.01,0.04)**** | Ref (0.00) | 0.01 | (-0.01,0.03) | 0.03 | **(0.01,0.05)**** | 0.06 | **(0.04,0.08)***** | <0.001 | <0.001 | 0.244 |
|  |  | ≥ 30 kg/m^2^ | 0.02 | (-0.01,0.05) | Ref (0.00) | 0.03 | (0.00,0.05) | 0.01 | (-0.02,0.04) | 0.06 | **(0.04,0.08)***** | <0.001 | <0.001 |  |
|  | Model 2 | < 30 kg/m^2^ | 0.02 | **(0.01,0.04)**** | Ref (0.00) | 0.01 | (-0.01,0.02) | 0.03 | **(0.01,0.05)*** | 0.04 | **(0.03,0.06)***** | <0.001 | <0.001 | 0.297 |
|  |  | ≥ 30 kg/m^2^ | 0.02 | (-0.01,0.05) | Ref (0.00) | 0.02 | (-0.01,0.05) | 0.01 | (-0.02,0.04) | 0.05 | **(0.03,0.07)***** | 0.001 | <0.001 |  |
|  | Model 3 | < 30 kg/m^2^ | 0.03 | **(0.01,0.04)***** | Ref (0.00) | 0.00 | (-0.01,0.02) | 0.02 | (0.00,0.04) | 0.03 | **(0.01,0.04)***** | 0.113 | 0.001 | 0.174 |
|  |  | ≥ 30 kg/m^2^ | 0.01 | (-0.02,0.04) | Ref (0.00) | 0.02 | (-0.01,0.04) | 0.00 | (-0.03,0.03) | 0.04 | **(0.02,0.06)***** | 0.012 | <0.001 |  |
|  | Model 4 | < 30 kg/m^2^ | 0.03 | **(0.01,0.04)***** | Ref (0.00) | 0.00 | (-0.01,0.02) | 0.02 | (0.00,0.04) | 0.03 | **(0.01,0.04)**** | 0.256 | 0.003 | 0.173 |
|  |  | ≥ 30 kg/m^2^ | 0.02 | (-0.01,0.05) | Ref (0.00) | 0.01 | (-0.02,0.04) | -0.01 | (-0.04,0.02) | 0.02 | (0.00,0.04) | 0.672 | 0.065 |  |
| Smoking |  |  |  |  |  |  |  |  |  |  |  |  |  |  |
|  | Model 1 | Yes | 0.02 | (-0.01,0.05) | Ref (0.00) | 0.00 | (-0.02,0.02) | 0.03 | (0.00,0.06) | 0.06 | **(0.03,0.08)***** | <0.001 | <0.001 | 0.959 |
|  |  | No | 0.02 | (-0.01,0.04) | Ref (0.00) | 0.03 | **(0.01,0.05)**** | 0.03 | **(0.01,0.05)**** | 0.07 | **(0.06,0.09)***** | <0.001 | <0.001 |  |
|  | Model 2 | Yes | 0.02 | (0.00,0.05) | Ref (0.00) | 0.00 | (-0.03,0.02) | 0.02 | (-0.01,0.05) | 0.04 | **(0.02,0.06)***** | 0.025 | 0.001 | 0.557 |
|  |  | No | 0.02 | (0.00,0.04) | Ref (0.00) | 0.02 | (0.00,0.04) | 0.02 | (0.00,0.04) | 0.05 | **(0.03,0.06)***** | <0.001 | <0.001 |  |
|  | Model 3 | Yes | 0.02 | (0.00,0.05) | Ref (0.00) | -0.01 | (-0.03,0.02) | 0.02 | (-0.01,0.05) | 0.03 | (0.00,0.05) | 0.265 | 0.022 | 0.514 |
|  |  | No | 0.02 | (0.00,0.04) | Ref (0.00) | 0.02 | (0.00,0.03) | 0.01 | (-0.01,0.03) | 0.03 | **(0.01,0.05)***** | 0.017 | 0.001 |  |
|  | Model 4 | Yes | 0.02 | (0.00,0.05) | Ref (0.00) | -0.01 | (-0.03,0.02) | 0.02 | (-0.01,0.04) | 0.02 | (0.00,0.05) | 0.504 | 0.044 | 0.548 |
|  |  | No | 0.02 | (0.00,0.05) | Ref (0.00) | 0.01 | (-0.01,0.03) | 0.01 | (-0.02,0.03) | 0.03 | **(0.01,0.04)*** | 0.277 | 0.012 |  |
| Drinking |  |  |  |  |  |  |  |  |  |  |  |  |  |  |
|  | Model 1 | Yes | 0.02 | **(0.01,0.04)*** | Ref (0.00) | 0.01 | (0.00,0.03) | 0.03 | **(0.01,0.05)**** | 0.07 | **(0.05,0.08)***** | <0.001 | <0.001 | 0.197 |
|  |  | No | 0.00 | (-0.05,0.04) | Ref (0.00) | 0.02 | (-0.01,0.05) | 0.02 | (-0.01,0.05) | 0.05 | **(0.02,0.08)***** | 0.001 | 0.001 |  |
|  | Model 2 | Yes | 0.02 | **(0.01,0.04)**** | Ref (0.00) | 0.01 | (-0.01,0.03) | 0.02 | (0.00,0.04) | 0.05 | **(0.03,0.06)***** | <0.001 | <0.001 | 0.144 |
|  |  | No | 0.01 | (-0.03,0.05) | Ref (0.00) | 0.01 | (-0.02,0.04) | 0.01 | (-0.03,0.04) | 0.03 | (0.00,0.06) | 0.129 | 0.042 |  |
|  | Model 3 | Yes | 0.02 | **(0.01,0.04)**** | Ref (0.00) | 0.01 | (-0.01,0.02) | 0.01 | (-0.01,0.03) | 0.03 | **(0.02,0.05)***** | 0.010 | <0.001 | 0.166 |
|  |  | No | 0.01 | (-0.03,0.04) | Ref (0.00) | 0.01 | (-0.02,0.03) | 0.00 | (-0.03,0.03) | 0.02 | (-0.01,0.05) | 0.298 | 0.185 |  |
|  | Model 4 | Yes | 0.02 | **(0.01,0.04)**** | Ref (0.00) | 0.01 | (-0.01,0.02) | 0.01 | (-0.01,0.03) | 0.03 | **(0.01,0.05)***** | 0.100 | <0.001 | 0.171 |
|  |  | No | 0.01 | (-0.03,0.05) | Ref (0.00) | 0.00 | (-0.03,0.03) | -0.01 | (-0.04,0.03) | 0.01 | (-0.02,0.03) | 0.934 | 0.720 |  |
| Exercise |  |  |  |  |  |  |  |  |  |  |  |  |  |  |
|  | Model 1 | Yes | 0.01 | (-0.01,0.03) | Ref (0.00) | 0.00 | (-0.02,0.03) | 0.03 | (0.00,0.06) | 0.05 | **(0.03,0.07)***** | <0.001 | <0.001 | 0.625 |
|  |  | No | 0.03 | **(0.01,0.05)**** | Ref (0.00) | 0.02 | (0.00,0.04) | 0.03 | (0.00,0.05) | 0.07 | **(0.05,0.08)***** | <0.001 | <0.001 |  |
|  | Model 2 | Yes | 0.02 | (-0.01,0.04) | Ref (0.00) | 0.01 | (-0.02,0.03) | 0.02 | (0.00,0.05) | 0.04 | **(0.01,0.06)***** | 0.020 | 0.001 | 0.764 |
|  |  | No | 0.03 | **(0.01,0.05)**** | Ref (0.00) | 0.01 | (-0.01,0.03) | 0.02 | (-0.01,0.04) | 0.05 | **(0.03,0.06)***** | <0.001 | <0.001 |  |
|  | Model 3 | Yes | 0.01 | (-0.01,0.04) | Ref (0.00) | 0.00 | (-0.02,0.02) | 0.02 | (-0.01,0.04) | 0.02 | (0.00,0.05) | 0.155 | 0.038 | 0.835 |
|  |  | No | 0.03 | **(0.01,0.05)***** | Ref (0.00) | 0.01 | (-0.01,0.03) | 0.01 | (-0.01,0.03) | 0.03 | **(0.02,0.05)***** | 0.086 | <0.001 |  |
|  | Model 4 | Yes | 0.02 | (-0.01,0.04) | Ref (0.00) | 0.00 | (-0.03,0.02) | 0.01 | (-0.01,0.04) | 0.01 | (-0.01,0.04) | 0.783 | 0.260 | 0.851 |
|  |  | No | 0.03 | **(0.02,0.05)***** | Ref (0.00) | 0.01 | (-0.01,0.03) | 0.01 | (-0.01,0.03) | 0.03 | **(0.01,0.05)***** | 0.348 | 0.001 |  |
| Education |  |  |  |  |  |  |  |  |  |  |  |  |  |  |
|  | Model 1 | Above high school | 0.02 | (0.00,0.04) | Ref (0.00) | 0.01 | (-0.01,0.03) | 0.02 | (-0.01,0.04) | 0.06 | **(0.05,0.08)***** | <0.001 | <0.001 | 0.795 |
|  |  | Others | 0.02 | (-0.01,0.04) | Ref (0.00) | 0.02 | (-0.01,0.04) | 0.04 | **(0.01,0.07)**** | 0.05 | **(0.03,0.06)***** | <0.001 | <0.001 |  |
|  | Model 2 | Above high school | 0.02 | (0.00,0.04) | Ref (0.00) | 0.01 | (-0.01,0.03) | 0.01 | (-0.01,0.03) | 0.05 | **(0.03,0.07)***** | <0.001 | <0.001 | 0.645 |
|  |  | Others | 0.02 | (0.00,0.05) | Ref (0.00) | 0.01 | (-0.01,0.03) | 0.03 | **(0.01,0.06)*** | 0.03 | **(0.02,0.05)***** | 0.023 | <0.001 |  |
|  | Model 3 | Above high school | 0.02 | (0.00,0.04) | Ref (0.00) | 0.01 | (-0.01,0.03) | 0.01 | (-0.02,0.03) | 0.04 | **(0.02,0.06)***** | 0.006 | <0.001 | 0.762 |
|  |  | Others | 0.02 | (0.00,0.05) | Ref (0.00) | 0.01 | (-0.02,0.03) | 0.03 | (0.00,0.05) | 0.02 | (0.00,0.04) | 0.439 | 0.038 |  |
|  | Model 4 | Above high school | 0.03 | **(0.01,0.04)**** | Ref (0.00) | 0.00 | (-0.02,0.02) | 0.00 | (-0.02,0.02) | 0.03 | **(0.01,0.05)**** | 0.298 | 0.003 | 0.779 |
|  |  | Others | 0.02 | (0.00,0.05) | Ref (0.00) | 0.01 | (-0.02,0.03) | 0.02 | (0.00,0.05) | 0.02 | (0.00,0.04) | 0.480 | 0.056 |  |
| Income |  |  |  |  |  |  |  |  |  |  |  |  |  |  |
|  | Model 1 | ≤ $55,000 | 0.02 | (0.00,0.05) | Ref (0.00) | 0.02 | (0.00,0.04) | 0.02 | (0.00,0.04) | 0.06 | **(0.05,0.08)***** | <0.001 | <0.001 | 0.788 |
|  |  | > $55,000 | 0.01 | (-0.01,0.04) | Ref (0.00) | 0.00 | (-0.02,0.02) | 0.03 | (0.00,0.05) | 0.05 | **(0.03,0.07)***** | <0.001 | <0.001 |  |
|  | Model 2 | ≤ $55,000 | 0.03 | **(0.01,0.05)*** | Ref (0.00) | 0.02 | (0.00,0.03) | 0.01 | (-0.01,0.03) | 0.04 | **(0.03,0.06)***** | 0.001 | <0.001 | 0.960 |
|  |  | > $55,000 | 0.02 | (-0.01,0.04) | Ref (0.00) | 0.01 | (-0.02,0.03) | 0.02 | (0.00,0.05) | 0.04 | **(0.02,0.06)***** | 0.005 | <0.001 |  |
|  | Model 3 | ≤ $55,000 | 0.02 | (0.00,0.04) | Ref (0.00) | 0.01 | (-0.01,0.03) | 0.01 | (-0.01,0.03) | 0.03 | **(0.02,0.05)***** | 0.047 | <0.001 | 0.961 |
|  |  | > $55,000 | 0.02 | (-0.01,0.04) | Ref (0.00) | 0.00 | (-0.02,0.02) | 0.02 | (-0.01,0.04) | 0.02 | (0.00,0.04) | 0.228 | 0.026 |  |
|  | Model 4 | ≤ $55,000 | 0.02 | **(0.01,0.04)*** | Ref (0.00) | 0.01 | (-0.01,0.03) | 0.01 | (-0.01,0.03) | 0.03 | **(0.01,0.05)***** | 0.096 | 0.001 | 0.921 |
|  |  | > $55,000 | 0.02 | (0.00,0.04) | Ref (0.00) | 0.00 | (-0.02,0.02) | 0.01 | (-0.01,0.04) | 0.01 | (-0.01,0.04) | 0.937 | 0.171 |  |
| Sleep duration |  |  |  |  |  |  |  |  |  |  |  |  |  |  |
|  | Model 1 | ≤ 7 hours | 0.02 | (-0.01,0.04) | Ref (0.00) | 0.02 | (0.00,0.05) | 0.03 | **(0.01,0.06)*** | 0.07 | **(0.05,0.10)***** | <0.001 | <0.001 | 0.004 |
|  |  | > 7 hours | 0.03 | **(0.01,0.05)*** | Ref (0.00) | 0.01 | (-0.01,0.03) | 0.03 | **(0.01,0.05)**** | 0.06 | **(0.04,0.08)***** | <0.001 | <0.001 |  |
|  | Model 2 | ≤ 7 hours | 0.02 | (-0.01,0.04) | Ref (0.00) | 0.01 | (-0.01,0.04) | 0.02 | (0.00,0.05) | 0.05 | **(0.02,0.07)***** | <0.001 | <0.001 | 0.023 |
|  |  | > 7 hours | 0.03 | **(0.01,0.05)**** | Ref (0.00) | 0.01 | (-0.01,0.03) | 0.02 | (0.00,0.04) | 0.04 | **(0.03,0.06)***** | 0.002 | <0.001 |  |
|  | Model 3 | ≤ 7 hours | 0.01 | (-0.01,0.04) | Ref (0.00) | 0.01 | (-0.01,0.03) | 0.02 | (0.00,0.05) | 0.04 | **(0.01,0.06)**** | 0.004 | 0.005 | 0.083 |
|  |  | > 7 hours | 0.03 | **(0.01,0.05)***** | Ref (0.00) | 0.00 | (-0.01,0.02) | 0.01 | (-0.01,0.03) | 0.03 | **(0.01,0.04)**** | 0.471 | 0.002 |  |
|  | Model 4 | ≤ 7 hours | 0.02 | (-0.01,0.04) | Ref (0.00) | 0.01 | (-0.02,0.03) | 0.02 | (-0.01,0.04) | 0.03 | (0.00,0.05) | 0.106 | 0.032 | 0.082 |
|  |  | > 7 hours | 0.03 | **(0.01,0.05)***** | Ref (0.00) | 0.00 | (-0.02,0.02) | 0.01 | (-0.01,0.03) | 0.02 | **(0.01,0.04)*** | 0.872 | 0.011 |  |
| Daily energy intake |  |  |  |  |  |  |  |  |  |  |  |  |  |  |
|  | Model 1 | Higher | 0.03 | (0.00,0.05) | Ref (0.00) | 0.02 | (0.00,0.05) | 0.04 | **(0.01,0.06)**** | 0.07 | **(0.05,0.09)***** | <0.001 | <0.001 | 0.016 |
|  |  | Lower | 0.02 | (0.00,0.04) | Ref (0.00) | 0.01 | (-0.01,0.03) | 0.02 | (0.00,0.04) | 0.06 | **(0.04,0.07)***** | <0.001 | <0.001 |  |
|  | Model 2 | Higher | 0.03 | **(0.01,0.05)*** | Ref (0.00) | 0.02 | (-0.01,0.04) | 0.02 | (-0.01,0.04) | 0.05 | **(0.03,0.07)***** | 0.072 | <0.001 | 0.047 |
|  |  | Lower | 0.02 | (0.00,0.04) | Ref (0.00) | 0.00 | (-0.01,0.02) | 0.01 | (-0.01,0.03) | 0.04 | **(0.02,0.05)***** | 0.002 | <0.001 |  |
|  | Model 3 | Higher | 0.03 | **(0.01,0.05)*** | Ref (0.00) | 0.02 | (-0.01,0.04) | 0.01 | (-0.01,0.04) | 0.04 | **(0.02,0.06)***** | 0.190 | <0.001 | 0.041 |
|  |  | Lower | 0.02 | (0.00,0.04) | Ref (0.00) | 0.00 | (-0.02,0.02) | 0.02 | (-0.01,0.04) | 0.03 | **(0.01,0.05)***** | 0.003 | <0.001 |  |
|  | Model 4 | Higher | 0.03 | **(0.01,0.05)*** | Ref (0.00) | 0.02 | (-0.01,0.04) | 0.01 | (-0.01,0.04) | 0.04 | **(0.02,0.06)***** | 0.259 | 0.001 | 0.044 |
|  |  | Lower | 0.02 | (0.00,0.04) | Ref (0.00) | 0.00 | (-0.02,0.02) | 0.01 | (-0.01,0.03) | 0.02 | (0.00,0.03) | 0.628 | 0.093 |  |
| Healthy eating index-2015 |  |  |  |  |  |  |  |  |  |  |  |  |  |  |
|  | Model 1 | < 30.53 | 0.02 | (-0.01,0.05) | Ref (0.00) | 0.01 | (-0.01,0.03) | 0.02 | (-0.01,0.04) | 0.06 | **(0.04,0.08)***** | <0.001 | <0.001 | 0.975 |
|  |  | ≥ 30.53 | 0.02 | (0.00,0.04) | Ref (0.00) | 0.02 | (0.00,0.04) | 0.04 | **(0.01,0.06)**** | 0.06 | **(0.05,0.08)***** | <0.001 | <0.001 |  |
|  | Model 2 | < 30.53 | 0.03 | (0.00,0.05) | Ref (0.00) | 0.01 | (-0.01,0.03) | 0.01 | (-0.02,0.03) | 0.04 | **(0.02,0.06)***** | 0.005 | <0.001 | 0.705 |
|  |  | ≥ 30.53 | 0.02 | (0.00,0.04) | Ref (0.00) | 0.01 | (-0.01,0.03) | 0.03 | **(0.01,0.05)*** | 0.04 | **(0.02,0.06)***** | <0.001 | <0.001 |  |
|  | Model 3 | < 30.53 | 0.03 | (0.00,0.05) | Ref (0.00) | 0.01 | (-0.01,0.02) | 0.01 | (-0.02,0.03) | 0.03 | **(0.01,0.05)***** | 0.077 | 0.001 | 0.711 |
|  |  | ≥ 30.53 | 0.02 | (0.00,0.04) | Ref (0.00) | 0.01 | (-0.01,0.03) | 0.02 | (0.00,0.04) | 0.03 | **(0.01,0.05)**** | 0.050 | 0.002 |  |
|  | Model 4 | < 30.53 | 0.03 | (0.00,0.05) | Ref (0.00) | 0.01 | (-0.01,0.02) | 0.01 | (-0.02,0.03) | 0.03 | **(0.01,0.05)**** | 0.140 | 0.002 | 0.746 |
|  |  | ≥ 30.53 | 0.02 | (0.00,0.04) | Ref (0.00) | 0.01 | (-0.01,0.03) | 0.02 | (-0.01,0.04) | 0.02 | (0.00,0.04) | 0.645 | 0.053 |  |
| Dietary supplements use |  |  |  |  |  |  |  |  |  |  |  |  |  |  |
|  | Model 1 | Yes | 0.02 | (0.00,0.05) | Ref (0.00) | 0.01 | (-0.01,0.03) | 0.03 | **(0.01,0.06)**** | 0.06 | **(0.04,0.08)***** | <0.001 | <0.001 | 0.663 |
|  |  | No | 0.02 | (-0.01,0.04) | Ref (0.00) | 0.02 | (0.00,0.04) | 0.02 | (-0.01,0.05) | 0.06 | **(0.04,0.08)***** | <0.001 | <0.001 |  |
|  | Model 2 | Yes | 0.03 | (0.00,0.05) | Ref (0.00) | 0.01 | (-0.01,0.03) | 0.03 | **(0.01,0.05)*** | 0.04 | **(0.02,0.07)***** | 0.004 | <0.001 | 0.692 |
|  |  | No | 0.02 | (0.00,0.04) | Ref (0.00) | 0.01 | (-0.01,0.04) | 0.01 | (-0.02,0.03) | 0.04 | **(0.02,0.06)***** | 0.002 | <0.001 |  |
|  | Model 3 | Yes | 0.03 | (0.00,0.05) | Ref (0.00) | 0.00 | (-0.02,0.02) | 0.02 | (0.00,0.05) | 0.03 | **(0.01,0.05)**** | 0.139 | 0.010 | 0.477 |
|  |  | No | 0.02 | (0.00,0.04) | Ref (0.00) | 0.01 | (-0.01,0.03) | 0.00 | (-0.02,0.03) | 0.03 | **(0.01,0.05)***** | 0.047 | 0.001 |  |
|  | Model 4 | Yes | 0.03 | **(0.01,0.05)*** | Ref (0.00) | 0.00 | (-0.02,0.02) | 0.02 | (0.00,0.04) | 0.02 | (0.00,0.04) | 0.708 | 0.060 | 0.434 |
|  |  | No | 0.02 | (0.00,0.04) | Ref (0.00) | 0.01 | (-0.01,0.03) | 0.00 | (-0.02,0.03) | 0.03 | **(0.01,0.05)**** | 0.178 | 0.003 |  |
| Daily eating frequency |  |  |  |  |  |  |  |  |  |  |  |  |  |  |
|  | Model 1 | < 4.00 times | 0.04 | **(0.01,0.06)**** | Ref (0.00) | 0.02 | (-0.01,0.04) | 0.02 | (-0.01,0.04) | 0.05 | **(0.03,0.07)***** | <0.001 | <0.001 | 0.002 |
|  |  | ≥ 4.00 times | 0.02 | (0.00,0.04) | Ref (0.00) | 0.00 | (-0.02,0.02) | 0.03 | (0.00,0.06) | 0.05 | **(0.03,0.08)***** | 0.153 | <0.001 |  |
|  | Model 2 | < 4.00 times | 0.03 | **(0.01,0.06)*** | Ref (0.00) | 0.01 | (-0.01,0.03) | 0.01 | (-0.02,0.03) | 0.03 | **(0.02,0.05)***** | 0.004 | <0.001 | 0.016 |
|  |  | ≥ 4.00 times | 0.03 | **(0.01,0.04)**** | Ref (0.00) | 0.00 | (-0.02,0.02) | 0.02 | (-0.01,0.05) | 0.04 | **(0.01,0.06)**** | 0.806 | 0.004 |  |
|  | Model 3 | < 4.00 times | 0.03 | (0.00,0.05) | Ref (0.00) | 0.01 | (-0.01,0.03) | 0.00 | (-0.02,0.03) | 0.02 | **(0.01,0.04)*** | 0.064 | 0.012 | 0.029 |
|  |  | ≥ 4.00 times | 0.02 | **(0.01,0.04)**** | Ref (0.00) | 0.00 | (-0.02,0.02) | 0.02 | (-0.01,0.05) | 0.03 | (0.00,0.05) | 0.766 | 0.021 |  |

^a^The variables adjusted in each model were the factors mentioned above except the stratification variables.

^b^Data were listed as the weighted beta estimates and 95% confidence intervals, with *p < 0.05, **p < 0.01, ***p < 0.001.

^c^Q, quintile.

^d^Ref, reference.

^e^Tests for trends based on the variables containing the median values for each quartile.

^f^*P*_test_ was the result of Bonfreni correction.

^g^Multiplicative interaction was assessed by adding interaction terms to the models.

**Supplementary table 11.** Association of NFD with KDM residual (years) stratified by variables of interest

|  |  |  | Nighttime fasting duration (hours) | | | | | | | | |  |  |  |
| --- | --- | --- | --- | --- | --- | --- | --- | --- | --- | --- | --- | --- | --- | --- |
|  |  |  | Q1^c^ | | Q2 | Q3 | | Q4 | | Q5 | | *P*_trend_^e^ | *P*_test_^f^ | *P*_interaction_^g^ |
|  |  |  | β^b^ | 95% CI | β | β | 95% CI | β | 95% CI | β | 95% CI |  |  |  |
| Age |  |  |  |  |  |  |  |  |  |  |  |  |  |  |
|  | Model 1^a^ | > 60 years | 0.06 | (-0.78, 0.91) | Ref (0.00)^d^ | 0.17 | (-0.53, 0.88) | 0.02 | (-0.74, 0.79) | 2.06 | **(1.39, 2.73)***** | < 0.001 | < 0.001 | < 0.001 |
|  |  | ≤ 60 years | 0.42 | (-0.06, 0.89) | Ref (0.00) | 0.51 | **(0.08, 0.94)*** | 1.07 | **(0.58, 1.55)***** | 1.76 | **(1.40, 2.11)***** | < 0.001 | < 0.001 |  |
|  | Model 2 | > 60 years | 0.27 | (-0.54, 1.08) | Ref (0.00) | 0.15 | (-0.55, 0.84) | 0.01 | (-0.76, 0.77) | 1.74 | **(1.06, 2.43)***** | < 0.001 | < 0.001 | < 0.001 |
|  |  | ≤ 60 years | 0.48 | **(0.07, 0.90)*** | Ref (0.00) | 0.37 | (-0.04, 0.77) | 0.74 | **(0.28, 1.21)**** | 1.21 | **(0.90, 1.51)***** | < 0.001 | < 0.001 |  |
|  | Model 3 | > 60 years | 0.15 | (-0.60, 0.90) | Ref (0.00) | 0.00 | (-0.69, 0.69) | -0.13 | (-0.92, 0.65) | 1.23 | **(0.52, 1.94)***** | 0.003 | 0.001 | < 0.001 |
|  |  | ≤ 60 years | 0.31 | (-0.09, 0.72) | Ref (0.00) | 0.32 | (-0.07, 0.71) | 0.73 | **(0.27, 1.19)**** | 1.09 | **(0.79, 1.38)***** | < 0.001 | < 0.001 |  |
|  | Model 4 | > 60 years | 0.27 | (-0.48, 1.01) | Ref (0.00) | -0.08 | (-0.77, 0.62) | -0.26 | (-1.10, 0.57) | 1.00 | **(0.23, 1.77)*** | 0.054 | 0.012 | < 0.001 |
|  |  | ≤ 60 years | 0.42 | **(0.01, 0.84)*** | Ref (0.00) | 0.22 | (-0.17, 0.61) | 0.54 | **(0.09, 1.00)*** | 0.73 | **(0.40, 1.06)***** | 0.019 | < 0.001 |  |
| Sex |  |  |  |  |  |  |  |  |  |  |  |  |  |  |
|  | Model 1 | Male | 0.27 | (-0.17, 0.70) | Ref (0.00) | 0.41 | (-0.13, 0.94) | 0.85 | **(0.18, 1.51)*** | 1.41 | **(0.95, 1.86)***** | < 0.001 | < 0.001 | 0.678 |
|  |  | Female | 0.23 | (-0.35, 0.81) | Ref (0.00) | 0.15 | (-0.25, 0.56) | 0.31 | (-0.12, 0.73) | 1.17 | **(0.80, 1.54)***** | < 0.001 | < 0.001 |  |
|  | Model 2 | Male | 0.36 | (-0.04, 0.75) | Ref (0.00) | 0.38 | (-0.11, 0.87) | 0.55 | (-0.09, 1.19) | 0.91 | **(0.47, 1.34)***** | 0.004 | < 0.001 | 0.408 |
|  |  | Female | 0.35 | (-0.18, 0.87) | Ref (0.00) | 0.00 | (-0.37, 0.37) | 0.11 | (-0.29, 0.51) | 0.69 | **(0.35, 1.04)***** | 0.006 | < 0.001 |  |
|  | Model 3 | Male | 0.32 | (-0.06, 0.71) | Ref (0.00) | 0.30 | (-0.16, 0.76) | 0.49 | (-0.14, 1.12) | 0.63 | **(0.20, 1.05)**** | 0.064 | 0.004 | 0.449 |
|  |  | Female | 0.24 | (-0.26, 0.74) | Ref (0.00) | -0.04 | (-0.40, 0.31) | 0.04 | (-0.34, 0.42) | 0.54 | **(0.17, 0.90)**** | 0.036 | 0.005 |  |
|  | Model 4 | Male | 0.39 | (-0.01, 0.79) | Ref (0.00) | 0.24 | (-0.21, 0.70) | 0.39 | (-0.25, 1.03) | 0.44 | (-0.01, 0.89) | 0.507 | 0.056 | 0.358 |
|  |  | Female | 0.32 | (-0.19, 0.82) | Ref (0.00) | -0.11 | (-0.47, 0.25) | -0.08 | (-0.48, 0.32) | 0.30 | (-0.10, 0.70) | 0.664 | 0.141 |  |
| Race |  |  |  |  |  |  |  |  |  |  |  |  |  |  |
|  | Model 1 | Non-hispanic white | 0.22 | (-0.21, 0.64) | Ref (0.00) | 0.28 | (-0.19, 0.74) | 0.42 | (-0.07, 0.91) | 1.26 | **(0.88, 1.64)***** | < 0.001 | < 0.001 | 0.555 |
|  |  | Others | 0.41 | (-0.06, 0.88) | Ref (0.00) | 0.16 | (-0.29, 0.60) | 0.75 | **(0.23, 1.26)**** | 1.02 | **(0.67, 1.37)***** | < 0.001 | < 0.001 |  |
|  | Model 2 | Non-hispanic white | 0.35 | (-0.02, 0.71) | Ref (0.00) | 0.18 | (-0.26, 0.63) | 0.24 | (-0.24, 0.72) | 0.85 | **(0.52, 1.18)***** | < 0.001 | < 0.001 | 0.402 |
|  |  | Others | 0.39 | (-0.06, 0.83) | Ref (0.00) | 0.08 | (-0.36, 0.51) | 0.46 | (0.00, 0.92) | 0.58 | **(0.22, 0.95)**** | 0.016 | 0.002 |  |
|  | Model 3 | Non-hispanic white | 0.27 | (-0.07, 0.61) | Ref (0.00) | 0.06 | (-0.38, 0.49) | 0.13 | (-0.34, 0.59) | 0.51 | **(0.18, 0.83)**** | 0.056 | 0.003 | 0.327 |
|  |  | Others | 0.35 | (-0.07, 0.78) | Ref (0.00) | 0.14 | (-0.30, 0.58) | 0.44 | (-0.01, 0.90) | 0.55 | **(0.18, 0.91)**** | 0.029 | 0.004 |  |
|  | Model 4 | Non-hispanic white | 0.31 | (-0.05, 0.67) | Ref (0.00) | 0.02 | (-0.41, 0.46) | 0.07 | (-0.42, 0.55) | 0.40 | **(0.04, 0.76)*** | 0.457 | 0.031 | 0.311 |
|  |  | Others | 0.48 | **(0.05, 0.92)*** | Ref (0.00) | 0.02 | (-0.42, 0.46) | 0.23 | (-0.21, 0.67) | 0.11 | (-0.31, 0.53) | 0.478 | 0.602 |  |
| BMI |  |  |  |  |  |  |  |  |  |  |  |  |  |  |
|  | Model 1 | < 30 kg/m^2^ | 0.29 | (-0.07, 0.65) | Ref (0.00) | 0.20 | (-0.18, 0.58) | 0.53 | **(0.05, 1.00)*** | 1.18 | **(0.85, 1.51)***** | < 0.001 | < 0.001 | 0.227 |
|  |  | ≥ 30 kg/m^2^ | 0.26 | (-0.40, 0.92) | Ref (0.00) | 0.38 | (-0.21, 0.97) | 0.22 | (-0.37, 0.80) | 0.94 | **(0.52, 1.36)***** | 0.001 | < 0.001 |  |
|  | Model 2 | < 30 kg/m^2^ | 0.30 | (-0.04, 0.65) | Ref (0.00) | 0.14 | (-0.22, 0.51) | 0.43 | (-0.05, 0.90) | 0.88 | **(0.57, 1.20)***** | < 0.001 | < 0.001 | 0.163 |
|  |  | ≥ 30 kg/m^2^ | 0.32 | (-0.34, 0.97) | Ref (0.00) | 0.30 | (-0.29, 0.89) | 0.11 | (-0.48, 0.70) | 0.72 | **(0.30, 1.14)***** | 0.027 | 0.001 |  |
|  | Model 3 | < 30 kg/m^2^ | 0.28 | (-0.05, 0.62) | Ref (0.00) | 0.09 | (-0.27, 0.44) | 0.39 | (-0.06, 0.85) | 0.62 | **(0.32, 0.91)***** | 0.003 | < 0.001 | 0.339 |
|  |  | ≥ 30 kg/m^2^ | 0.17 | (-0.46, 0.79) | Ref (0.00) | 0.20 | (-0.37, 0.76) | -0.08 | (-0.66, 0.50) | 0.48 | **(0.04, 0.91)*** | 0.146 | 0.033 |  |
|  | Model 4 | < 30 kg/m^2^ | 0.34 | (0.00, 0.67) | Ref (0.00) | 0.04 | (-0.31, 0.40) | 0.31 | (-0.15, 0.77) | 0.46 | **(0.15, 0.78)**** | 0.143 | 0.005 | 0.344 |
|  |  | ≥ 30 kg/m^2^ | 0.29 | (-0.36, 0.95) | Ref (0.00) | 0.06 | (-0.51, 0.62) | -0.29 | (-0.89, 0.31) | 0.07 | (-0.44, 0.59) | 0.628 | 0.777 |  |
| Smoking |  |  |  |  |  |  |  |  |  |  |  |  |  |  |
|  | Model 1 | Yes | 0.21 | (-0.29, 0.71) | Ref (0.00) | -0.02 | (-0.50, 0.46) | 0.45 | (-0.22, 1.12) | 1.08 | **(0.60, 1.55)***** | < 0.001 | < 0.001 | 0.162 |
|  |  | No | 0.11 | (-0.43, 0.64) | Ref (0.00) | 0.50 | **(0.09, 0.90)*** | 0.62 | **(0.22, 1.03)**** | 1.36 | **(0.96, 1.75)***** | < 0.001 | < 0.001 |  |
|  | Model 2 | Yes | 0.32 | (-0.13, 0.77) | Ref (0.00) | 0.00 | (-0.48, 0.48) | 0.27 | (-0.36, 0.91) | 0.69 | **(0.24, 1.14)**** | 0.035 | 0.003 | 0.600 |
|  |  | No | 0.23 | (-0.26, 0.73) | Ref (0.00) | 0.28 | (-0.10, 0.67) | 0.31 | (-0.10, 0.72) | 0.83 | **(0.45, 1.20)***** | < 0.001 | < 0.001 |  |
|  | Model 3 | Yes | 0.24 | (-0.20, 0.68) | Ref (0.00) | -0.09 | (-0.55, 0.36) | 0.19 | (-0.42, 0.80) | 0.41 | (-0.04, 0.86) | 0.264 | 0.074 | 0.727 |
|  |  | No | 0.20 | (-0.26, 0.67) | Ref (0.00) | 0.22 | (-0.16, 0.59) | 0.19 | (-0.20, 0.59) | 0.58 | **(0.20, 0.97)**** | 0.006 | 0.003 |  |
|  | Model 4 | Yes | 0.26 | (-0.18, 0.71) | Ref (0.00) | -0.11 | (-0.57, 0.34) | 0.16 | (-0.46, 0.77) | 0.34 | (-0.14, 0.82) | 0.565 | 0.165 | 0.638 |
|  |  | No | 0.31 | (-0.17, 0.79) | Ref (0.00) | 0.12 | (-0.25, 0.50) | 0.02 | (-0.40, 0.45) | 0.28 | (-0.13, 0.69) | 0.726 | 0.179 |  |
| Drinking |  |  |  |  |  |  |  |  |  |  |  |  |  |  |
|  | Model 1 | Yes | 0.22 | (-0.15, 0.58) | Ref (0.00) | 0.30 | (-0.11, 0.71) | 0.52 | **(0.05, 0.99)*** | 1.34 | **(1.00, 1.68)***** | < 0.001 | < 0.001 | 0.352 |
|  |  | No | 0.10 | (-0.82, 1.01) | Ref (0.00) | 0.16 | (-0.49, 0.81) | 0.48 | (-0.14, 1.10) | 1.02 | **(0.36, 1.69)**** | 0.010 | 0.003 |  |
|  | Model 2 | Yes | 0.22 | (-0.09, 0.53) | Ref (0.00) | 0.25 | (-0.14, 0.64) | 0.30 | (-0.17, 0.77) | 0.86 | **(0.53, 1.18)***** | < 0.001 | < 0.001 | 0.179 |
|  |  | No | 0.40 | (-0.42, 1.22) | Ref (0.00) | 0.04 | (-0.56, 0.63) | 0.24 | (-0.34, 0.81) | 0.59 | (-0.05, 1.22) | 0.304 | 0.070 |  |
|  | Model 3 | Yes | 0.18 | (-0.11, 0.48) | Ref (0.00) | 0.19 | (-0.19, 0.57) | 0.20 | (-0.26, 0.66) | 0.61 | **(0.30, 0.91)***** | 0.003 | < 0.001 | 0.199 |
|  |  | No | 0.32 | (-0.45, 1.10) | Ref (0.00) | -0.16 | (-0.74, 0.42) | 0.14 | (-0.42, 0.70) | 0.28 | (-0.37, 0.91) | 0.776 | 0.396 |  |
|  | Model 4 | Yes | 0.24 | (-0.07, 0.55) | Ref (0.00) | 0.14 | (-0.23, 0.52) | 0.11 | (-0.35, 0.58) | 0.45 | **(0.10, 0.80)*** | 0.170 | 0.013 | 0.205 |
|  |  | No | 0.42 | (-0.35, 1.19) | Ref (0.00) | -0.27 | (-0.84, 0.31) | -0.02 | (-0.62, 0.57) | -0.04 | (-0.73, 0.65) | 0.434 | 0.911 |  |
| Exercise |  |  |  |  |  |  |  |  |  |  |  |  |  |  |
|  | Model 1 | Yes | 0.27 | (-0.23, 0.76) | Ref (0.00) | 0.17 | (-0.36, 0.71) | 0.57 | (0.00, 1.14) | 1.02 | **(0.54, 1.51)***** | 0.001 | < 0.001 | 0.635 |
|  |  | No | 0.26 | (-0.19, 0.72) | Ref (0.00) | 0.36 | (-0.10, 0.81) | 0.51 | **(0.04, 0.98)*** | 1.30 | **(0.91, 1.70)***** | < 0.001 | < 0.001 |  |
|  | Model 2 | Yes | 0.39 | (-0.09, 0.86) | Ref (0.00) | 0.25 | (-0.24, 0.74) | 0.36 | (-0.20, 0.91) | 0.51 | **(0.08, 0.95)*** | 0.223 | 0.021 | 0.985 |
|  |  | No | 0.31 | (-0.07, 0.68) | Ref (0.00) | 0.15 | (-0.29, 0.59) | 0.31 | (-0.14, 0.76) | 0.91 | **(0.57, 1.25)***** | < 0.001 | < 0.001 |  |
|  | Model 3 | Yes | 0.29 | (-0.16, 0.75) | Ref (0.00) | 0.18 | (-0.31, 0.67) | 0.33 | (-0.20, 0.85) | 0.40 | (-0.01, 0.81) | 0.281 | 0.057 | 0.960 |
|  |  | No | 0.31 | (-0.05, 0.66) | Ref (0.00) | 0.10 | (-0.33, 0.52) | 0.20 | (-0.23, 0.64) | 0.60 | **(0.26, 0.94)***** | 0.020 | 0.001 |  |
|  | Model 4 | Yes | 0.37 | (-0.09, 0.83) | Ref (0.00) | 0.10 | (-0.38, 0.58) | 0.19 | (-0.32, 0.70) | 0.15 | (-0.28, 0.58) | 0.627 | 0.498 | 0.936 |
|  |  | No | 0.37 | (0.00, 0.75) | Ref (0.00) | 0.04 | (-0.38, 0.47) | 0.11 | (-0.35, 0.58) | 0.42 | **(0.02, 0.82)*** | 0.425 | 0.039 |  |
| Education |  |  |  |  |  |  |  |  |  |  |  |  |  |  |
|  | Model 1 | Above high school | 0.20 | (-0.24, 0.64) | Ref (0.00) | 0.17 | (-0.29, 0.62) | 0.43 | (-0.05, 0.91) | 1.13 | **(0.71, 1.56)***** | < 0.001 | < 0.001 | 0.899 |
|  |  | Others | 0.25 | (-0.30, 0.80) | Ref (0.00) | 0.40 | (-0.09, 0.90) | 0.61 | **(0.05, 1.16)*** | 1.06 | **(0.64, 1.49)***** | < 0.001 | < 0.001 |  |
|  | Model 2 | Above high school | 0.33 | (-0.08, 0.75) | Ref (0.00) | 0.12 | (-0.32, 0.56) | 0.25 | (-0.23, 0.73) | 0.79 | **(0.40, 1.19)***** | 0.001 | < 0.001 | 0.761 |
|  |  | Others | 0.34 | (-0.19, 0.87) | Ref (0.00) | 0.30 | (-0.19, 0.79) | 0.39 | (-0.15, 0.94) | 0.81 | **(0.42, 1.19)***** | 0.006 | < 0.001 |  |
|  | Model 3 | Above high school | 0.28 | (-0.11, 0.67) | Ref (0.00) | 0.07 | (-0.36, 0.49) | 0.18 | (-0.28, 0.64) | 0.56 | **(0.18, 0.94)**** | 0.039 | 0.005 | 0.958 |
|  |  | Others | 0.29 | (-0.23, 0.80) | Ref (0.00) | 0.18 | (-0.30, 0.66) | 0.28 | (-0.25, 0.80) | 0.52 | **(0.11, 0.93)*** | 0.121 | 0.015 |  |
|  | Model 4 | Above high school | 0.36 | (-0.04, 0.75) | Ref (0.00) | 0.01 | (-0.42, 0.43) | 0.07 | (-0.40, 0.53) | 0.35 | (-0.06, 0.76) | 0.720 | 0.092 | 0.985 |
|  |  | Others | 0.34 | (-0.18, 0.86) | Ref (0.00) | 0.12 | (-0.35, 0.60) | 0.19 | (-0.36, 0.75) | 0.36 | (-0.08, 0.80) | 0.551 | 0.108 |  |
| Income |  |  |  |  |  |  |  |  |  |  |  |  |  |  |
|  | Model 1 | ≤ $55,000 | 0.47 | **(0.01, 0.94)*** | Ref (0.00) | 0.51 | **(0.08, 0.94)*** | 0.41 | (-0.04, 0.86) | 1.38 | **(0.97, 1.79)***** | < 0.001 | < 0.001 | 0.810 |
|  |  | > $55,000 | -0.13 | (-0.62, 0.35) | Ref (0.00) | -0.06 | (-0.54, 0.43) | 0.54 | (-0.06, 1.14) | 0.81 | **(0.36, 1.26)***** | < 0.001 | 0.001 |  |
|  | Model 2 | ≤ $55,000 | 0.51 | **(0.11, 0.91)*** | Ref (0.00) | 0.30 | (-0.10, 0.70) | 0.14 | (-0.29, 0.56) | 0.92 | **(0.56, 1.28)***** | 0.001 | < 0.001 | 0.839 |
|  |  | > $55,000 | 0.06 | (-0.38, 0.50) | Ref (0.00) | -0.01 | (-0.49, 0.48) | 0.44 | (-0.14, 1.02) | 0.54 | **(0.06, 1.01)*** | 0.021 | 0.027 |  |
|  | Model 3 | ≤ $55,000 | 0.47 | **(0.09, 0.84)*** | Ref (0.00) | 0.21 | (-0.16, 0.59) | 0.04 | (-0.38, 0.46) | 0.69 | **(0.32, 1.06)***** | 0.032 | < 0.001 | 0.961 |
|  |  | > $55,000 | 0.00 | (-0.43, 0.42) | Ref (0.00) | -0.09 | (-0.57, 0.39) | 0.35 | (-0.20, 0.89) | 0.24 | (-0.23, 0.72) | 0.227 | 0.316 |  |
|  | Model 4 | ≤ $55,000 | 0.54 | **(0.15, 0.93)**** | Ref (0.00) | 0.15 | (-0.23, 0.54) | -0.06 | (-0.51, 0.38) | 0.49 | **(0.12, 0.86)**** | 0.597 | 0.010 | 0.975 |
|  |  | > $55,000 | 0.06 | (-0.37, 0.50) | Ref (0.00) | -0.14 | (-0.62, 0.34) | 0.24 | (-0.32, 0.80) | 0.04 | (-0.48, 0.56) | 0.908 | 0.874 |  |
| Sleep duration |  |  |  |  |  |  |  |  |  |  |  |  |  |  |
|  | Model 1 | ≤ 7 hours | 0.46 | (-0.06, 0.97) | Ref (0.00) | 0.55 | **(0.01, 1.10)*** | 0.98 | **(0.37, 1.59)**** | 1.55 | **(1.11, 1.99)***** | < 0.001 | < 0.001 | 0.413 |
|  |  | > 7 hours | 0.12 | (-0.35, 0.60) | Ref (0.00) | 0.05 | (-0.41, 0.51) | 0.22 | (-0.26, 0.69) | 1.04 | **(0.63, 1.45)***** | < 0.001 | < 0.001 |  |
|  | Model 2 | ≤ 7 hours | 0.46 | **(0.02, 0.90)*** | Ref (0.00) | 0.30 | (-0.19, 0.80) | 0.82 | **(0.28, 1.35)**** | 0.92 | **(0.49, 1.35)***** | 0.001 | < 0.001 | 0.419 |
|  |  | > 7 hours | 0.27 | (-0.17, 0.71) | Ref (0.00) | 0.06 | (-0.37, 0.49) | -0.05 | (-0.53, 0.42) | 0.68 | **(0.30, 1.07)***** | 0.008 | 0.001 |  |
|  | Model 3 | ≤ 7 hours | 0.38 | (-0.04, 0.80) | Ref (0.00) | 0.22 | (-0.26, 0.71) | 0.80 | **(0.29, 1.30)**** | 0.73 | **(0.30, 1.16)***** | 0.010 | 0.001 | 0.787 |
|  |  | > 7 hours | 0.25 | (-0.17, 0.67) | Ref (0.00) | -0.01 | (-0.42, 0.39) | -0.19 | (-0.67, 0.29) | 0.38 | (0.00, 0.75) | 0.312 | 0.052 |  |
|  | Model 4 | ≤ 7 hours | 0.43 | **(0.01, 0.86)*** | Ref (0.00) | 0.17 | (-0.33, 0.67) | 0.71 | **(0.20, 1.22)**** | 0.57 | **(0.11, 1.04)*** | 0.180 | 0.017 | 0.771 |
|  |  | > 7 hours | 0.32 | (-0.11, 0.75) | Ref (0.00) | -0.08 | (-0.48, 0.32) | -0.31 | (-0.80, 0.18) | 0.15 | (-0.23, 0.52) | 0.618 | 0.447 |  |
| Daily energy intake |  |  |  |  |  |  |  |  |  |  |  |  |  |  |
|  | Model 1 | Higher | 0.20 | (-0.27, 0.67) | Ref (0.00) | 0.50 | (-0.11, 1.11) | 0.96 | **(0.41, 1.52)***** | 1.21 | **(0.75, 1.67)***** | < 0.001 | < 0.001 | 0.053 |
|  |  | Lower | 0.32 | (-0.17, 0.81) | Ref (0.00) | 0.13 | (-0.25, 0.51) | 0.32 | (-0.14, 0.79) | 1.25 | **(0.89, 1.61)***** | < 0.001 | < 0.001 |  |
|  | Model 2 | Higher | 0.27 | (-0.13, 0.67) | Ref (0.00) | 0.40 | (-0.17, 0.97) | 0.47 | (-0.09, 1.02) | 0.60 | **(0.17, 1.04)**** | 0.025 | 0.007 | 0.073 |
|  |  | Lower | 0.43 | (0.00, 0.87) | Ref (0.00) | 0.02 | (-0.34, 0.38) | 0.18 | (-0.25, 0.61) | 0.78 | **(0.45, 1.11)***** | 0.001 | < 0.001 |  |
|  | Model 3 | Higher | 0.24 | (-0.13, 0.61) | Ref (0.00) | 0.38 | (-0.17, 0.93) | 0.35 | (-0.20, 0.89) | 0.46 | **(0.04, 0.88)*** | 0.122 | 0.034 | 0.067 |
|  |  | Lower | 0.28 | (-0.15, 0.71) | Ref (0.00) | -0.03 | (-0.38, 0.32) | 0.18 | (-0.23, 0.60) | 0.63 | **(0.31, 0.95)***** | 0.003 | < 0.001 |  |
|  | Model 4 | Higher | 0.31 | (-0.08, 0.70) | Ref (0.00) | 0.31 | (-0.24, 0.86) | 0.23 | (-0.30, 0.76) | 0.25 | (-0.21, 0.71) | 0.935 | 0.280 | 0.076 |
|  |  | Lower | 0.37 | (-0.06, 0.80) | Ref (0.00) | -0.11 | (-0.46, 0.25) | 0.04 | (-0.40, 0.48) | 0.34 | (-0.02, 0.69) | 0.488 | 0.064 |  |
| Healthy eating index-2015 |  |  |  |  |  |  |  |  |  |  |  |  |  |  |
|  | Model 1 | < 30.53 | 0.24 | (-0.28, 0.76) | Ref (0.00) | 0.11 | (-0.35, 0.58) | 0.36 | (-0.12, 0.84) | 1.23 | **(0.83, 1.63)***** | < 0.001 | < 0.001 | 0.626 |
|  |  | ≥ 30.53 | 0.21 | (-0.24, 0.66) | Ref (0.00) | 0.40 | (-0.08, 0.88) | 0.64 | **(0.13, 1.16)*** | 1.16 | **(0.77, 1.54)***** | < 0.001 | < 0.001 |  |
|  | Model 2 | < 30.53 | 0.38 | (-0.09, 0.84) | Ref (0.00) | -0.06 | (-0.50, 0.39) | 0.12 | (-0.36, 0.60) | 0.74 | **(0.36, 1.11)***** | 0.027 | < 0.001 | 0.624 |
|  |  | ≥ 30.53 | 0.27 | (-0.14, 0.69) | Ref (0.00) | 0.37 | (-0.06, 0.80) | 0.46 | (-0.01, 0.92) | 0.74 | **(0.36, 1.12)***** | 0.001 | < 0.001 |  |
|  | Model 3 | < 30.53 | 0.34 | (-0.12, 0.81) | Ref (0.00) | -0.07 | (-0.50, 0.36) | 0.09 | (-0.38, 0.55) | 0.58 | **(0.19, 0.97)**** | 0.110 | 0.004 | 0.254 |
|  |  | ≥ 30.53 | 0.21 | (-0.19, 0.61) | Ref (0.00) | 0.26 | (-0.16, 0.67) | 0.36 | (-0.10, 0.81) | 0.47 | **(0.12, 0.83)**** | 0.043 | 0.010 |  |
|  | Model 4 | < 30.53 | 0.38 | (-0.07, 0.84) | Ref (0.00) | -0.11 | (-0.54, 0.32) | 0.03 | (-0.47, 0.53) | 0.46 | **(0.02, 0.90)*** | 0.444 | 0.039 | 0.288 |
|  |  | ≥ 30.53 | 0.31 | (-0.11, 0.73) | Ref (0.00) | 0.18 | (-0.23, 0.59) | 0.21 | (-0.27, 0.68) | 0.20 | (-0.18, 0.57) | 0.950 | 0.297 |  |
| Dietary supplements use |  |  |  |  |  |  |  |  |  |  |  |  |  |  |
|  | Model 1 | Yes | 0.30 | (-0.23, 0.82) | Ref (0.00) | 0.20 | (-0.31, 0.71) | 0.54 | **(0.05, 1.03)*** | 1.25 | **(0.75, 1.76)***** | < 0.001 | < 0.001 | 0.961 |
|  |  | No | 0.07 | (-0.43, 0.56) | Ref (0.00) | 0.35 | (-0.11, 0.81) | 0.54 | **(0.02, 1.07)*** | 1.08 | **(0.76, 1.41)***** | < 0.001 | < 0.001 |  |
|  | Model 2 | Yes | 0.41 | (-0.05, 0.88) | Ref (0.00) | 0.12 | (-0.36, 0.61) | 0.36 | (-0.14, 0.85) | 0.82 | **(0.33, 1.32)***** | 0.009 | 0.001 | 0.923 |
|  |  | No | 0.17 | (-0.26, 0.60) | Ref (0.00) | 0.24 | (-0.18, 0.67) | 0.28 | (-0.22, 0.78) | 0.66 | **(0.37, 0.95)***** | 0.001 | < 0.001 |  |
|  | Model 3 | Yes | 0.40 | (-0.03, 0.83) | Ref (0.00) | 0.05 | (-0.41, 0.51) | 0.29 | (-0.18, 0.75) | 0.51 | **(0.03, 0.99)*** | 0.213 | 0.038 | 0.614 |
|  |  | No | 0.13 | (-0.29, 0.55) | Ref (0.00) | 0.22 | (-0.19, 0.62) | 0.20 | (-0.29, 0.70) | 0.58 | **(0.28, 0.88)***** | 0.006 | < 0.001 |  |
|  | Model 4 | Yes | 0.48 | **(0.03, 0.92)*** | Ref (0.00) | -0.02 | (-0.47, 0.44) | 0.17 | (-0.31, 0.65) | 0.30 | (-0.20, 0.81) | 0.962 | 0.232 | 0.536 |
|  |  | No | 0.20 | (-0.23, 0.62) | Ref (0.00) | 0.16 | (-0.25, 0.57) | 0.10 | (-0.40, 0.61) | 0.36 | **(0.04, 0.69)*** | 0.280 | 0.030 |  |
| Daily eating frequency |  |  |  |  |  |  |  |  |  |  |  |  |  |  |
|  | Model 1 | < 4.00 times | 0.82 | **(0.15, 1.50)*** | Ref (0.00) | 0.61 | **(0.05, 1.17)*** | 0.40 | (-0.06, 0.85) | 1.05 | **(0.63, 1.47)***** | < 0.001 | < 0.001 | 0.002 |
|  |  | ≥ 4.00 times | 0.16 | (-0.27, 0.58) | Ref (0.00) | -0.27 | (-0.70, 0.17) | 0.39 | (-0.26, 1.05) | 0.97 | **(0.39, 1.55)***** | 0.081 | 0.001 |  |
|  | Model 2 | < 4.00 times | 0.72 | **(0.07, 1.37)*** | Ref (0.00) | 0.41 | (-0.09, 0.91) | 0.15 | (-0.29, 0.59) | 0.65 | **(0.27, 1.02)***** | 0.051 | 0.001 | 0.008 |
|  |  | ≥ 4.00 times | 0.25 | (-0.12, 0.62) | Ref (0.00) | -0.18 | (-0.62, 0.25) | 0.33 | (-0.31, 0.97) | 0.80 | **(0.23, 1.37)**** | 0.267 | 0.006 |  |
|  | Model 3 | < 4.00 times | 0.57 | (-0.06, 1.19) | Ref (0.00) | 0.35 | (-0.14, 0.84) | 0.15 | (-0.28, 0.58) | 0.48 | **(0.11, 0.86)*** | 0.182 | 0.013 | 0.015 |
|  |  | ≥ 4.00 times | 0.18 | (-0.16, 0.53) | Ref (0.00) | -0.21 | (-0.63, 0.22) | 0.21 | (-0.41, 0.83) | 0.58 | **(0.06, 1.11)*** | 0.505 | 0.031 |  |

^a^The variables adjusted in each model were the factors mentioned above except the stratification variables.

^b^Data were listed as the weighted beta estimates and 95% confidence intervals, with *p < 0.05, **p < 0.01, ***p < 0.001.

^c^Q, quintile.

^d^Ref, reference.

^e^Tests for trends based on the variables containing the median values for each quartile.

^f^*P*_test_ was the result of Bonfreni correction.

^g^Multiplicative interaction was assessed by adding interaction terms to the models.

**Supplementary table 12.** Association of NFD with PA residual (years) stratified by variables of interest

|  |  |  | Nighttime fasting duration (hours) | | | | | | | | |  |  |  |
| --- | --- | --- | --- | --- | --- | --- | --- | --- | --- | --- | --- | --- | --- | --- |
|  |  |  | Q1^c^ | | Q2 | Q3 | | Q4 | | Q5 | | *P*_trend_^e^ | *P*_test_^f^ | *P*_interaction_^g^ |
|  |  |  | β^b^ | 95% CI | β | β | 95% CI | β | 95% CI | β | 95% CI |  |  |  |
| Age |  |  |  |  |  |  |  |  |  |  |  |  |  |  |
|  | Model 1^a^ | > 60 years | 0.27 | (-0.25, 0.79) | Ref (0.00)^d^ | -0.08 | (-0.47, 0.30) | 0.18 | (-0.21, 0.58) | 1.09 | **(0.74, 1.44)***** | < 0.001 | < 0.001 | < 0.001 |
|  |  | ≤ 60 years | 0.40 | **(0.11, 0.69)**** | Ref (0.00) | 0.16 | (-0.09, 0.41) | 0.50 | **(0.20, 0.80)***** | 0.93 | **(0.69, 1.17)***** | < 0.001 | < 0.001 |  |
|  | Model 2 | > 60 years | 0.48 | (-0.02, 0.98) | Ref (0.00) | -0.11 | (-0.47, 0.25) | 0.17 | (-0.22, 0.57) | 0.80 | **(0.45, 1.15)***** | 0.007 | < 0.001 | < 0.001 |
|  |  | ≤ 60 years | 0.44 | **(0.18, 0.70)***** | Ref (0.00) | 0.08 | (-0.14, 0.31) | 0.29 | **(0.03, 0.55)*** | 0.58 | **(0.39, 0.78)***** | 0.003 | < 0.001 |  |
|  | Model 3 | > 60 years | 0.39 | (-0.08, 0.86) | Ref (0.00) | -0.19 | (-0.54, 0.16) | 0.09 | (-0.30, 0.48) | 0.46 | **(0.08, 0.84)*** | 0.244 | 0.017 | < 0.001 |
|  |  | ≤ 60 years | 0.33 | **(0.07, 0.59)*** | Ref (0.00) | 0.06 | (-0.16, 0.28) | 0.28 | **(0.03, 0.54)*** | 0.50 | **(0.31, 0.69)***** | 0.005 | < 0.001 |  |
|  | Model 4 | > 60 years | 0.46 | (-0.01, 0.93) | Ref (0.00) | -0.23 | (-0.58, 0.11) | 0.02 | (-0.38, 0.41) | 0.33 | (-0.08, 0.73) | 0.774 | 0.114 | < 0.001 |
|  |  | ≤ 60 years | 0.40 | **(0.14, 0.66)**** | Ref (0.00) | 0.00 | (-0.22, 0.21) | 0.17 | (-0.07, 0.42) | 0.28 | **(0.10, 0.47)**** | 0.935 | 0.003 |  |
| Sex |  |  |  |  |  |  |  |  |  |  |  |  |  |  |
|  | Model 1 | Male | 0.33 | **(0.05, 0.61)*** | Ref (0.00) | 0.15 | (-0.15, 0.46) | 0.45 | **(0.13, 0.78)**** | 0.83 | **(0.59, 1.08)***** | < 0.001 | < 0.001 | 0.764 |
|  |  | Female | 0.32 | (-0.05, 0.69) | Ref (0.00) | -0.06 | (-0.30, 0.18) | 0.22 | (-0.12, 0.55) | 0.68 | **(0.44, 0.92)***** | < 0.001 | < 0.001 |  |
|  | Model 2 | Male | 0.38 | **(0.13, 0.63)**** | Ref (0.00) | 0.14 | (-0.14, 0.42) | 0.29 | (-0.01, 0.59) | 0.55 | **(0.31, 0.78)***** | 0.026 | < 0.001 | 0.160 |
|  |  | Female | 0.42 | **(0.09, 0.75)*** | Ref (0.00) | -0.18 | (-0.39, 0.03) | 0.04 | (-0.26, 0.34) | 0.27 | **(0.07, 0.47)**** | 0.643 | 0.010 |  |
|  | Model 3 | Male | 0.36 | **(0.11, 0.61)**** | Ref (0.00) | 0.09 | (-0.18, 0.35) | 0.24 | (-0.06, 0.53) | 0.35 | **(0.12, 0.58)**** | 0.434 | 0.003 | 0.165 |
|  |  | Female | 0.32 | (0.00, 0.64) | Ref (0.00) | -0.18 | (-0.39, 0.03) | 0.03 | (-0.27, 0.32) | 0.18 | (-0.04, 0.40) | 0.912 | 0.104 |  |
|  | Model 4 | Male | 0.39 | **(0.14, 0.64)**** | Ref (0.00) | 0.06 | (-0.20, 0.32) | 0.20 | (-0.11, 0.50) | 0.27 | **(0.03, 0.50)*** | 0.822 | 0.028 | 0.106 |
|  |  | Female | 0.39 | **(0.06, 0.71)*** | Ref (0.00) | -0.24 | **(-0.46, -0.03)*** | -0.08 | (-0.37, 0.21) | -0.02 | (-0.27, 0.22) | 0.007 | 0.844 |  |
| Race |  |  |  |  |  |  |  |  |  |  |  |  |  |  |
|  | Model 1 | Non-hispanic white | 0.30 | (0.00, 0.60) | Ref (0.00) | 0.06 | (-0.22, 0.33) | 0.25 | (-0.06, 0.56) | 0.80 | **(0.55, 1.05)***** | < 0.001 | < 0.001 | 0.417 |
|  |  | Others | 0.39 | **(0.11, 0.67)**** | Ref (0.00) | -0.02 | (-0.28, 0.23) | 0.49 | **(0.19, 0.79)**** | 0.63 | **(0.40, 0.85)***** | < 0.001 | < 0.001 |  |
|  | Model 2 | Non-hispanic white | 0.39 | **(0.13, 0.65)**** | Ref (0.00) | -0.02 | (-0.27, 0.24) | 0.11 | (-0.17, 0.40) | 0.46 | **(0.25, 0.67)***** | 0.102 | < 0.001 | 0.244 |
|  |  | Others | 0.36 | **(0.10, 0.63)**** | Ref (0.00) | -0.07 | (-0.31, 0.18) | 0.29 | **(0.02, 0.56)*** | 0.32 | **(0.11, 0.54)**** | 0.016 | 0.004 |  |
|  | Model 3 | Non-hispanic white | 0.33 | **(0.07, 0.58)*** | Ref (0.00) | -0.08 | (-0.33, 0.17) | 0.06 | (-0.22, 0.34) | 0.25 | **(0.04, 0.47)*** | 0.902 | 0.023 | 0.217 |
|  |  | Others | 0.35 | **(0.09, 0.61)**** | Ref (0.00) | -0.01 | (-0.26, 0.23) | 0.28 | **(0.02, 0.54)*** | 0.29 | **(0.08, 0.51)**** | 0.249 | 0.007 |  |
|  | Model 4 | Non-hispanic white | 0.37 | **(0.12, 0.62)**** | Ref (0.00) | -0.12 | (-0.37, 0.13) | -0.01 | (-0.29, 0.28) | 0.13 | (-0.09, 0.35) | 0.194 | 0.243 | 0.207 |
|  |  | Others | 0.41 | **(0.15, 0.68)**** | Ref (0.00) | -0.07 | (-0.32, 0.17) | 0.17 | (-0.09, 0.43) | 0.08 | (-0.17, 0.32) | 0.259 | 0.536 |  |
| BMI |  |  |  |  |  |  |  |  |  |  |  |  |  |  |
|  | Model 1 | < 30 kg/m^2^ | 0.32 | **(0.07, 0.58)*** | Ref (0.00) | 0.01 | (-0.23, 0.24) | 0.35 | **(0.09, 0.61)**** | 0.68 | **(0.47, 0.88)***** | < 0.001 | < 0.001 | 0.966 |
|  |  | ≥ 30 kg/m^2^ | 0.38 | **(0.01, 0.75)*** | Ref (0.00) | 0.09 | (-0.26, 0.43) | 0.03 | (-0.39, 0.45) | 0.56 | **(0.31, 0.80)***** | 0.029 | < 0.001 |  |
|  | Model 2 | < 30 kg/m^2^ | 0.33 | **(0.09, 0.57)**** | Ref (0.00) | -0.03 | (-0.27, 0.20) | 0.28 | **(0.02, 0.53)*** | 0.45 | **(0.25, 0.65)***** | 0.031 | < 0.001 | 0.926 |
|  |  | ≥ 30 kg/m^2^ | 0.41 | **(0.05, 0.77)*** | Ref (0.00) | 0.03 | (-0.31, 0.38) | -0.04 | (-0.45, 0.37) | 0.40 | **(0.15, 0.65)**** | 0.310 | 0.002 |  |
|  | Model 3 | < 30 kg/m^2^ | 0.30 | **(0.07, 0.54)*** | Ref (0.00) | -0.05 | (-0.28, 0.18) | 0.28 | **(0.03, 0.52)*** | 0.30 | **(0.12, 0.49)**** | 0.248 | 0.002 | 0.884 |
|  |  | ≥ 30 kg/m^2^ | 0.31 | (-0.04, 0.65) | Ref (0.00) | -0.02 | (-0.35, 0.30) | -0.14 | (-0.55, 0.26) | 0.25 | (-0.01, 0.51) | 0.803 | 0.058 |  |
|  | Model 4 | < 30 kg/m^2^ | 0.35 | **(0.11, 0.59)**** | Ref (0.00) | -0.09 | (-0.32, 0.14) | 0.21 | (-0.03, 0.45) | 0.18 | (-0.01, 0.36) | 0.604 | 0.056 | 0.876 |
|  |  | ≥ 30 kg/m^2^ | 0.38 | **(0.05, 0.72)*** | Ref (0.00) | -0.11 | (-0.43, 0.22) | -0.27 | (-0.68, 0.14) | 0.01 | (-0.29, 0.31) | 0.118 | 0.955 |  |
| Smoking |  |  |  |  |  |  |  |  |  |  |  |  |  |  |
|  | Model 1 | Yes | 0.34 | **(0.01, 0.68)*** | Ref (0.00) | 0.02 | (-0.28, 0.32) | 0.26 | (-0.11, 0.62) | 0.67 | **(0.40, 0.94)***** | 0.009 | < 0.001 | 0.562 |
|  |  | No | 0.15 | (-0.14, 0.44) | Ref (0.00) | 0.10 | (-0.17, 0.37) | 0.39 | **(0.13, 0.66)**** | 0.78 | **(0.52, 1.05)***** | < 0.001 | < 0.001 |  |
|  | Model 2 | Yes | 0.42 | **(0.12, 0.72)**** | Ref (0.00) | 0.04 | (-0.26, 0.34) | 0.13 | (-0.21, 0.47) | 0.39 | **(0.14, 0.65)**** | 0.462 | 0.003 | 0.845 |
|  |  | No | 0.25 | (-0.02, 0.51) | Ref (0.00) | -0.06 | (-0.29, 0.18) | 0.16 | (-0.10, 0.42) | 0.41 | **(0.18, 0.64)***** | 0.019 | 0.001 |  |
|  | Model 3 | Yes | 0.36 | **(0.07, 0.66)*** | Ref (0.00) | 0.00 | (-0.30, 0.30) | 0.09 | (-0.24, 0.42) | 0.21 | (-0.05, 0.47) | 0.813 | 0.106 | 0.524 |
|  |  | No | 0.22 | (-0.04, 0.48) | Ref (0.00) | -0.08 | (-0.32, 0.15) | 0.10 | (-0.16, 0.36) | 0.28 | **(0.04, 0.51)*** | 0.185 | 0.021 |  |
|  | Model 4 | Yes | 0.42 | **(0.13, 0.72)**** | Ref (0.00) | -0.05 | (-0.35, 0.24) | 0.00 | (-0.32, 0.33) | 0.04 | (-0.21, 0.30) | 0.082 | 0.743 | 0.588 |
|  |  | No | 0.25 | (-0.02, 0.51) | Ref (0.00) | -0.11 | (-0.34, 0.12) | 0.05 | (-0.22, 0.32) | 0.19 | (-0.07, 0.44) | 0.852 | 0.147 |  |
| Drinking |  |  |  |  |  |  |  |  |  |  |  |  |  |  |
|  | Model 1 | Yes | 0.36 | **(0.10, 0.62)**** | Ref (0.00) | 0.03 | (-0.21, 0.28) | 0.29 | (0.00, 0.58) | 0.84 | **(0.62, 1.06)***** | < 0.001 | < 0.001 | 0.528 |
|  |  | No | -0.07 | (-0.69, 0.55) | Ref (0.00) | -0.02 | (-0.36, 0.32) | 0.29 | (-0.09, 0.67) | 0.41 | **(0.01, 0.80)*** | 0.042 | 0.044 |  |
|  | Model 2 | Yes | 0.34 | **(0.12, 0.56)**** | Ref (0.00) | 0.01 | (-0.22, 0.23) | 0.15 | (-0.11, 0.40) | 0.48 | **(0.29, 0.68)***** | 0.014 | < 0.001 | 0.418 |
|  |  | No | 0.15 | (-0.40, 0.70) | Ref (0.00) | -0.08 | (-0.38, 0.22) | 0.13 | (-0.23, 0.48) | 0.09 | (-0.30, 0.48) | 0.919 | 0.646 |  |
|  | Model 3 | Yes | 0.30 | **(0.09, 0.52)**** | Ref (0.00) | -0.01 | (-0.24, 0.21) | 0.10 | (-0.16, 0.35) | 0.35 | **(0.16, 0.54)***** | 0.182 | < 0.001 | 0.373 |
|  |  | No | 0.12 | (-0.41, 0.64) | Ref (0.00) | -0.21 | (-0.50, 0.09) | 0.06 | (-0.29, 0.42) | -0.11 | (-0.50, 0.29) | 0.455 | 0.593 |  |
|  | Model 4 | Yes | 0.35 | **(0.14, 0.56)**** | Ref (0.00) | -0.05 | (-0.27, 0.17) | 0.03 | (-0.22, 0.28) | 0.22 | **(0.03, 0.42)*** | 0.751 | 0.026 | 0.385 |
|  |  | No | 0.18 | (-0.35, 0.71) | Ref (0.00) | -0.28 | (-0.57, 0.02) | -0.04 | (-0.41, 0.33) | -0.31 | (-0.70, 0.08) | 0.053 | 0.116 |  |
| Exercise |  |  |  |  |  |  |  |  |  |  |  |  |  |  |
|  | Model 1 | Yes | 0.19 | (-0.14, 0.52) | Ref (0.00) | -0.11 | (-0.47, 0.25) | 0.24 | (-0.07, 0.55) | 0.61 | **(0.32, 0.91)***** | 0.001 | < 0.001 | 0.287 |
|  |  | No | 0.46 | **(0.12, 0.80)**** | Ref (0.00) | 0.14 | (-0.11, 0.38) | 0.34 | **(0.06, 0.62)*** | 0.73 | **(0.49, 0.97)***** | < 0.001 | < 0.001 |  |
|  | Model 2 | Yes | 0.25 | (-0.05, 0.56) | Ref (0.00) | -0.05 | (-0.38, 0.27) | 0.11 | (-0.18, 0.40) | 0.28 | **(0.02, 0.54)*** | 0.438 | 0.033 | 0.502 |
|  |  | No | 0.49 | **(0.19, 0.78)**** | Ref (0.00) | -0.01 | (-0.25, 0.22) | 0.20 | (-0.06, 0.47) | 0.45 | **(0.24, 0.66)***** | 0.118 | < 0.001 |  |
|  | Model 3 | Yes | 0.17 | (-0.14, 0.47) | Ref (0.00) | -0.09 | (-0.41, 0.24) | 0.10 | (-0.18, 0.38) | 0.20 | (-0.06, 0.45) | 0.558 | 0.131 | 0.545 |
|  |  | No | 0.48 | **(0.20, 0.77)***** | Ref (0.00) | -0.03 | (-0.26, 0.20) | 0.16 | (-0.11, 0.42) | 0.28 | **(0.06, 0.50)*** | 0.969 | 0.014 |  |
|  | Model 4 | Yes | 0.20 | (-0.10, 0.50) | Ref (0.00) | -0.12 | (-0.43, 0.20) | 0.04 | (-0.23, 0.32) | 0.09 | (-0.17, 0.36) | 0.602 | 0.477 | 0.571 |
|  |  | No | 0.55 | **(0.25, 0.84)***** | Ref (0.00) | -0.08 | (-0.31, 0.15) | 0.07 | (-0.20, 0.34) | 0.11 | (-0.14, 0.35) | 0.123 | 0.375 |  |
| Education |  |  |  |  |  |  |  |  |  |  |  |  |  |  |
|  | Model 1 | Above high school | 0.16 | (-0.11, 0.42) | Ref (0.00) | -0.08 | (-0.35, 0.19) | 0.15 | (-0.14, 0.45) | 0.60 | **(0.33, 0.86)***** | < 0.001 | < 0.001 | 0.714 |
|  |  | Others | 0.51 | **(0.14, 0.89)**** | Ref (0.00) | 0.20 | (-0.10, 0.50) | 0.51 | **(0.15, 0.87)**** | 0.67 | **(0.41, 0.92)***** | 0.006 | < 0.001 |  |
|  | Model 2 | Above high school | 0.25 | (-0.01, 0.50) | Ref (0.00) | -0.10 | (-0.35, 0.14) | 0.04 | (-0.23, 0.30) | 0.35 | **(0.12, 0.59)**** | 0.160 | 0.003 | 0.834 |
|  |  | Others | 0.58 | **(0.21, 0.95)**** | Ref (0.00) | 0.13 | (-0.16, 0.41) | 0.36 | **(0.01, 0.70)*** | 0.47 | **(0.23, 0.71)***** | 0.237 | < 0.001 |  |
|  | Model 3 | Above high school | 0.21 | (-0.04, 0.46) | Ref (0.00) | -0.13 | (-0.37, 0.12) | -0.01 | (-0.27, 0.26) | 0.20 | (-0.02, 0.42) | 0.789 | 0.074 | 0.628 |
|  |  | Others | 0.54 | **(0.19, 0.90)**** | Ref (0.00) | 0.06 | (-0.22, 0.34) | 0.30 | (-0.03, 0.63) | 0.33 | **(0.08, 0.59)*** | 0.805 | 0.011 |  |
|  | Model 4 | Above high school | 0.25 | (0.00, 0.49) | Ref (0.00) | -0.16 | (-0.39, 0.08) | -0.06 | (-0.32, 0.19) | 0.09 | (-0.13, 0.32) | 0.276 | 0.413 | 0.596 |
|  |  | Others | 0.60 | **(0.25, 0.95)***** | Ref (0.00) | 0.00 | (-0.28, 0.28) | 0.21 | (-0.13, 0.55) | 0.16 | (-0.10, 0.42) | 0.211 | 0.219 |  |
| Income |  |  |  |  |  |  |  |  |  |  |  |  |  |  |
|  | Model 1 | ≤ $55,000 | 0.39 | **(0.09, 0.69)*** | Ref (0.00) | 0.26 | **(0.01, 0.51)*** | 0.34 | **(0.06, 0.62)*** | 0.70 | **(0.47, 0.93)***** | < 0.001 | < 0.001 | 0.805 |
|  |  | > $55,000 | 0.08 | (-0.27, 0.43) | Ref (0.00) | -0.25 | (-0.56, 0.05) | 0.13 | (-0.26, 0.52) | 0.54 | **(0.23, 0.85)***** | 0.004 | 0.001 |  |
|  | Model 2 | ≤ $55,000 | 0.42 | **(0.15, 0.70)**** | Ref (0.00) | 0.11 | (-0.10, 0.33) | 0.15 | (-0.11, 0.41) | 0.37 | **(0.17, 0.58)***** | 0.249 | 0.001 | 0.863 |
|  |  | > $55,000 | 0.22 | (-0.08, 0.52) | Ref (0.00) | -0.20 | (-0.49, 0.08) | 0.07 | (-0.29, 0.44) | 0.35 | **(0.05, 0.65)*** | 0.205 | 0.021 |  |
|  | Model 3 | ≤ $55,000 | 0.38 | **(0.11, 0.65)**** | Ref (0.00) | 0.07 | (-0.14, 0.28) | 0.11 | (-0.15, 0.37) | 0.26 | **(0.04, 0.48)*** | 0.819 | 0.020 | 0.980 |
|  |  | > $55,000 | 0.17 | (-0.13, 0.47) | Ref (0.00) | -0.24 | (-0.52, 0.04) | 0.02 | (-0.33, 0.37) | 0.15 | (-0.15, 0.44) | 0.922 | 0.330 |  |
|  | Model 4 | ≤ $55,000 | 0.43 | **(0.17, 0.70)**** | Ref (0.00) | 0.03 | (-0.18, 0.23) | 0.04 | (-0.23, 0.30) | 0.12 | (-0.10, 0.35) | 0.185 | 0.284 | 0.956 |
|  |  | > $55,000 | 0.21 | (-0.09, 0.51) | Ref (0.00) | -0.28 | (-0.56, 0.00) | -0.04 | (-0.40, 0.32) | 0.03 | (-0.28, 0.33) | 0.345 | 0.852 |  |
| Sleep duration |  |  |  |  |  |  |  |  |  |  |  |  |  |  |
|  | Model 1 | ≤ 7 hours | 0.51 | **(0.15, 0.87)**** | Ref (0.00) | 0.14 | (-0.20, 0.49) | 0.45 | **(0.08, 0.83)*** | 0.92 | **(0.63, 1.21)***** | 0.002 | < 0.001 | 0.258 |
|  |  | > 7 hours | 0.15 | (-0.14, 0.43) | Ref (0.00) | -0.04 | (-0.34, 0.26) | 0.23 | (-0.05, 0.50) | 0.62 | **(0.36, 0.88)***** | < 0.001 | < 0.001 |  |
|  | Model 2 | ≤ 7 hours | 0.52 | **(0.21, 0.83)***** | Ref (0.00) | -0.03 | (-0.36, 0.29) | 0.33 | **(0.01, 0.65)*** | 0.47 | **(0.20, 0.73)***** | 0.438 | 0.001 | 0.192 |
|  |  | > 7 hours | 0.25 | (0.00, 0.50) | Ref (0.00) | -0.03 | (-0.29, 0.23) | 0.03 | (-0.23, 0.30) | 0.35 | **(0.12, 0.58)**** | 0.090 | 0.004 |  |
|  | Model 3 | ≤ 7 hours | 0.46 | **(0.16, 0.77)**** | Ref (0.00) | -0.07 | (-0.39, 0.26) | 0.32 | **(0.01, 0.62)*** | 0.33 | **(0.07, 0.59)*** | 0.910 | 0.015 | 0.485 |
|  |  | > 7 hours | 0.22 | (-0.02, 0.46) | Ref (0.00) | -0.06 | (-0.31, 0.18) | -0.02 | (-0.28, 0.24) | 0.19 | (-0.04, 0.43) | 0.618 | 0.103 |  |
|  | Model 4 | ≤ 7 hours | 0.52 | **(0.21, 0.82)***** | Ref (0.00) | -0.12 | (-0.45, 0.21) | 0.23 | (-0.06, 0.53) | 0.18 | (-0.11, 0.46) | 0.204 | 0.223 | 0.466 |
|  |  | > 7 hours | 0.26 | **(0.02, 0.50)*** | Ref (0.00) | -0.10 | (-0.34, 0.14) | -0.09 | (-0.35, 0.18) | 0.07 | (-0.16, 0.29) | 0.387 | 0.555 |  |
| Daily energy intake |  |  |  |  |  |  |  |  |  |  |  |  |  |  |
|  | Model 1 | Higher | 0.22 | (-0.07, 0.52) | Ref (0.00) | 0.02 | (-0.35, 0.39) | 0.55 | **(0.18, 0.92)**** | 0.68 | **(0.38, 0.99)***** | 0.002 | < 0.001 | 0.010 |
|  |  | Lower | 0.40 | **(0.05, 0.74)*** | Ref (0.00) | 0.05 | (-0.20, 0.30) | 0.21 | (-0.08, 0.50) | 0.78 | **(0.56, 1.00)***** | < 0.001 | < 0.001 |  |
|  | Model 2 | Higher | 0.27 | **(0.03, 0.52)*** | Ref (0.00) | -0.07 | (-0.40, 0.26) | 0.18 | (-0.15, 0.51) | 0.22 | (-0.04, 0.48) | 0.780 | 0.091 | 0.023 |
|  |  | Lower | 0.47 | **(0.16, 0.78)**** | Ref (0.00) | -0.02 | (-0.25, 0.20) | 0.12 | (-0.15, 0.38) | 0.45 | **(0.25, 0.65)***** | 0.042 | < 0.001 |  |
|  | Model 3 | Higher | 0.25 | **(0.02, 0.48)*** | Ref (0.00) | -0.08 | (-0.40, 0.24) | 0.11 | (-0.23, 0.45) | 0.11 | (-0.14, 0.36) | 0.338 | 0.397 | 0.019 |
|  |  | Lower | 0.37 | **(0.06, 0.68)*** | Ref (0.00) | -0.04 | (-0.26, 0.17) | 0.13 | (-0.13, 0.38) | 0.34 | **(0.15, 0.53)***** | 0.096 | 0.001 |  |
|  | Model 4 | Higher | 0.27 | **(0.04, 0.51)*** | Ref (0.00) | -0.10 | (-0.42, 0.22) | 0.07 | (-0.26, 0.40) | 0.04 | (-0.21, 0.30) | 0.107 | 0.742 | 0.022 |
|  |  | Lower | 0.44 | **(0.13, 0.75)**** | Ref (0.00) | -0.11 | (-0.33, 0.11) | 0.01 | (-0.25, 0.27) | 0.10 | (-0.10, 0.31) | 0.257 | 0.326 |  |
| Healthy eating index-2015 |  |  |  |  |  |  |  |  |  |  |  |  |  |  |
|  | Model 1 | < 30.53 | 0.21 | (-0.14, 0.55) | Ref (0.00) | -0.01 | (-0.29, 0.27) | 0.31 | (-0.02, 0.63) | 0.68 | **(0.43, 0.93)***** | < 0.001 | < 0.001 | 0.379 |
|  |  | ≥ 30.53 | 0.39 | **(0.10, 0.68)**** | Ref (0.00) | 0.08 | (-0.23, 0.38) | 0.29 | (0.00, 0.58) | 0.69 | **(0.44, 0.94)***** | 0.001 | < 0.001 |  |
|  | Model 2 | < 30.53 | 0.30 | (-0.01, 0.60) | Ref (0.00) | -0.12 | (-0.38, 0.13) | 0.14 | (-0.15, 0.44) | 0.33 | **(0.12, 0.55)**** | 0.210 | 0.003 | 0.689 |
|  |  | ≥ 30.53 | 0.43 | **(0.17, 0.69)***** | Ref (0.00) | 0.05 | (-0.22, 0.32) | 0.15 | (-0.11, 0.41) | 0.40 | **(0.16, 0.63)***** | 0.340 | 0.001 |  |
|  | Model 3 | < 30.53 | 0.26 | (-0.03, 0.56) | Ref (0.00) | -0.11 | (-0.36, 0.13) | 0.15 | (-0.13, 0.43) | 0.27 | **(0.03, 0.50)*** | 0.361 | 0.027 | 0.653 |
|  |  | ≥ 30.53 | 0.37 | **(0.13, 0.62)**** | Ref (0.00) | 0.00 | (-0.27, 0.26) | 0.10 | (-0.16, 0.36) | 0.24 | **(0.01, 0.48)*** | 0.981 | 0.041 |  |
|  | Model 4 | < 30.53 | 0.30 | **(0.01, 0.60)*** | Ref (0.00) | -0.15 | (-0.39, 0.09) | 0.10 | (-0.19, 0.39) | 0.16 | (-0.08, 0.39) | 0.785 | 0.195 | 0.698 |
|  |  | ≥ 30.53 | 0.43 | **(0.19, 0.68)***** | Ref (0.00) | -0.05 | (-0.31, 0.21) | 0.01 | (-0.25, 0.26) | 0.07 | (-0.17, 0.30) | 0.065 | 0.579 |  |
| Dietary supplements use |  |  |  |  |  |  |  |  |  |  |  |  |  |  |
|  | Model 1 | Yes | 0.32 | (-0.01, 0.66) | Ref (0.00) | -0.10 | (-0.39, 0.18) | 0.25 | (-0.04, 0.54) | 0.73 | **(0.44, 1.01)***** | < 0.001 | < 0.001 | 0.964 |
|  |  | No | 0.26 | (-0.07, 0.59) | Ref (0.00) | 0.20 | (-0.11, 0.52) | 0.39 | **(0.04, 0.75)*** | 0.64 | **(0.40, 0.88)***** | < 0.001 | < 0.001 |  |
|  | Model 2 | Yes | 0.41 | **(0.11, 0.71)**** | Ref (0.00) | -0.16 | (-0.43, 0.11) | 0.11 | (-0.16, 0.39) | 0.42 | **(0.15, 0.69)**** | 0.285 | 0.003 | 0.941 |
|  |  | No | 0.31 | **(0.03, 0.60)*** | Ref (0.00) | 0.13 | (-0.15, 0.40) | 0.20 | (-0.13, 0.53) | 0.33 | **(0.12, 0.54)**** | 0.186 | 0.003 |  |
|  | Model 3 | Yes | 0.39 | **(0.10, 0.69)**** | Ref (0.00) | -0.18 | (-0.43, 0.07) | 0.10 | (-0.17, 0.37) | 0.24 | (-0.03, 0.50) | 0.852 | 0.079 | 0.605 |
|  |  | No | 0.27 | (-0.01, 0.55) | Ref (0.00) | 0.12 | (-0.15, 0.38) | 0.15 | (-0.17, 0.48) | 0.27 | **(0.06, 0.49)*** | 0.299 | 0.013 |  |
|  | Model 4 | Yes | 0.45 | **(0.17, 0.74)**** | Ref (0.00) | -0.23 | (-0.49, 0.02) | 0.01 | (-0.27, 0.28) | 0.08 | (-0.21, 0.36) | 0.083 | 0.595 | 0.504 |
|  |  | No | 0.31 | **(0.02, 0.59)*** | Ref (0.00) | 0.08 | (-0.18, 0.35) | 0.10 | (-0.23, 0.43) | 0.16 | (-0.05, 0.37) | 0.733 | 0.133 |  |
| Daily eating frequency |  |  |  |  |  |  |  |  |  |  |  |  |  |  |
|  | Model 1 | < 4.00 times | 0.92 | **(0.55, 1.28)***** | Ref (0.00) | 0.21 | (-0.09, 0.51) | 0.24 | (-0.08, 0.56) | 0.58 | **(0.33, 0.84)***** | 0.019 | < 0.001 | < 0.001 |
|  |  | ≥ 4.00 times | 0.19 | (-0.07, 0.45) | Ref (0.00) | -0.27 | **(-0.52, -0.02)*** | 0.17 | (-0.18, 0.52) | 0.53 | **(0.15, 0.92)**** | 0.681 | 0.008 |  |
|  | Model 2 | < 4.00 times | 0.84 | **(0.52, 1.17)***** | Ref (0.00) | 0.08 | (-0.19, 0.34) | 0.07 | (-0.22, 0.37) | 0.29 | **(0.05, 0.53)*** | 0.924 | 0.017 | 0.001 |
|  |  | ≥ 4.00 times | 0.26 | **(0.04, 0.48)*** | Ref (0.00) | -0.21 | (-0.47, 0.04) | 0.13 | (-0.20, 0.45) | 0.42 | **(0.06, 0.77)*** | 0.634 | 0.022 |  |
|  | Model 3 | < 4.00 times | 0.75 | **(0.42, 1.07)***** | Ref (0.00) | 0.05 | (-0.22, 0.32) | 0.09 | (-0.20, 0.38) | 0.19 | (-0.05, 0.43) | 0.586 | 0.111 | 0.001 |
|  |  | ≥ 4.00 times | 0.20 | (-0.01, 0.40) | Ref (0.00) | -0.21 | (-0.45, 0.03) | 0.06 | (-0.26, 0.37) | 0.28 | (-0.03, 0.59) | 0.428 | 0.074 |  |

^a^The variables adjusted in each model were the factors mentioned above except the stratification variables.

^b^Data were listed as the weighted beta estimates and 95% confidence intervals, with *p < 0.05, **p < 0.01, ***p < 0.001.

^c^Q, quintile.

^d^Ref, reference.

^e^Tests for trends based on the variables containing the median values for each quartile.

^f^*P*_test_ was the result of Bonfreni correction.

^g^Multiplicative interaction was assessed by adding interaction terms to the models.

**Supplementary table 13.** Association of NFD with AL stratified by variables of interest

|  |  |  | Nighttime fasting duration (hours) | | | | | | | | |  |  |  |
| --- | --- | --- | --- | --- | --- | --- | --- | --- | --- | --- | --- | --- | --- | --- |
|  |  |  | Q1^c^ | | Q2 | Q3 | | Q4 | | Q5 | | *P*_trend_^e^ | *P*_test_^f^ | *P*_interaction_^g^ |
|  |  |  | β^b^ | 95% CI | β | β | 95% CI | β | 95% CI | β | 95% CI |  |  |  |
| Age |  |  |  |  |  |  |  |  |  |  |  |  |  |  |
|  | Model 1^a^ | > 60 years | 0.01 | (-0.01, 0.02) | Ref (0.00)^d^ | 0.00 | (-0.01, 0.02) | 0.01 | (-0.01, 0.02) | 0.04 | **(0.02, 0.05)***** | < 0.001 | < 0.001 | 0.030 |
|  |  | ≤ 60 years | 0.01 | (0.00, 0.02) | Ref (0.00) | 0.00 | (-0.01, 0.01) | 0.00 | (-0.01, 0.01) | 0.01 | (0.00, 0.02) | 0.422 | 0.031 |  |
|  | Model 2 | > 60 years | 0.01 | (-0.01, 0.03) | Ref (0.00) | 0.00 | (-0.01, 0.01) | 0.01 | (-0.01, 0.02) | 0.03 | **(0.01, 0.04)***** | 0.003 | < 0.001 | 0.009 |
|  |  | ≤ 60 years | 0.01 | (0.00, 0.02) | Ref (0.00) | 0.00 | (-0.01, 0.01) | 0.00 | (-0.01, 0.01) | 0.00 | (-0.01, 0.01) | 0.044 | 0.890 |  |
|  | Model 3 | > 60 years | 0.01 | (-0.01, 0.03) | Ref (0.00) | 0.00 | (-0.01, 0.01) | 0.00 | (-0.01, 0.02) | 0.02 | (0.00, 0.03) | 0.106 | 0.010 | 0.008 |
|  |  | ≤ 60 years | 0.01 | (0.00, 0.02) | Ref (0.00) | 0.00 | (-0.01, 0.01) | -0.01 | (-0.01, 0.00) | 0.00 | (-0.01, 0.01) | 0.014 | 0.608 |  |
|  | Model 4 | > 60 years | 0.01 | (0.00, 0.03) | Ref (0.00) | 0.00 | (-0.02, 0.01) | 0.00 | (-0.02, 0.02) | 0.01 | (0.00, 0.03) | 0.567 | 0.101 | 0.014 |
|  |  | ≤ 60 years | 0.01 | (0.00, 0.02) | Ref (0.00) | 0.00 | (-0.01, 0.01) | -0.01 | (-0.02, 0.00) | 0.00 | (-0.01, 0.00) | 0.002 | 0.244 |  |
| Sex |  |  |  |  |  |  |  |  |  |  |  |  |  |  |
|  | Model 1 | Male | 0.01 | (0.00, 0.02) | Ref (0.00) | 0.01 | (0.00, 0.02) | 0.01 | (-0.01, 0.02) | 0.03 | **(0.02, 0.04)***** | < 0.001 | < 0.001 | 0.640 |
|  |  | Female | 0.01 | (0.00, 0.02) | Ref (0.00) | 0.00 | (-0.01, 0.01) | 0.01 | (0.00, 0.02) | 0.03 | **(0.02, 0.03)***** | < 0.001 | < 0.001 |  |
|  | Model 2 | Male | 0.01 | (0.00, 0.02) | Ref (0.00) | 0.01 | (0.00, 0.02) | 0.00 | (-0.01, 0.02) | 0.02 | **(0.01, 0.03)***** | 0.019 | < 0.001 | 0.248 |
|  |  | Female | 0.01 | (0.00, 0.02) | Ref (0.00) | 0.00 | (-0.01, 0.01) | 0.00 | (-0.01, 0.01) | 0.01 | **(0.01, 0.02)**** | 0.088 | 0.002 |  |
|  | Model 3 | Male | 0.01 | (0.00, 0.02) | Ref (0.00) | 0.01 | (-0.01, 0.02) | 0.00 | (-0.01, 0.01) | 0.02 | **(0.01, 0.02)***** | 0.221 | 0.001 | 0.323 |
|  |  | Female | 0.01 | (0.00, 0.02) | Ref (0.00) | 0.00 | (-0.01, 0.01) | 0.00 | (-0.01, 0.01) | 0.01 | (0.00, 0.02) | 0.209 | 0.011 |  |
|  | Model 4 | Male | 0.01 | **(0.01, 0.02)***** | Ref (0.00) | 0.00 | (-0.01, 0.01) | 0.00 | (-0.01, 0.01) | 0.01 | (0.00, 0.02) | 0.460 | 0.071 | 0.218 |
|  |  | Female | 0.01 | (0.00, 0.02) | Ref (0.00) | 0.00 | (-0.01, 0.01) | 0.00 | (-0.01, 0.01) | 0.01 | (0.00, 0.02) | 0.968 | 0.144 |  |
| Race |  |  |  |  |  |  |  |  |  |  |  |  |  |  |
|  | Model 1 | Non-hispanic white | 0.01 | (0.00, 0.02) | Ref (0.00) | 0.01 | (0.00, 0.01) | 0.01 | (-0.01, 0.02) | 0.03 | **(0.02, 0.04)***** | < 0.001 | < 0.001 | 0.989 |
|  |  | Others | 0.01 | **(0.01, 0.02)***** | Ref (0.00) | 0.00 | (-0.01, 0.01) | 0.01 | (0.00, 0.02) | 0.02 | **(0.01, 0.02)***** | 0.023 | < 0.001 |  |
|  | Model 2 | Non-hispanic white | 0.01 | (0.00, 0.02) | Ref (0.00) | 0.00 | (-0.01, 0.01) | 0.00 | (-0.01, 0.01) | 0.02 | **(0.01, 0.03)***** | 0.004 | < 0.001 | 0.794 |
|  |  | Others | 0.01 | **(0.01, 0.02)***** | Ref (0.00) | 0.00 | (-0.01, 0.01) | 0.00 | (-0.01, 0.01) | 0.01 | (0.00, 0.01) | 0.975 | 0.041 |  |
|  | Model 3 | Non-hispanic white | 0.01 | (0.00, 0.02) | Ref (0.00) | 0.00 | (-0.01, 0.01) | 0.00 | (-0.01, 0.01) | 0.01 | **(0.01, 0.02)***** | 0.070 | < 0.001 | 0.752 |
|  |  | Others | 0.01 | (0.00, 0.02) | Ref (0.00) | 0.00 | (-0.01, 0.01) | 0.00 | (0.00, 0.01) | 0.01 | (0.00, 0.01) | 0.887 | 0.047 |  |
|  | Model 4 | Non-hispanic white | 0.01 | (0.00, 0.02) | Ref (0.00) | 0.00 | (-0.01, 0.01) | 0.00 | (-0.01, 0.01) | 0.01 | (0.00, 0.02) | 0.957 | 0.015 | 0.716 |
|  |  | Others | 0.01 | **(0.01, 0.02)***** | Ref (0.00) | 0.00 | (-0.01, 0.01) | 0.00 | (-0.01, 0.01) | 0.00 | (-0.01, 0.01) | 0.072 | 0.543 |  |
| BMI |  |  |  |  |  |  |  |  |  |  |  |  |  |  |
|  | Model 1 | < 30 kg/m^2^ | 0.01 | (0.00, 0.02) | Ref (0.00) | 0.00 | (-0.01, 0.01) | 0.01 | (0.00, 0.02) | 0.02 | **(0.02, 0.03)***** | < 0.001 | < 0.001 | 0.639 |
|  |  | ≥ 30 kg/m^2^ | 0.01 | (0.00, 0.02) | Ref (0.00) | 0.01 | (0.00, 0.03) | 0.00 | (-0.01, 0.01) | 0.02 | **(0.01, 0.03)***** | 0.017 | < 0.001 |  |
|  | Model 2 | < 30 kg/m^2^ | 0.01 | (0.00, 0.02) | Ref (0.00) | 0.00 | (-0.01, 0.01) | 0.01 | (0.00, 0.02) | 0.02 | **(0.01, 0.03)***** | 0.003 | < 0.001 | 0.522 |
|  |  | ≥ 30 kg/m^2^ | 0.01 | (0.00, 0.03) | Ref (0.00) | 0.01 | (0.00, 0.02) | 0.00 | (-0.01, 0.01) | 0.02 | **(0.01, 0.02)***** | 0.207 | 0.001 |  |
|  | Model 3 | < 30 kg/m^2^ | 0.01 | (0.00, 0.02) | Ref (0.00) | 0.00 | (-0.01, 0.00) | 0.01 | (0.00, 0.02) | 0.02 | **(0.01, 0.02)***** | 0.028 | < 0.001 | 0.707 |
|  |  | ≥ 30 kg/m^2^ | 0.01 | (0.00, 0.02) | Ref (0.00) | 0.01 | (0.00, 0.02) | -0.01 | (-0.02, 0.01) | 0.01 | (0.00, 0.02) | 0.406 | 0.006 |  |
|  | Model 4 | < 30 kg/m^2^ | 0.01 | (0.00, 0.02) | Ref (0.00) | 0.00 | (-0.01, 0.00) | 0.00 | (-0.01, 0.01) | 0.01 | (0.00, 0.02) | 0.823 | 0.025 | 0.715 |
|  |  | ≥ 30 kg/m^2^ | 0.01 | (0.00, 0.03) | Ref (0.00) | 0.01 | (0.00, 0.02) | -0.01 | (-0.02, 0.00) | 0.00 | (-0.01, 0.01) | 0.411 | 0.416 |  |
| Smoking |  |  |  |  |  |  |  |  |  |  |  |  |  |  |
|  | Model 1 | Yes | 0.01 | (0.00, 0.02) | Ref (0.00) | 0.00 | (-0.01, 0.01) | 0.01 | (-0.01, 0.02) | 0.03 | **(0.02, 0.03)***** | < 0.001 | < 0.001 | 0.126 |
|  |  | No | 0.01 | (0.00, 0.02) | Ref (0.00) | 0.01 | (0.00, 0.02) | 0.01 | (0.00, 0.02) | 0.03 | **(0.02, 0.04)***** | < 0.001 | < 0.001 |  |
|  | Model 2 | Yes | 0.01 | (0.00, 0.02) | Ref (0.00) | 0.00 | (-0.01, 0.01) | 0.00 | (-0.01, 0.02) | 0.02 | **(0.01, 0.03)***** | 0.064 | < 0.001 | 0.494 |
|  |  | No | 0.01 | (0.00, 0.02) | Ref (0.00) | 0.00 | (0.00, 0.01) | 0.00 | (-0.01, 0.01) | 0.02 | **(0.01, 0.02)***** | 0.018 | 0.001 |  |
|  | Model 3 | Yes | 0.01 | (0.00, 0.02) | Ref (0.00) | 0.00 | (-0.01, 0.01) | 0.00 | (-0.01, 0.02) | 0.01 | (0.00, 0.02) | 0.292 | 0.008 | 0.645 |
|  |  | No | 0.01 | (0.00, 0.02) | Ref (0.00) | 0.00 | (-0.01, 0.01) | 0.00 | (-0.01, 0.01) | 0.01 | (0.00, 0.02) | 0.121 | 0.005 |  |
|  | Model 4 | Yes | 0.01 | (0.00, 0.02) | Ref (0.00) | 0.00 | (-0.01, 0.01) | 0.00 | (-0.01, 0.01) | 0.01 | (0.00, 0.02) | 0.545 | 0.183 | 0.555 |
|  |  | No | 0.01 | (0.00, 0.02) | Ref (0.00) | 0.00 | (-0.01, 0.01) | 0.00 | (-0.01, 0.01) | 0.01 | (0.00, 0.02) | 0.840 | 0.095 |  |
| Drinking |  |  |  |  |  |  |  |  |  |  |  |  |  |  |
|  | Model 1 | Yes | 0.01 | (0.00, 0.02) | Ref (0.00) | 0.01 | (0.00, 0.01) | 0.01 | (0.00, 0.02) | 0.03 | **(0.02, 0.04)***** | < 0.001 | < 0.001 | 0.989 |
|  |  | No | 0.01 | (-0.01, 0.03) | Ref (0.00) | 0.01 | (-0.01, 0.02) | 0.00 | (-0.02, 0.02) | 0.02 | **(0.01, 0.04)***** | 0.014 | 0.001 |  |
|  | Model 2 | Yes | 0.01 | (0.00, 0.02) | Ref (0.00) | 0.00 | (0.00, 0.01) | 0.01 | (-0.01, 0.01) | 0.02 | **(0.01, 0.02)***** | 0.002 | < 0.001 | 0.778 |
|  |  | No | 0.01 | (-0.01, 0.03) | Ref (0.00) | 0.00 | (-0.01, 0.01) | 0.00 | (-0.02, 0.01) | 0.01 | (0.00, 0.03) | 0.548 | 0.058 |  |
|  | Model 3 | Yes | 0.01 | (0.00, 0.02) | Ref (0.00) | 0.00 | (0.00, 0.01) | 0.00 | (-0.01, 0.01) | 0.01 | **(0.01, 0.02)***** | 0.021 | < 0.001 | 0.786 |
|  |  | No | 0.01 | (-0.01, 0.03) | Ref (0.00) | 0.00 | (-0.01, 0.01) | -0.01 | (-0.02, 0.01) | 0.01 | (0.00, 0.02) | 0.801 | 0.186 |  |
|  | Model 4 | Yes | 0.01 | (0.00, 0.02) | Ref (0.00) | 0.00 | (-0.01, 0.01) | 0.00 | (-0.01, 0.01) | 0.01 | (0.00, 0.02) | 0.727 | 0.011 | 0.808 |
|  |  | No | 0.01 | (0.00, 0.03) | Ref (0.00) | 0.00 | (-0.01, 0.01) | -0.01 | (-0.02, 0.01) | 0.00 | (-0.01, 0.02) | 0.365 | 0.777 |  |
| Exercise |  |  |  |  |  |  |  |  |  |  |  |  |  |  |
|  | Model 1 | Yes | 0.01 | (-0.01, 0.02) | Ref (0.00) | 0.00 | (-0.01, 0.01) | 0.01 | (-0.01, 0.02) | 0.03 | **(0.02, 0.04)***** | 0.001 | < 0.001 | 0.195 |
|  |  | No | 0.02 | **(0.01, 0.02)**** | Ref (0.00) | 0.01 | (0.00, 0.02) | 0.01 | (0.00, 0.02) | 0.02 | **(0.02, 0.03)***** | < 0.001 | < 0.001 |  |
|  | Model 2 | Yes | 0.01 | (0.00, 0.02) | Ref (0.00) | 0.00 | (-0.01, 0.01) | 0.00 | (-0.01, 0.02) | 0.02 | **(0.01, 0.03)**** | 0.062 | 0.003 | 0.310 |
|  |  | No | 0.02 | **(0.01, 0.02)***** | Ref (0.00) | 0.00 | (0.00, 0.01) | 0.00 | (-0.01, 0.01) | 0.02 | **(0.01, 0.02)***** | 0.062 | < 0.001 |  |
|  | Model 3 | Yes | 0.01 | (0.00, 0.02) | Ref (0.00) | 0.00 | (-0.01, 0.01) | 0.00 | (-0.01, 0.01) | 0.01 | **(0.01, 0.02)**** | 0.068 | 0.004 | 0.338 |
|  |  | No | 0.02 | **(0.01, 0.02)***** | Ref (0.00) | 0.00 | (-0.01, 0.01) | 0.00 | (-0.01, 0.01) | 0.01 | **(0.01, 0.02)**** | 0.513 | 0.002 |  |
|  | Model 4 | Yes | 0.01 | (0.00, 0.02) | Ref (0.00) | 0.00 | (-0.01, 0.01) | 0.00 | (-0.01, 0.01) | 0.01 | (0.00, 0.02) | 0.427 | 0.044 | 0.356 |
|  |  | No | 0.02 | **(0.01, 0.03)***** | Ref (0.00) | 0.00 | (-0.01, 0.01) | 0.00 | (-0.01, 0.01) | 0.01 | (0.00, 0.01) | 0.225 | 0.171 |  |
| Education |  |  |  |  |  |  |  |  |  |  |  |  |  |  |
|  | Model 1 | Above high school | 0.01 | (0.00, 0.02) | Ref (0.00) | 0.00 | (-0.01, 0.01) | 0.00 | (-0.01, 0.01) | 0.02 | **(0.01, 0.03)***** | < 0.001 | < 0.001 | 0.748 |
|  |  | Others | 0.02 | (0.00, 0.03) | Ref (0.00) | 0.01 | (0.00, 0.02) | 0.02 | **(0.01, 0.03)**** | 0.02 | **(0.02, 0.03)***** | 0.001 | < 0.001 |  |
|  | Model 2 | Above high school | 0.01 | (0.00, 0.02) | Ref (0.00) | 0.00 | (-0.01, 0.01) | 0.00 | (-0.01, 0.01) | 0.02 | **(0.01, 0.03)***** | 0.043 | 0.001 | 0.889 |
|  |  | Others | 0.02 | **(0.01, 0.03)**** | Ref (0.00) | 0.01 | (0.00, 0.02) | 0.01 | (0.00, 0.03) | 0.02 | **(0.01, 0.03)***** | 0.038 | < 0.001 |  |
|  | Model 3 | Above high school | 0.01 | (0.00, 0.02) | Ref (0.00) | 0.00 | (-0.01, 0.01) | -0.01 | (-0.01, 0.00) | 0.01 | (0.00, 0.02) | 0.228 | 0.005 | 0.706 |
|  |  | Others | 0.02 | (0.00, 0.03) | Ref (0.00) | 0.01 | (0.00, 0.02) | 0.01 | (0.00, 0.02) | 0.01 | **(0.01, 0.02)***** | 0.218 | < 0.001 |  |
|  | Model 4 | Above high school | 0.01 | (0.00, 0.02) | Ref (0.00) | 0.00 | (-0.01, 0.01) | -0.01 | (-0.02, 0.00) | 0.01 | (0.00, 0.02) | 0.680 | 0.113 | 0.668 |
|  |  | Others | 0.02 | **(0.01, 0.03)**** | Ref (0.00) | 0.01 | (0.00, 0.02) | 0.01 | (0.00, 0.02) | 0.01 | (0.00, 0.02) | 0.903 | 0.029 |  |
| Income |  |  |  |  |  |  |  |  |  |  |  |  |  |  |
|  | Model 1 | ≤ $55,000 | 0.01 | (0.00, 0.02) | Ref (0.00) | 0.01 | (0.00, 0.02) | 0.01 | (0.00, 0.02) | 0.03 | **(0.02, 0.04)***** | < 0.001 | < 0.001 | 0.696 |
|  |  | > $55,000 | 0.01 | (-0.01, 0.02) | Ref (0.00) | 0.00 | (-0.01, 0.01) | 0.00 | (-0.01, 0.01) | 0.02 | **(0.01, 0.03)***** | 0.013 | < 0.001 |  |
|  | Model 2 | ≤ $55,000 | 0.01 | (0.00, 0.02) | Ref (0.00) | 0.00 | (0.00, 0.01) | 0.01 | (-0.01, 0.02) | 0.02 | **(0.01, 0.03)***** | 0.005 | < 0.001 | 0.973 |
|  |  | > $55,000 | 0.01 | (0.00, 0.02) | Ref (0.00) | 0.00 | (-0.01, 0.01) | 0.00 | (-0.02, 0.01) | 0.01 | (0.00, 0.02) | 0.294 | 0.005 |  |
|  | Model 3 | ≤ $55,000 | 0.01 | (0.00, 0.02) | Ref (0.00) | 0.00 | (0.00, 0.01) | 0.00 | (-0.01, 0.01) | 0.01 | **(0.01, 0.02)***** | 0.029 | 0.001 | 0.888 |
|  |  | > $55,000 | 0.01 | (0.00, 0.02) | Ref (0.00) | 0.00 | (-0.01, 0.01) | 0.00 | (-0.02, 0.01) | 0.01 | (0.00, 0.02) | 0.763 | 0.054 |  |
|  | Model 4 | ≤ $55,000 | 0.01 | (0.00, 0.02) | Ref (0.00) | 0.00 | (-0.01, 0.01) | 0.00 | (-0.01, 0.01) | 0.01 | (0.00, 0.02) | 0.342 | 0.016 | 0.829 |
|  |  | > $55,000 | 0.01 | (0.00, 0.02) | Ref (0.00) | 0.00 | (-0.01, 0.01) | -0.01 | (-0.02, 0.01) | 0.00 | (-0.01, 0.01) | 0.139 | 0.804 |  |
| Sleep duration |  |  |  |  |  |  |  |  |  |  |  |  |  |  |
|  | Model 1 | ≤ 7 hours | 0.01 | (0.00, 0.02) | Ref (0.00) | 0.01 | (0.00, 0.02) | 0.02 | (0.00, 0.03) | 0.03 | **(0.02, 0.04)***** | < 0.001 | < 0.001 | 0.886 |
|  |  | > 7 hours | 0.01 | (0.00, 0.02) | Ref (0.00) | 0.00 | (-0.01, 0.01) | 0.00 | (-0.01, 0.01) | 0.02 | **(0.01, 0.03)***** | < 0.001 | < 0.001 |  |
|  | Model 2 | ≤ 7 hours | 0.01 | (0.00, 0.02) | Ref (0.00) | 0.00 | (-0.01, 0.01) | 0.01 | (0.00, 0.03) | 0.02 | **(0.01, 0.03)***** | 0.012 | < 0.001 | 0.975 |
|  |  | > 7 hours | 0.02 | **(0.01, 0.02)***** | Ref (0.00) | 0.00 | (-0.01, 0.01) | 0.00 | (-0.01, 0.01) | 0.01 | **(0.01, 0.02)***** | 0.157 | < 0.001 |  |
|  | Model 3 | ≤ 7 hours | 0.01 | (0.00, 0.02) | Ref (0.00) | 0.00 | (-0.01, 0.01) | 0.01 | (0.00, 0.03) | 0.02 | **(0.01, 0.02)***** | 0.027 | < 0.001 | 0.599 |
|  |  | > 7 hours | 0.01 | **(0.01, 0.02)**** | Ref (0.00) | 0.00 | (-0.01, 0.01) | -0.01 | (-0.02, 0.00) | 0.01 | (0.00, 0.02) | 0.810 | 0.010 |  |
|  | Model 4 | ≤ 7 hours | 0.01 | (0.00, 0.02) | Ref (0.00) | 0.00 | (-0.01, 0.01) | 0.01 | (0.00, 0.02) | 0.01 | (0.00, 0.02) | 0.362 | 0.019 | 0.615 |
|  |  | > 7 hours | 0.02 | **(0.01, 0.02)***** | Ref (0.00) | 0.00 | (-0.01, 0.01) | -0.01 | (-0.02, 0.00) | 0.00 | (0.00, 0.01) | 0.097 | 0.304 |  |
| Daily energy intake |  |  |  |  |  |  |  |  |  |  |  |  |  |  |
|  | Model 1 | Higher | 0.01 | (0.00, 0.02) | Ref (0.00) | 0.01 | (0.00, 0.02) | 0.01 | (0.00, 0.03) | 0.03 | **(0.02, 0.04)***** | < 0.001 | < 0.001 | 0.051 |
|  |  | Lower | 0.01 | (0.00, 0.02) | Ref (0.00) | 0.00 | (-0.01, 0.01) | 0.00 | (-0.01, 0.02) | 0.02 | **(0.02, 0.03)***** | < 0.001 | < 0.001 |  |
|  | Model 2 | Higher | 0.01 | (0.00, 0.02) | Ref (0.00) | 0.01 | (0.00, 0.02) | 0.00 | (-0.01, 0.02) | 0.02 | **(0.01, 0.03)***** | 0.470 | 0.001 | 0.072 |
|  |  | Lower | 0.01 | (0.00, 0.02) | Ref (0.00) | 0.00 | (-0.01, 0.01) | 0.00 | (-0.01, 0.01) | 0.01 | **(0.01, 0.02)***** | 0.012 | < 0.001 |  |
|  | Model 3 | Higher | 0.01 | (0.00, 0.02) | Ref (0.00) | 0.01 | (0.00, 0.02) | 0.00 | (-0.01, 0.01) | 0.02 | **(0.01, 0.03)**** | 0.742 | 0.003 | 0.068 |
|  |  | Lower | 0.01 | (0.00, 0.02) | Ref (0.00) | 0.00 | (-0.01, 0.01) | 0.00 | (-0.01, 0.01) | 0.01 | **(0.01, 0.02)***** | 0.023 | < 0.001 |  |
|  | Model 4 | Higher | 0.02 | **(0.01, 0.03)**** | Ref (0.00) | 0.01 | (-0.01, 0.02) | 0.00 | (-0.01, 0.01) | 0.01 | (0.00, 0.02) | 0.230 | 0.071 | 0.076 |
|  |  | Lower | 0.01 | (0.00, 0.02) | Ref (0.00) | 0.00 | (-0.01, 0.01) | 0.00 | (-0.01, 0.01) | 0.01 | (0.00, 0.01) | 0.842 | 0.127 |  |
| Healthy eating index-2015 |  |  |  |  |  |  |  |  |  |  |  |  |  |  |
|  | Model 1 | < 30.53 | 0.01 | (0.00, 0.02) | Ref (0.00) | 0.00 | (-0.01, 0.01) | 0.01 | (-0.01, 0.02) | 0.02 | **(0.01, 0.03)***** | < 0.001 | < 0.001 | 0.476 |
|  |  | ≥ 30.53 | 0.01 | (0.00, 0.02) | Ref (0.00) | 0.01 | (0.00, 0.02) | 0.01 | (0.00, 0.02) | 0.03 | **(0.02, 0.04)***** | < 0.001 | < 0.001 |  |
|  | Model 2 | < 30.53 | 0.01 | (0.00, 0.02) | Ref (0.00) | -0.01 | (-0.01, 0.01) | 0.00 | (-0.01, 0.01) | 0.01 | (0.00, 0.02) | 0.187 | 0.007 | 0.892 |
|  |  | ≥ 30.53 | 0.01 | **(0.01, 0.02)**** | Ref (0.00) | 0.01 | (0.00, 0.02) | 0.00 | (-0.01, 0.01) | 0.02 | **(0.01, 0.03)***** | 0.017 | < 0.001 |  |
|  | Model 3 | < 30.53 | 0.01 | (0.00, 0.02) | Ref (0.00) | -0.01 | (-0.01, 0.00) | 0.00 | (-0.01, 0.01) | 0.01 | (0.00, 0.02) | 0.475 | 0.046 | 0.925 |
|  |  | ≥ 30.53 | 0.01 | (0.00, 0.02) | Ref (0.00) | 0.01 | (0.00, 0.02) | 0.00 | (-0.01, 0.01) | 0.02 | **(0.01, 0.03)***** | 0.047 | < 0.001 |  |
|  | Model 4 | < 30.53 | 0.01 | (0.00, 0.02) | Ref (0.00) | -0.01 | (-0.02, 0.00) | 0.00 | (-0.01, 0.01) | 0.00 | (-0.01, 0.01) | 0.726 | 0.350 | 0.854 |
|  |  | ≥ 30.53 | 0.02 | **(0.01, 0.03)**** | Ref (0.00) | 0.01 | (0.00, 0.01) | 0.00 | (-0.01, 0.01) | 0.01 | (0.00, 0.02) | 0.836 | 0.023 |  |
| Dietary supplements use |  |  |  |  |  |  |  |  |  |  |  |  |  |  |
|  | Model 1 | Yes | 0.01 | (0.00, 0.02) | Ref (0.00) | 0.00 | (-0.01, 0.01) | 0.01 | (0.00, 0.02) | 0.02 | **(0.01, 0.03)***** | < 0.001 | < 0.001 | 0.260 |
|  |  | No | 0.01 | (0.00, 0.02) | Ref (0.00) | 0.01 | (0.00, 0.02) | 0.01 | (-0.01, 0.02) | 0.03 | **(0.02, 0.03)***** | < 0.001 | < 0.001 |  |
|  | Model 2 | Yes | 0.01 | (0.00, 0.02) | Ref (0.00) | 0.00 | (-0.01, 0.01) | 0.00 | (-0.01, 0.01) | 0.01 | **(0.01, 0.02)**** | 0.131 | 0.002 | 0.202 |
|  |  | No | 0.01 | (0.00, 0.02) | Ref (0.00) | 0.01 | (0.00, 0.02) | 0.00 | (-0.01, 0.01) | 0.02 | **(0.01, 0.02)***** | 0.018 | < 0.001 |  |
|  | Model 3 | Yes | 0.01 | (0.00, 0.02) | Ref (0.00) | 0.00 | (-0.01, 0.01) | 0.00 | (-0.01, 0.01) | 0.01 | (0.00, 0.02) | 0.464 | 0.018 | 0.123 |
|  |  | No | 0.01 | (0.00, 0.02) | Ref (0.00) | 0.01 | (0.00, 0.01) | 0.00 | (-0.01, 0.01) | 0.02 | **(0.01, 0.02)***** | 0.062 | < 0.001 |  |
|  | Model 4 | Yes | 0.01 | (0.00, 0.02) | Ref (0.00) | 0.00 | (-0.01, 0.01) | 0.00 | (-0.01, 0.01) | 0.01 | (0.00, 0.02) | 0.363 | 0.286 | 0.092 |
|  |  | No | 0.01 | (0.00, 0.02) | Ref (0.00) | 0.00 | (-0.01, 0.01) | 0.00 | (-0.01, 0.01) | 0.01 | (0.00, 0.02) | 0.762 | 0.014 |  |
| Daily eating frequency |  |  |  |  |  |  |  |  |  |  |  |  |  |  |
|  | Model 1 | < 4.00 times | 0.02 | **(0.01, 0.03)**** | Ref (0.00) | 0.01 | (0.00, 0.02) | 0.00 | (-0.01, 0.02) | 0.02 | **(0.01, 0.03)***** | 0.001 | < 0.001 | 0.001 |
|  |  | ≥ 4.00 times | 0.01 | (0.00, 0.02) | Ref (0.00) | 0.00 | (-0.01, 0.01) | 0.01 | (-0.01, 0.02) | 0.03 | **(0.01, 0.04)***** | 0.393 | 0.001 |  |
|  | Model 2 | < 4.00 times | 0.02 | (0.00, 0.03) | Ref (0.00) | 0.00 | (-0.01, 0.01) | 0.00 | (-0.01, 0.01) | 0.01 | (0.00, 0.02) | 0.083 | 0.002 | 0.003 |
|  |  | ≥ 4.00 times | 0.01 | (0.00, 0.02) | Ref (0.00) | 0.00 | (-0.01, 0.01) | 0.00 | (-0.01, 0.02) | 0.02 | **(0.01, 0.04)**** | 0.980 | 0.003 |  |
|  | Model 3 | < 4.00 times | 0.01 | (0.00, 0.03) | Ref (0.00) | 0.00 | (-0.01, 0.01) | 0.00 | (-0.01, 0.01) | 0.01 | (0.00, 0.02) | 0.177 | 0.010 | 0.005 |
|  |  | ≥ 4.00 times | 0.01 | (0.00, 0.02) | Ref (0.00) | 0.00 | (-0.01, 0.01) | 0.00 | (-0.01, 0.01) | 0.02 | **(0.01, 0.03)**** | 0.786 | 0.009 |  |

^a^The variables adjusted in each model were the factors mentioned above except the stratification variables.

^b^Data were listed as the weighted beta estimates and 95% confidence intervals, with *p < 0.05, **p < 0.01, ***p < 0.001.

^c^Q, quintile.

^d^Ref, reference.

^e^Tests for trends based on the variables containing the median values for each quartile.

^f^*P*_test_ was the result of Bonfreni correction.

^g^Multiplicative interaction was assessed by adding interaction terms to the models.

**Supplementary figure 1.** Matrix displaying the associations between chronological age and predicted age metrics (Pearson correlation).


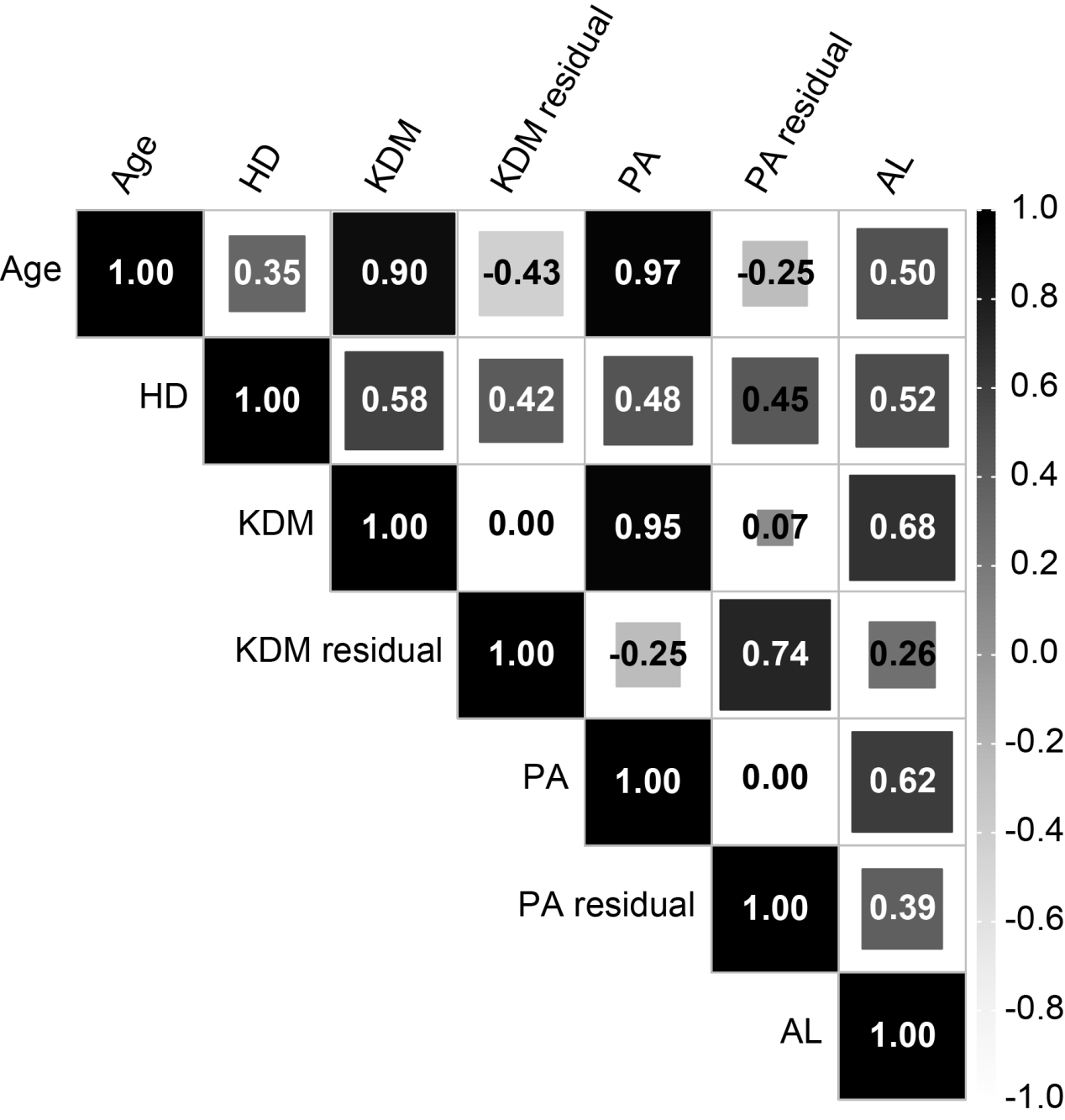


**Supplementary figure 2.** Scatter plot illustrating the correlation between DEF and NFD (Pearson correlation).


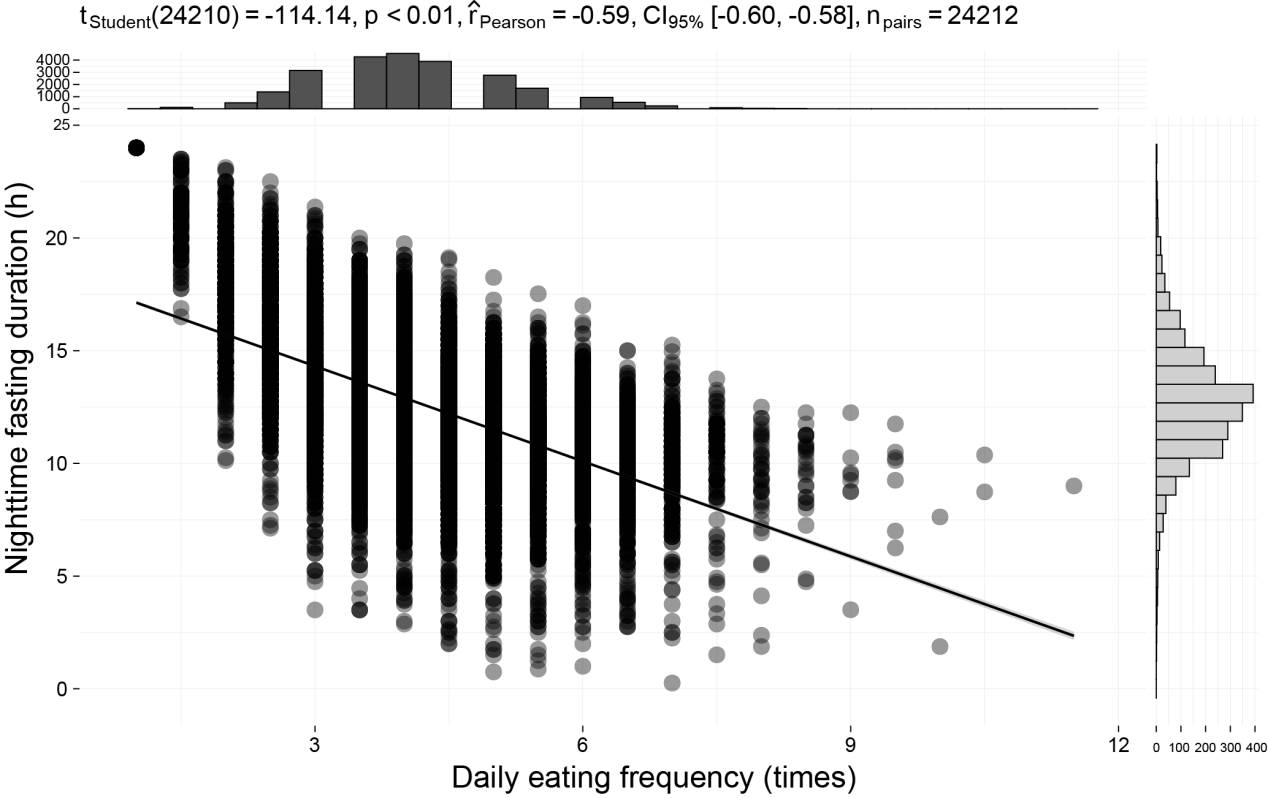

Supplement: Supplementary file 3 — Additional file 3: Supplementary Table 1. Components of biological age metrics by quintiles of DEF. Supplementary Table 2. Components of biological age metrics by quintiles of NFD. Supplementary Table 3. Differences in the baseline characteristics of participants categorized by quintiles of NFD. Supplementary Table 4. Associations of DEF with predicted age metrics. Supplementary Table 5. Associations of NFD with predicted age metrics. Supplementary Table 6. Association of DEF with Ln-transformed HD stratified by variables of interest. Supplementary Table 7. Association of DEF with KDM residual stratified by variables of interest. Supplementary Table 8. Association of DEF with PA residual stratified by variables of interest. Supplementary Table 9. Association of DEF with AL stratified by variables of interest. Supplementary Table 10. Association of NFD with Ln-transformed HD stratified by variables of interest. Supplementary Table 11. Association of NFD with KDM residual stratified by variables of interest. Supplementary Table 12. Association of NFD with PA residual stratified by variables of interest. Supplementary Table 13. Association of NFD with AL stratified by variables of interest. Supplementary Fig. 1. Matrix displaying the associations between chronological age and predicted age metrics (Pearson correlation). Supplementary Fig. 2. Scatter plot illustrating the correlation between DEF and NFD (Pearson correlation). [file 12966_2024_1654_MOESM3_ESM.docx]
